# Sex-specific associations of depression with inflammatory markers: a systematic review and meta-analysis

**DOI:** 10.1038/s41398-026-04139-7

**Published:** 2026-06-02

**Authors:** Yvette Z. Szabo, Kristen Nishimi, Mary Smirnova, Andrea Niles, Anna Skovgaard Lerche, Hao-An Tsai, Sasha Afroz, Nora Huey, Maya Mathur, Aoife O’Donovan

**Affiliations:** 1 Department of Psychology, California State University, Los Angeles, Los Angeles, CA USA; 2 Mental Health Service, San Francisco Veterans Affairs Health Care System, San Francisco, CA USA; 3 Department of Psychiatry and Behavioral Sciences, University of California San Francisco, San Francisco, CA USA; 4 Department of Psychology, University of Nevada Reno, Reno, NV USA; 5 School of Medicine, Georgetown University, Washington, DC USA; 6 School of Nursing and Health, University of San Francisco, San Francisco, CA USA; 7 Quantitative Sciences Unit and Department of Pediatrics, Stanford University, Palo Alto, CA USA

**Keywords:** Depression, Biomarkers

## Abstract

**Introduction:**

Converging evidence implicates elevated inflammation in the pathophysiology of depression and its medical comorbidities. Despite known sex differences in both inflammatory activity and depression, a dedicated large-scale review of these differences is lacking.

**Methods:**

We conducted a sex-stratified systematic review and meta-analysis of studies examining associations between depression and blood-based inflammatory markers in adult humans. Searches of PubMed, PsycINFO, and Scopus, and reference treeing, yielded 124 eligible papers including data from 423,421 participants (53% female). Analyses focused on depression and inflammatory markers C-reactive protein (CRP), interleukin (IL)-6, tumor necrosis factor (TNF)-α, IL-1β, and fibrinogen. Sex-stratified meta-analyses and meta regressions for the effects of sex were conducted, separately for cross-sectional and longitudinal designs.

**Results:**

Cross-sectionally, depression was modestly associated with higher levels of all markers combined in both females (Cohen’s *d* = 0.06, 95% CI [0.03–0.10]) and males (Cohen’s *d* = 0.14, 95% CI [0.09–0.19]). Depression was associated with elevated IL-6 in females, and elevated CRP and IL-6 in males. Longitudinally, elevated IL-6 was associated with subsequent depression among females and elevated CRP was associated with depression in males, whereas depression was not associated with subsequent inflammation in either sex.

**Conclusion:**

Overall, results indicated modest associations between depression and elevated inflammation in both sexes, with slightly stronger associations in males. Associations between specific inflammatory markers (i.e., IL-6 in females and CRP in males) and subsequent depression differed by sex. These findings highlight the cross-sex relevance of inflammation in depression, and subtle sex differences in directionality and specific markers.

## Introduction

Depression is among the most common psychiatric disorders [1], with an average annual prevalence of 4.4% worldwide [2], and a leading cause of disability [1]. Strikingly, women are twice as likely as men to develop depression [2–5]. Antidepressant medications are widely and increasingly prescribed, with women receiving about 68% of antidepressant prescriptions [6, 7]. Despite their wide use, antidepressants can have significant side effects and are not effective for all patients [8]. Thus, novel interventions for depression are desperately needed. Elevated inflammation may be an important new treatment target for depression and has even been proposed as a signifier for depression in the Diagnostic and Statistical Manual for Mental Disorders -VI [9]. Substantial preclinical and clinical evidence implicates elevated inflammation in the development of depressive symptoms [10–13], and in medical comorbidity associated with depression [14–17]. However, despite known sex differences in both inflammatory activity and depression, there has been no large-scale systematic meta-analysis focusing on sex differences in relationships between depression and inflammatory markers.

Multiple factors related to sex (e.g., biological attributes) and gender (e.g., identity-based roles and behaviors) may contribute to differences in relationships between depression and inflammatory markers. First, sex differences in immune function are evident across species, with females exhibiting heightened immune responses compared to males [18]. While this pattern can reduce vulnerability to infection, it also places females at higher risk for some types of immunopathology, including autoimmune disorders [18]. Second, the underlying neurobiology of depression may differ by sex, with experimental studies indicating that acute inflammation may yield greater increases in depressive symptoms in females [19, 20]. Third, risk factors for depression differ by gender, with gender roles, stress exposure, and social desirability potentially contributing to differences in risk for depression [21, 22]. Thus, immunological, neurobiological, and sociocultural factors may drive sex differences in neural and psychological responses to inflammation. These differences have important implications for both theory and clinical research. First, if there are sex differences in associations between depression and inflammation, research progress will be hampered if we fail to include sex in our conceptual models. Second, sex differences in associations between inflammation and depression have important implications for clinical research. For example, strong sex differences could support sex stratification in clinical trials of anti-inflammatory interventions for depression, the development of different targeted interventions for females and males, and consideration of sex differences in studies examining mechanisms accounting for increased medical morbidity in depressed individuals. Thus, a better understanding of sex differences in associations between depression and inflammation can enhance theory and clinical research.

Large-scale meta-analyses have reported elevated levels of the inflammatory markers C-reactive protein (CRP), interleukin-6 (IL-6), and tumor necrosis factor-alpha (TNF-α) in depressed individuals versus controls [23–26]. Several of these meta-analyses reported no evidence of moderation by sex [23–25, 27], but these meta-analyses were not specifically designed to address sex differences. One prior meta-analysis specifically addressing sex differences among samples with clinical depression reported that IL-6 and CRP were significantly higher in women but not men with depression, in a small selection of 23 studies with sex-stratified samples (5459 males, 7364 females in the CRP analysis) [28]. Thus, sex differences in the depression-inflammation relationship may exist. Extending this prior work, we conducted a dedicated large-scale systematic review and meta-analysis of sex differences in depression-inflammation associations encompassing clinical and non-clinical samples using cross-sectional and longitudinal designs.

Here, we present a comprehensive meta-analysis and meta-regression of studies examining the relationship between depression and inflammation. We report sex-stratified meta-analyses of depression and inflammation associations, and meta-regressions estimating sex moderation. To examine the robustness of observed associations, we separately examined associations from cross-sectional and longitudinal studies, unadjusted and confounder-adjusted estimates, samples with clinical and subclinical depression, and samples with and without medical illness. We hypothesized that depression would be associated with inflammatory markers in both sexes, and the strength of the relationship would differ between females and males. Despite some theory and previous research suggesting depression-inflammation associations may be stronger in females, other studies have reported associations stronger in males for some markers [29]. Thus, we did not have directionally specific hypotheses regarding sex effects or individual markers.

## Method

This review follows Preferred Reporting Items for Systematic Reviews and Meta-Analyses (PRISMA) guidelines [30] and was preregistered with Open Science Framework (https://osf.io/wx8bz). The larger project examined the relationship between depression or anxiety and inflammation, but we only report on depression here. The Supplement includes a summary of and justifications for revisions, deviations, and post hoc analyses.

### Search methods

PubMed, PsycINFO, and Scopus were searched from inception through January 2023 for papers examining depression and inflammation associations (search terms in Supplement). We also utilized reference treeing to identify additional papers.

### Eligibility determination

**Fig. 1:**
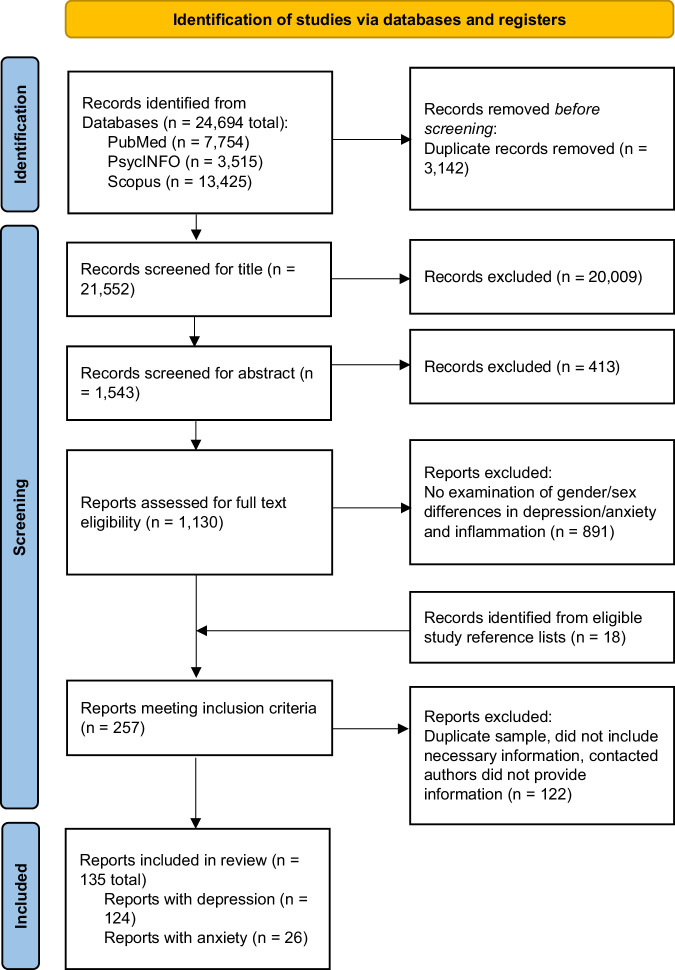
PRISMA 2020 flow diagram for new systematic reviews which included searches of databases and registers only. *From:* Page MJ, McKenzie JE, Bossuyt PM, Boutron I, Hoffmann TC, Mulrow CD, et al. The PRISMA 2020 statement: an updated guideline for reporting systematic reviews. BMJ 2021;372:n71. 10.1136/bmj.n71.

Papers were included for eligibility in three phases: (1) title, (2) abstract, and (3) full-text screening. Authors (KN, MS, SA, HT, AN) reviewed the titles, then abstracts of potentially relevant articles (Fig. 1). At the title and abstract screening phases, papers were evaluated for presence of the following criteria: inflammatory marker, depression measure, published in English, published in a peer-reviewed journal, and including only adults over the age of 18. Depression measure could include self-reported symptoms, clinical interviews, or other depression measure (see Table S3 for measures), and there were no specific restrictions related to clinical depression diagnoses. The paper did not need to mention sex/gender differences at this point because the title and abstract might not include this information. In the full-text phase, authors (authors above plus YS and AS) examined the entire paper to search for two additional inclusion criteria: 1) the analyses examined sex/gender differences or the study enrolled only one sex/gender (studies including mostly one sex/gender plus two or fewer individuals of the other sex/gender were considered single sex/gender); and 2) statistical estimates that could be expressed on the standardized mean difference scale were reported for our question of interest (e.g., the paper examined the association between the presence/absence or the severity of depression with inflammatory markers for each sex separately or for one sex). If sex difference analyses were conducted but data was not reported, we contacted the authors by email. We included only CRP, IL-6, TNF-α, IL-1β, and fibrinogen due to an insufficient number of studies examining other markers leading to imprecise variance estimates or unstable quantification of heterogeneity (i.e., less than three separate estimates per marker for cross-sectional analyses) [31]. In each phase of review, each exclusion decision was reviewed and confirmed by a second reviewer. Discrepancies were resolved by discussion or with a third reviewer (AOD) when necessary. See Supplement for full inclusion/exclusion criteria.

### Data extraction

For each paper, authors recorded statistics needed to compute Cohen’s *d*, corresponding to the standardized mean difference (SMD) between a binary variable (e.g., having versus not having depression) and standardized levels of a continuous variable (e.g., marker levels). For papers reporting multiple adjusted models, we extracted information from unadjusted, minimally adjusted, and most adjusted models. To retain all possible papers and standardize reported statistics, we converted and calculated effect estimates as needed and prioritized available data (see Supplement for details). We also recorded sample size, sex distribution, study design, marker, blood fraction, depression measure, and covariate adjustment.

### Study quality and risk of bias

Risk of bias was assessed at paper and analysis levels. For papers, reviewers assessed quality and risk of bias using the Methodological Index for Non-Randomized Studies (MINORS) criteria [32], adapted based on other guides and methodological standards for inflammation (see Table S2 for criteria) [33]. We attempted to reduce publication bias by contacting authors when sex differences were assessed but not reported (e.g., reported testing sex moderation).We then conducted sensitivity analyses for publication bias to statistically and visually estimate whether our primary findings were influenced by selective reporting of estimates or studies.

### Statistical analysis

We fit random-effects meta-regressions in R with point estimates on the Cohen’s *d* scale using robust variance estimation (RVE) [34, 35].

### Main analyses

Separate sex-stratified meta-analyses were conducted for all markers combined and each individual marker with three or more estimates, stratified by study design (i.e., cross-sectional, longitudinal with depression and subsequent inflammation, longitudinal with inflammation and subsequent depression) to reduce bias due to study design differences [36]. We considered all markers combined within a model to index a broad pro-inflammatory milieu, then examined each individual marker to identify specific depression-marker associations [37]. When a study reported models with different levels of adjustment, the most adjusted estimate was used in this main analytic model to analyze effects adjusted for potential confounders. To directly estimate the difference in depression-inflammation associations between sexes, we also conducted meta-regressions for all markers combined and each individual marker, stratified by study design combining both females and males with an indicator variable for sex (male as referent).

For each of the primary meta-analyses, we report the pooled point estimate and the estimated standard deviation of population effects (τ) along with their 95% confidence intervals and p-values. We report both uncorrected inference and inference that has been corrected for multiple comparisons using the Holm-Bonferroni method [38]. Since basic meta-analyses only characterize the mean of a potentially heterogeneous distribution of effects, we also estimated the percentage of population effects that were positive (i.e., any association in the expected direction) and that were stronger than a magnitude of Cohen’s *d* = 0.2 [39, 40].

### Sensitivity analyses

Several sets of analyses assessed sensitivity to unmeasured confounding (including differences in levels of covariate adjustment, populations with medical illness versus not, and clinical diagnoses of depression versus not) [41, 42], and to publication bias (details in Supplement).

## Results

### Search results and study selection

Database searches yielded 24,694 papers (21,552 unique). As shown in Fig. 1, all 21,552 were title screened, 1543 were abstract screened, and 1130 were reviewed in full. Of these, 239 met inclusion criteria and an additional 18 were added through reference treeing. These 257 papers were reviewed for non-independent samples, removing 41 papers by prioritizing availability of data, largest sample, and then most recent. During data extraction, 81 papers were excluded due to missing necessary data or lack of study author response resulting in 124 included papers for depression (an additional *n* = 11 reported on anxiety only).

### Study and participant characteristics

The 124 eligible papers (*n* = 423,421) contributed one or more estimates (Table S3). Papers could contribute multiple estimates (e.g., female and male estimates, estimates stratified by participant characteristics); thus, *k* estimates were often larger than *k* papers for any given model (e.g., Table 1 all markers combined for females included k = 147 estimates from k = 85 papers).

**Table 1:** Meta-analytic results for cross-sectional studies: depression-biomarker associations separately among females and males.

	k_pap._	k_est._	n_obs._	Cohen’s *d* [95% CI]	p	p_Bon._	τ^2^	df	% positive	% positive and *d* > 0.20	% negative and *d* < −0.20
Females											
All Markers	**85**	**147**	**211,926**	**0.06** [**0.03, 0.10]**	**0.001**	**0.003**	0.07	49.06	91 [78, 100]	2 [0, 16]	0
CRP	60	69	190,865	0.01 [−0.01, 0.03]	0.144	1.000	0.01	5.64	94 [84, 100]	0	0
IL-6	**40**	**41**	**11,565**	**0.09** [**0.02, 0.17]**	**0.014**	0.290	0.17	29.73	76 [52, 88]	22 [0, 41]	2 [0, 10]
TNF-α	22	23	5550	0.15 [−0.04, 0.35]	0.119	1.000	0.21	16.53	78 [0, 96]	30 [0, 70]	0
L-1β	11	11	2708	0.41 [−0.36, 1.18]	0.256	1.000	0.49	9.32	64 [0, 91]	27 [0, 55]	0
Fibrinogen	3	3	1238	0.23 [−0.76, 1.22]	0.419	1.000	0.47	2.00	100	100	0
Males											
All Markers	**71**	**108**	**206,007**	**0.14** [**0.09, 0.19]**	**<0.001**	**<0.001**	0.11	47.48	89 [80, 94]	25 [5, 41]	0
CRP	**55**	**58**	**176,475**	**0.08** [**0.05, 0.11]**	**<0.001**	**<0.001**	0.06	31.83	97 [80, 100]	12 [0, 43]	0
IL-6	**27**	**27**	**18,272**	**0.13** [**0.04, 0.22]**	**0.008**	0.151	0.18	20.05	81 [54, 93]	37 [4, 59]	0
TNF-α	11	11	6083	0.30 [−0.37, 0.98]	0.337	1.000	0.40	9.18	73 [0, 100]	27 [0, 64]	0
IL-1β	6	6	1905	1.84 [−1.10, 4.78]	0.168	1.000	1.33	4.95	100	100	0
Fibrinogen	6	6	3272	0.27 [−0.21, 0.75]	0.207	1.000	0.29	4.74	100	100	0

k_pap._ = number of papers contributing to each model; k_est_. = number of total estimates (some papers contributed more than one estimate to each model); n_obs._ = total sample size (number of individual observations in each model). *p*_Bon_*. =* Bonferroni adjusted p value. %’s refer to the estimated percentage of population effects. Results in bold indicate *p* < 0.05 and italics indicate 0.05 ≤ *p* < *0.10*.

Regarding sex, 98 papers included females (*n* = 222,507) and 85 included males (*n* = 200,914), with sex-stratified sample sizes ranging from 6 to 81,964. Depression measures largely included validated symptom measures or clinical interviews, and samples reflected community samples and clinical populations. Markers were derived from serum (67 papers) or plasma (43 papers) with one paper including either serum or plasma, and 13 not reporting fraction. Regarding specific markers, 92 papers reported CRP (64 hsCRP), 58 IL-6, 27 TNF-α, 12 IL-1β, and eight fibrinogen.

### Study quality

Quality was assessed for the paper in general and for sex-specific analyses (Table S4). Papers ranged in risk of bias, with average quality of 8.5 (range 2–13) in general and 7.1 (range 1–12) for sex-specific analyses, out of possible 0–16. Most had stated aims, but fewer had sex-specific aims, and most adequately reported inclusion/exclusion criteria. Only one study reported pre-registration. Of the included papers, 92 were cross-sectional and 32 were longitudinal. Of the sex differences associations included in analyses, 102 papers contributed only cross-sectional estimates and 22 contributed longitudinal or prospective estimates. There was variability in data missingness and in the adequacy of reporting or handling of missing data. Two-thirds of studies failed to adequately report or reported poor assay reliability. Almost 70% of studies reported main effects adjusted for confounding, though a lower 49% of sex-specific estimates were adequately adjusted. Sample sizes ranged widely, with only 40% of included studies powered for medium effect sizes.

### Meta-analyses for sex differences in depression-inflammation associations

Meta-analysis results for sex-stratified depression-marker associations are presented in Table 1 (cross-sectional), Table 2 (longitudinal), and Fig. 2 and Figs. S1–S22.

**Fig. 2:**
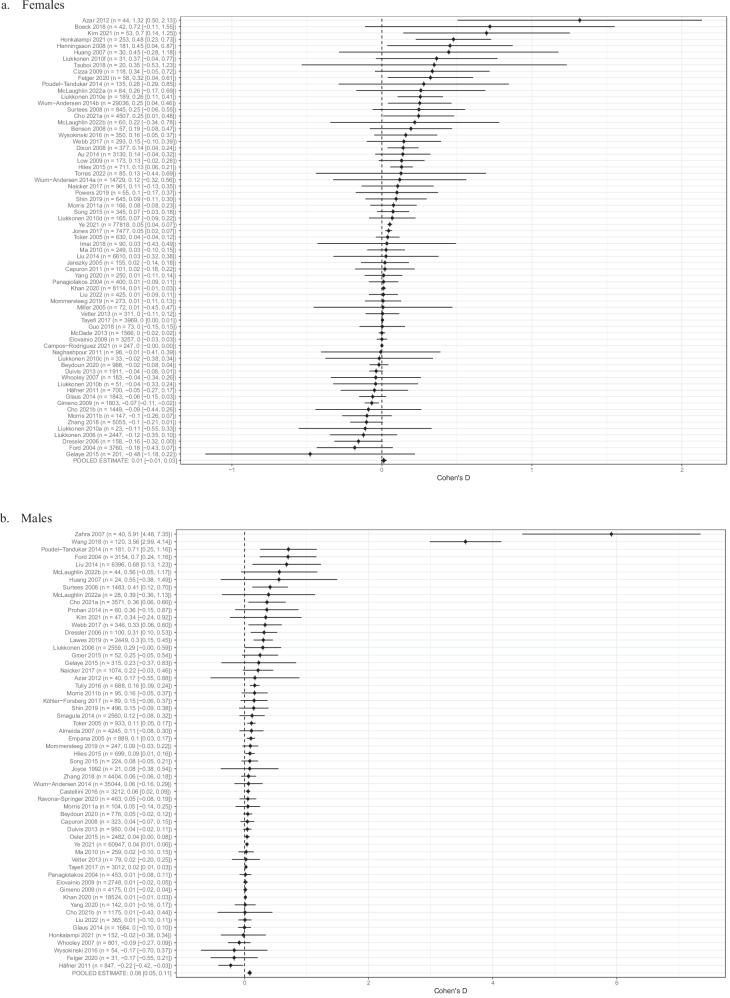
Forest plots of depression and CRP in cross-sectional studies among females and males. **a** Forest plot of depression and CRP in cross-sectional studies among females (k_papers _= 60; k_estimates_ = 69; n_observations_ = 190,865). **b** Forest plot of depression and CRP in cross-sectional studies among males (k_papers _= 55; k_estimates_ = 58; n_observations_ = 176,475).

**Table 2:** Meta-analytic results for longitudinal studies: depression-biomarker associations separately among females and males.

	k_pap._	k_est._	n_obs._	Cohen’s *d* [95% CI]	p	p_Bon._	τ^2^	df	% positive	% positive and *d* > 0.20	% negative and *d* < −0.20
Depression and subsequent Inflammation											
Females											
All Markers	6	9	14,083	0.08 [−0.14, 0.31]	0.381	1.000	0.12	4.93	100	0	0
CRP	6	6	12,492	0.07 [−0.12, 0.27]	0.382	1.000	0.11	4.90	100	0	0
IL-6	2	2	1388	0.32 [−3.35, 3.99]	0.467	1.000	0.41	1.00	100	100	0
Males											
All Markers	7	15	10,602	0.04 [−0.02, 0.11]	0.149	1.000	0.09	5.83	60 [3, 80]	13 [0, 27]	0
CRP	7	7	5006	0.10 [−0.03, 0.22]	0.109	1.000	0.11	5.90	100	0	0
IL-6	5	5	4022	0.01 [−0.08, 0.11]	0.702	1.000	0.05	3.62	100	0	0
Inflammation and subsequent Depression											
Females											
All Markers	**12**	**17**	**29,624**	**0.03** [**0.00, 0.06]**	**0.039**	0.311	0.03	6.59	94 [59, 100]	0	0
CRP	11	11	24,702	0.02 [−0.01, 0.04]	0.122	1.000	0.02	5.86	91 [0, 100]	0	0
IL-6	**5**	**5**	**4824**	**0.05** [**0.00, 0.10]**	**0.040**	0.524	0.00	2.42	100	0	0
Males											
All Markers	*11*	*18*	*19,830*	*0.07* [−*0.01, 0.15]*	*0.091*	0.728	0.08	7.45	83 [33, 100]	0	0
CRP	**10**	**10**	**14,043**	**0.06** [**0.00, 0.13]**	**0.050**	0.648	0.05	5.27	100 [40, 100]	0	0
IL-6	6	6	4568	0.03 [−0.05, 0.10]	0.388	1.000	0.05	3.84	100	0	0

k_pap._ = number of papers contributing to each model; k_est._ = number of total estimates (some papers contributed more than one estimate to each model); n_obs._ = total sample size (number of individual observations in each model). *p*_Bon_*. =* Bonferroni adjusted p value. %’s refer to the estimated percentage of population effects. Results in bold indicate *p* < 0.05 and italics indicate 0.05 ≤ *p* < *0.10*.

In cross-sectional sex-stratified models, among both females and males, depression was associated with higher levels of all markers combined, with modest effect sizes (female Cohen’s *d* = 0.06 [0.03, 0.10], male Cohen’s *d* = 0.14 [0.09, 0.19]) and relatively high heterogeneity between included studies (female τ^2^ = 0.07, male τ^2^ = 0.11). Among individual markers, depression was associated only with elevated IL-6 in females, but with higher levels of both CRP and IL-6 in males. There was also heterogeneity between studies for both sexes across individual marker models.

In longitudinal sex-stratified models with depression and subsequent inflammation, all estimates were positive, but none statistically significant. For inflammation and subsequent depression, all estimates were positive. Among females, higher levels of all markers combined was associated with subsequent depression (Cohen’s *d* = 0.03, [0.00, 0.06]), with only IL-6 significantly associated with subsequent depression among individual markers (Cohen’s *d* = 0.05 [0.00, 0.10]). Conversely, all markers combined was marginally associated with subsequent depression among males (Cohen’s *d* = 0.07, [−0.01, 0.15]), with only CRP significantly associated with subsequent depression among individual markers (Cohen’s *d* = 0.06 [0.00, 0.13]).

Meta-regressions for the effect of sex are presented in Table 3 (cross-sectional) and Table 4 (longitudinal), whereby the estimate for females reflects the difference in associations between depression and markers between males and females. In cross-sectional models, results indicate a significant depression-inflammation association for all markers combined, CRP, and IL-6 among males. Additionally, for all makers combined and CRP, the depression-marker associations were significantly stronger in males compared to females. In longitudinal models, we observed marginally significant effects for all markers combined and CRP, suggesting marginal depression-inflammation associations for males. There were no significant moderating effects, indicating that longitudinal depression-marker associations did not differ by sex.

**Table 3:** Meta-regression results for effects of sex in cross-sectional studies: depression-biomarker associations.

	k_pap._	k_est._	n_obs._	Predictor	Cohen’s *d* [95% CI]	p	τ^2^	I^2^	df
All Markers	107	255	417,933	Male	**0.14** [**0.09, 0.20]**	**<0.001**	0.01	90.02	40.31
				Female	−**0.06** [−**0.12, 0.00]**	**0.049**			64.87
CRP	75	127	367,340	Male	**0.07** [**0.05, 0.10]**	**<0.001**	0.00	81.39	20.32
				Female	−**0.05** [−**0.08**, −**0.02]**	**0.002**			27.52
IL-6	51	68	29,837	Male	**0.15** [**0.05, 0.24]**	**0.005**	0.03	87.40	17.16
				Female	−0.05 [−0.17, 0.07]	0.424			31.89
TNF-α	24	34	11,633	Male	0.13 [−0.19, 0.46]	0.362	0.05	90.20	7.15
				Female	0.00 [−0.25, 0.25]	0.981			12.12
IL-1β	12	17	4613	Male	1.96 [−1.17, 5.10]	0.168	0.50	95.03	4.92
				Female	−1.66 [−4.30, 0.99]	0.183			7.28
Fibrinogen	6	9	4510	Male	0.31 [−0.25, 0.87]	0.206	0.11	96.15	4.47
				Female	−0.08 [−0.86, 0.69]	0.759			2.95

Estimates reflect dummy coding where the coefficient for male refers to the average effect among males (the intercept), and the coefficient for female refers to the average difference in effect between males and females. k_pap._ = number of papers contributing to each model; k_est._. = number of total estimates (some papers contributed more than one estimate to each model); n_obs._ = total sample size (number of individual observations in each model). Results in bold indicate *p* < 0.05 and italics indicate 0.05 ≤ *p* < *0.10*.

**Table 4:** Meta-regression results for effects of sex in longitudinal studies: depression-biomarker associations.

	k_pap._	k_est._	n_obs._	Predictor	Cohen’s *d* [95% CI]	p	τ^2^	I^2^	df
Depression and subsequent Inflammation									
All Markers	9	24	24,685	Male	*0.06* [−*0.02, 0.14]*	*0.098*	0.01	90.49	5.19
				Female	0.00 [−0.16, 0.15]	0.973			7.30
CRP	9	13	17,498	Male	*0.11* [−*0.01, 0.24]*	*0.068*	0.01	89.60	5.17
				Female	−0.06 [−0.20, 0.07]	0.314			7.27
IL-6	5	7	5410	Male	0.03 [−0.05, 0.11]	0.339	0.03	93.33	3.61
				Female	0.28 [−1.61, 2.17]	0.474			1.45
Inflammation and subsequent Depression									
All Markers	15	35	49,454	Male	0.07 [−0.04, 0.17]	0.178	0.00	63.30	6.42
				Female	−0.04 [−0.14, 0.06]	0.426			9.71
CRP	14	21	38,745	Male	0.06 [−0.03, 0.15]	0.128	0.00	58.12	3.79
				Female	−0.04 [−0.12, 0.04]	0.236			7.05
IL-6	7	11	9392	Male	0.03 [−0.07, 0.12]	0.474	0.00	45.47	3.80
				Female	0.02 [−0.08, 0.12]	0.562			4.58

Estimates reflect dummy coding where the coefficient for male refers to the average effect among males (the intercept), and the coefficient for female refers to the average difference in effect between males and females. k_pap._ = number of papers contributing to each model; k_est._ = number of total estimates (some papers contributed more than one estimate to each model); n_obs._ = total sample size (number of individual observations in each model). Results in bold indicate *p* < 0.05 and italics indicate 0.05 ≤ *p* < *0.10*.

### Sensitivity analyses

#### Confounding control

E-values assess unmeasured confounding that would reduce observed results to null and represent a factor equivalent to an unmeasured confounder that affects both depression and inflammation on a risk ratio scale (Tables S5, S6). For example, to explain away the cross-sectional depression-all markers combined association among females, an unmeasured confounder would need to be associated with both depression and inflammation with a risk ratio of 1.31, but weaker confounds would not. Primary models relied on the most adjusted estimates available for each effect, thus E-values reflect estimates of residual confounding after adjustment. Observed E-values for the estimate ranged from 1.12 to 10.14, with most values ranging between ~1.2 and ~2.0 suggesting low to moderate unmeasured confounder associations could explain associations.

We also report the strength of unmeasured confounding required to reduce the proportion of true effects larger than *d* = 0.2 to below 10% (Table S5). For the cross-sectional association between depression and all markers combined among males, an unmeasured confounder would need to be associated with both depression and inflammation by an SMD of 1.46 or greater (observed range 1.00–2.77).

To assess how associations vary by confounding control, secondary meta-regressions included all estimates with a categorical covariate for confounding adjustment (unadjusted, minimally adjusted, or fully adjusted) as well as meta-analyses restricted to only unadjusted versus only adjusted estimates (Tables S7–S12). Among cross-sectional models, associations for adjusted models were smaller in magnitude than unadjusted models. In longitudinal models, no major differences were found between unadjusted versus adjusted associations in females or males.

A subset of studies (16%; *n* = 5152 females and *n* = 7318 males for cross-sectional models of all markers combined) included samples selected for medical illness or high risk for medical illness, including high cardiovascular disease (CVD) risk, CVD, congestive heart failure, coronary heart disease, ischemic heart disease, polycystic ovary syndrome, perimenopausal syndrome, type 2 diabetes, obstructive sleep apnea, overweight/obese, anorexia nervosa, rheumatoid arthritis, osteoarthritis, human immunodeficiency virus (HIV), and fibromyalgia. Results were generally similar for females in samples selected for medical illness versus not, while for males, depression-inflammation associations were stronger in non-medical illness compared to medical illness samples, with all markers combined associations twice as strong among non-medical illness males versus medical illness males (Tables S13–15). A subset of studies (28%; n = 60,476 females and n = 49,925 males for cross-sectional models of all markers combined) included samples with clinical depression. Estimates were stronger overall, though less precise, in cross-sectional models for both females and males in samples using clinical depression diagnoses versus not (Tables S16–19).

#### Publication bias

The selection model estimates the ratio of publishing affirmative versus non-affirmative results on average (Tables S5, S6). For cross-sectional associations of depression an all-markers, this model suggested there was publication bias in male samples (with affirmative results an estimated 2.25 times more likely to be published than non-affirmative results), but suggested no publication bias in female samples (estimated ratio 0.91).

Significance funnels for primary meta-analyses present the black diamond as the primary estimate and grey diamond reflecting worst-case publication bias-corrected estimate (Figs. S23–46). Close proximity of the diamonds indicates robustness to publication bias. There was some evidence of publication bias (e.g., cross-sectional models in females for TNF-α and fibrinogen).

Worst-case scenario estimates were calculated including only non-affirmative studies (Tables S5, S6); in these conservative estimates, cross-sectional models for females and males indicate an association for depression and all markers (and CRP specifically in males).

## Discussion

In this pre-registered meta-analysis of data from 423,421 participants (53% female) across 124 studies, we determined sex-specific associations between depression and inflammatory markers, and sex differences in the strength and direction of these associations. Cross-sectionally, depression was associated with elevated inflammatory activity in both sexes, as indexed by analyses of all inflammatory markers combined and IL-6. Among males, depression was additionally associated with elevated levels of CRP. Moreover, cross-sectional associations of all inflammatory markers (and CRP) and depression were significantly stronger in males compared to females. Longitudinally, only higher CRP was associated with subsequent higher depression in males, whereas only higher IL-6 was associated with subsequent higher depression in females, with depression not predicting inflammation in either sex. Thus, sex differences in the relationship between inflammatory markers and depression exist, but appear specific to the strength of associations, type of inflammatory marker, and directionality of longitudinal associations.

Our analyses extend prior reviews by comprehensively examining both cross-sectional and longitudinal studies, applying minimal exclusion criteria and including both categorical and continuous measures of depression. We conducted both sex-stratified models and meta-regressions to examine sex differences. Finally, our approach integrated both unadjusted and adjusted effect sizes, while prior studies have not [28]. Our most notable sex differences exist between CRP and IL-6, which are separate important components of an innate inflammatory response and often closely related in that IL-6 is an upstream regulator inducing the production of CRP in the liver [43]. Thus, it may be that inflammation and depression are associated in both males and females with subtle differences. Moreover, sex differences we identified relied on sex/gender as reported in our included studies. Most included papers did not explicitly define sex and/or gender, reported sex/gender as binary, and few provided data to disentangle biological sex versus gender effects. Thus, the current study combined sex/gender into a single binary variable of female versus male. Our results thus cannot distinguish between sex versus gender, and we interpreted findings as “sex” differences. Together, this suggests that existing theories may need revision to incorporate the role of sex differences, as sex-specific mediators and moderators could be crucial in understanding the vulnerability to depression associated with inflammation. However, this future work will need to better distinguish effects of sex versus gender, to understand underlying mechanisms (e.g., hormonal, genetic, psychosocial). One recent systematic review of 10 papers found limited evidence to date on hormonal influences on associations between mood disorders and inflammation but called for more research in this area [44].

Our cross-sectional, sex-specific analyses identified positive associations between depression and inflammation in both females and males. Specifically, using data from 211,926 females and 206,007 males, depression was associated with higher levels of all inflammatory markers combined, separately in each sex. This depression-inflammation association was significantly stronger among males compared to females. Regarding specific markers, depression was significantly associated with higher IL-6 in both females and males. However, depression was associated with higher CRP only among males. While other tested markers (i.e., TNF-α, IL-1β, fibrinogen) were not significantly associated with depression, all pooled estimates were in the positive direction. We present the first sex-stratified longitudinal meta-analyses of depression and subsequent inflammation across 10 studies including 17,240 participants (72% female) and inflammation and subsequent depression across 15 studies including 39,626 participants (68% female). Previous large-scale meta-analyses indicate significant bidirectional associations between depression and inflammatory markers over time when combining data across sex [18]. Our analyses identified that all inflammatory markers combined and IL-6 predict subsequent depression in females, and only CRP predicts subsequent depression in males. However, depression did not predict inflammation for females or males. This may be due to the relatively small sample size in our sex-stratified analyses compared to recent reviews that did not look at associations by sex or other methodological differences between the reviews [24]. Despite the differences observed in the present sex-stratified analyses, none of the longitudinal associations significantly differed by sex in meta-regressions. As longitudinal analyses relied on relatively fewer effects and studies, it is possible we were underpowered to detect sex moderation in meta-regressions. However, given lack of significant meta-regression differences, we caution interpretation of differential longitudinal findings by sex and emphasize that additional sex-specific longitudinal work is needed.

Our primary inflammatory measure was a combination of all inflammatory markers, providing a non-specific index of inflammatory activity that has less error variance than any individual marker [37]. Given that inflammation is a complex, multi-pathway process, including various markers together may more accurately capture the multidimensional immune response. However, markers may index different immune processes, and models incorporating various markers could be driven by the most common markers. Indeed, our results indicated that depression may have stronger associations with specific inflammatory markers in males versus females, underscoring the importance of specificity at the level of marker. Interestingly, there were no significant sex-stratified associations between depression and the individual markers IL-1β, TNF-α, and fibrinogen in either sex, though all pooled effect estimates were positive. Our significant results were found among CRP and IL-6, which were also the markers with the largest sample sizes and therefore highest power. It is critical that we continue to consider the complex and multifaceted nature of inflammation in psychoneuroimmunology by including multiple markers and using reliable and validated assays.

The comprehensive nature of the present review allowed for examination by level of confounding adjustment, study design, medical illness, and depression measure. In our unadjusted models, we saw consistent depression-inflammation associations across females and males. When fully adjusted effect sizes were considered, more clear sex differences emerged. In particular, depression-CRP associations remained significant for males only. This indicates that associations between depression and CRP among females may be sensitive to potential confounders. Notably, however, some covariates included in adjusted models, such as BMI, sleep, and physical activity, could be mediating pathway variables and adjusted estimates may be overly conservative. In other words, it is possible that elevated inflammation from any cause (e.g., psychological stress, environmental exposures) impairs sleep, which in turn drives the development of depressive symptoms. Unfortunately, many papers did not report multiple levels of adjustment or varied in what they covaried and as such, we were limited in our ability to shed light on the effects of different types of covariates. Given this, our primary results should be interpreted as potentially conservative, given overadjustment in some estimates for mediating variables. It is likely that true effect sizes fall between unadjusted and fully adjusted effect sizes (e.g., cross-sectional depression-all markers among males true effect between 0.05 and 0.31). For progress in the field, it is critical that researchers consider potentially mediating and confounding factors separately, and report multiple models with different levels of adjustment.

In secondary analyses, we compared depression-inflammation associations between samples with and without medical illness, and between samples with and without clinical depression measures. In females, effect sizes for depression-inflammation associations were similar among those with versus without medical illnesses. In contrast, in males, relationships between depression and inflammatory markers were generally stronger in samples without medical illness. In cross-sectional models for both sexes, estimates for depression-CRP associations were stronger in samples using clinical depression diagnoses than in those relying on continuous measures or non-clinical cutoffs. In contrast, a smaller recent meta-analysis reported significant associations of clinical diagnoses of depression with inflammatory markers in females only [28]. No significant depression-IL-6 associations were found when restricting to clinical depression diagnoses, though positive and significant associations were identified in both females and males when considering non-clinical depression measures, suggesting IL-6 may be linked with subclinical depressive symptoms. Of note, however, clinical depression subanalyses may have been underpowered.

Overall, estimates were small in magnitude, which could reflect our inclusion of the most adjusted estimates available (i.e., adjusting for confounders as well as potential mechanisms of elevated inflammation in depression), which are conservative estimates because some papers adjusted for potential mechanisms (e.g., BMI, sleep, physical activity) as well as potential confounders (e.g., age, race, socio-economic position). Our main estimates were similar in magnitude to some prior meta-analyses of depression and inflammation [24, 25], while smaller than others [23, 26, 27]. Some factors that could contribute to small effect sizes include the following. We used relatively broad inclusion criteria, which may have increased heterogeneity among the included studies and biased estimates towards the null. Against this possibility, our sensitivity analyses that stratified by some major potential sources of heterogeneity (e.g., medical illness samples, clinical depression versus not) revealed largely similar magnitude of effects overall. It is possible that there are other sources of heterogeneity that we were not able to address, which could be influencing the overall estimates and potentially biasing overall estimates towards the null. For example, there may be subgroups of individuals with depression while others do not show inflammatory profiles [9, 45, 46]. Indeed, despite identifying differences by sex in some cases, levels of heterogeneity across studies were high in our analyses. It is possible that differences in study samples, depression measures, assay type, covariates adjusted, and timing contributed to this heterogeneity and deserve further study. Another possibility is that our sex-stratified results are less confounded by publication bias than those included in prior meta-analyses. Sex differences were often not the goal of included studies, with many effect sizes gleaned from correlation matrices and supplementary analyses. Given known concerns about publication bias and the file drawer problem, it is possible that our inclusion of these effect sizes provide a more unbiased picture of depression-inflammation associations. It is also possible that the true associations between depression and inflammation by sex are simply relatively small in magnitude. Given our relatively large sample size, particularly in cross-sectional models, we had high power to detect small effects. However, even these small effects could be meaningful for theory and clinical practice. Shifts in average symptoms or in levels of inflammation can translate to population wide increases or decreases in health risk or diagnostic thresholds [47].

The included studies varied in methodology. For example, the use of different assays across studies introduces variability. Two thirds of studies failed to report or reported poor assay reliability, and there were not enough studies to examine moderation by assay type or blood fraction. This is a significant issue, especially considering that some commonly used assays have poor reliability [48–50]. Additionally, the methods used for assessing depression varied, with some studies using gold-standard clinical interviews for diagnoses in clinical samples and others using self-report measures in community samples. The quality of included studies varied, with lower quality studies potentially introducing greater risk of bias due to smaller sample size, lack of adjustment for confounders, inadequately addressing missingness, or unreliable assays. While studies overall had moderate quality, the quality of studies with regard to sex-specific analyses was 16% lower on average, which may indicate greater risk of bias in these studies. However, the lower quality scores primarily reflect that the sex-stratified analyses were not primary objectives in many studies, as evidenced by gaps in stated aims, study design, and confounding control. As mentioned previously, this lack of focus on sex differences in most papers is also a strength of this meta-analysis because bias was reduced. The majority of studies did not adequately adjust for factors that could impact depression-inflammation associations, such as sociodemographic factors and physical comorbidities. In contrast, many studies reported adjusted models that covaried potential mediating variables, which may obscure or reduce associations. Additional preregistered and longitudinal studies that clearly report on methodology, such as missing data, assay reliability and full statistical models in Supplementary materials are needed.

### Limitations

There are limitations to this meta-analysis. First, the majority of studies were cross-sectional, limiting our ability to fully examine longitudinal associations. Second, we were also unable to include all relevant articles due to missing necessary data to calculate standardized effect sizes. Third, we included a broad definition of depression, in line with several other meta-analyses [24–26]. However, this means that there are a range of measures of depression. While the vast majority are validated measures of depression, some category definitions were arbitrary or varied. Collapsing across continuous and diagnostic categories, for both depression and inflammation, was necessary to convert effect sizes but may obscure relevant distinctions. Finally, most studies examining depression-inflammation links failed to report sex-specific analyses, thus our sample is limited to a subset of the literature. Conversely, the present meta-analysis may have been less subject to publication bias because many papers did not have a primary focus on sex-stratified effects. Our sensitivity analyses suggest minimal publication bias, especially in cross-sectional studies, likely due to this fact and our proactive efforts in contacting authors. Some biomarkers (e.g., IL-10, interferon-γ) were assessed in few eligible studies, restricting our analyses to a subset including only the most common inflammatory markers examined in the depression literature. Additional research is needed examining these markers, some of which have been examined with depression in sex-stratified samples [51, 52].

### Agenda for future research

Rigorous longitudinal studies are needed to understand the directionality of associations between depression and inflammation by sex. Ideally, studies should include multiple follow-ups in situations where inflammatory activity changes to better understand the dynamics between inflammation and depressive symptom onset and progression. For instance, assessing depression and inflammation at baseline in a group who are likely to experience flares in inflammatory activity (e.g., people with inflammatory disorders), incorporating socio-demographic and health-related confounders, followed by multiple time points to observe mutual changes in these variables. This approach will help determine whether inflammation is a causal factor, a correlate, and/or in a bidirectional relationship with depression. Moreover, rigorous longitudinal studies could provide insights into whether inflammation could serve as a biomarker for specific depression subtypes [46]. Mechanisms involved in the onset of depression may not necessarily be associated with clinical progression [53], so longitudinal studies could also help inform treatment. Additionally, only some included studies examined depression symptom clusters separately (e.g., cognitive and somatic symptoms); greater focus on depression dimensionality could reveal differential associations with inflammatory markers [54, 55]. Similarly, some depressive phenotypes may be particularly associated with elevated inflammation and more amenable to anti-inflammatory treatment [46]. Elevated baseline inflammation predicts worse response to selective serotonin reuptake inhibitors (SSRIs) and females tend to show better responses to SSRIs than males, raising the possibility that inflammation may play a role in treatment resistance in males [56]. In addition to research on directionality, additional work is needed to understand if associations between depression and inflammation are clinically meaningful and should inform clinical practice. Some research suggests only a subset of individuals with depression will demonstrate elevated inflammatory phenotypes, potentially due to earlier exposure to adversity or differential genetic risk [46, 57, 58]. Future research that focuses on characterizing these subsets, including whether they differ by sex, is critical.

## Conclusions

This comprehensive systematic review and meta-analysis provides evidence of the potential cross-sex relevance of inflammation in depression, but also for sex differences in associations between depression and specific inflammatory markers, both cross-sectionally and longitudinally. These studies suggest a need to incorporate sex into future studies and to include hypotheses regarding stronger associations in males in our conceptual models and theories. While more research is needed, these findings have relevance for future screening and intervention efforts that incorporate inflammatory markers. High-quality, longitudinal studies are needed to serve as the foundation for future theoretical and research advancements in this field.

## Supplementary information

Supplement
