# Supplementary material for: Sex-specific associations of depression with inflammatory markers: a systematic review and meta-analysis
Source: Transl Psychiatry. 2026 Jun 2;16:394. doi: 10.1038/s41398-026-04139-7 (PMC13443555; doi:10.1038/s41398-026-04139-7)
Supplement: Supplementary file 1 [file 41398_2026_4139_MOESM1_ESM.docx]

**Supplement**

**Sex-specific associations of depression with inflammatory markers: A systematic review and meta-analysis**

Szabo, Nishimi, *et al.*

**TABLE OF CONTENTS**

[Table S1: Preferred Reporting Items for Systematic Reviews and Meta-Analyses (PRISMA)	5](#_Toc228212893)

[Detailed Methods	8](#_Toc228212894)

[Appendix S1: Inclusion and exclusion criteria and determination	8](#_Toc228212895)

[Appendix S2: Data extraction and processing	9](#_Toc228212896)

[Appendix S3: Full search string	11](#_Toc228212897)

[Appendix S4: Changes and deviations from preregistered protocol	12](#_Toc228212898)

[Appendix S5: Statistical analysis	14](#_Toc228212899)

[Appendix S6: Study quality and risk of bias assessment	15](#_Toc228212900)

[Table S2: Quality criteria adapted from MINOR guidelines	16](#_Toc228212901)

[Appendix S7: Sensitivity analyses for evaluating confounding control	18](#_Toc228212902)

[Detailed Results	19](#_Toc228212903)

[Table S3. Characteristics of Included Studies	19](#_Toc228212904)

[Table S4: Quality of included studies	25](#_Toc228212905)

[List of Included Papers	31](#_Toc228212906)

[Table S5-S19: Sensitivity Analyses: Meta-analytic and meta-regression results	42](#_Toc228212907)

[Table S5. Sensitivity analyses: meta-analytic results for cross-sectional studies: depression-biomarker associations separately among females and males	42](#_Toc228212908)

[Table S6. Sensitivity analyses: meta-analytic results for longitudinal studies: depression-biomarker associations separately among females and males	43](#_Toc228212909)

[Table S7. Meta-regression results for effects of level of confounding adjustment in cross-sectional studies: depression-biomarker associations	44](#_Toc228212910)

[Table S8. Meta-regression results for effects of level of confounding adjustment in longitudinal studies: depression-biomarker associations	46](#_Toc228212911)

[Table S9. Meta-analytic results for cross-sectional studies: depression-biomarker associations separately among females and males, restricted to unadjusted estimates	48](#_Toc228212912)

[Table S10. Meta-analytic results for longitudinal studies: depression-biomarker associations separately among females and males, restricted to unadjusted estimates	49](#_Toc228212913)

[Table S11. Meta-analytic results for cross-sectional studies: depression-biomarker associations separately among females and males, restricted to fully adjusted estimates	50](#_Toc228212914)

[Table S12. Meta-analytic results for longitudinal studies: depression-biomarker associations separately among females and males, restricted to fully adjusted estimates	51](#_Toc228212915)

[Table S13. Meta-analytic results for cross-sectional studies: depression-biomarker associations separately among females and males, restricted to medical illness samples	52](#_Toc228212916)

[Table S14. Meta-analytic results for cross-sectional studies: depression-biomarker associations separately among females and males, restricted to non-medical illness samples	53](#_Toc228212917)

[Table S15. Meta-analytic results for longitudinal studies: depression-biomarker associations separately among females and males, restricted to non-medical illness samples	54](#_Toc228212918)

[Table S16. Meta-analytic results for cross-sectional studies: depression-biomarker associations separately among females and males, restricted to samples assessing clinical depression diagnosis	55](#_Toc228212919)

[Table S17. Meta-analytic results for longitudinal studies: depression-biomarker associations separately among females and males, restricted to samples assessing clinical depression diagnosis	56](#_Toc228212920)

[Table S18. Meta-analytic results for cross-sectional studies: depression-biomarker associations separately among females and males, restricted to samples NOT assessing clinical depression diagnoses	57](#_Toc228212921)

[Table S19. Meta-analytic results for longitudinal studies: depression-biomarker associations separately among females and males, restricted to samples NOT assessing clinical depression diagnoses	58](#_Toc228212922)

[Figure S1-S22: Forest plots	59](#_Toc228212923)

[Figure S1: Forest plot of depression and all markers combined among females in cross-sectional studies (k_papers_=85; k_estimates_=147; n_observations_=211,926)	60](#_Toc228212924)

[Figure S2: Forest plot of depression and IL-6 among females in cross-sectional studies (k_papers_=40; k_estimates_=41; n_observations_=11,565)	61](#_Toc228212925)

[Figure S3: Forest plot of depression and TNF-α among females in cross-sectional studies (k_papers_=22; k_estimates_=23; n_observations_=5,550)	62](#_Toc228212926)

[Figure S4: Forest plot of depression and IL-1β among females in cross-sectional studies (k_papers_=11; k_estimates_=11; n_observations_=2,708)	63](#_Toc228212927)

[Figure S5: Forest plot of depression and fibrinogen among females in cross-sectional studies (k_papers_=3; k_estimates_=3; n_observations_=1,238)	64](#_Toc228212928)

[Figure S6: Forest plot of depression and all markers combined among males in cross-sectional studies (k_papers_=71; k_estimates_=108; n_observations_=206,007)	65](#_Toc228212929)

[Figure S7: Forest plot of depression and IL-6 among males in cross-sectional studies (k_papers_=27; k_estimates_=27; n_observations_=18,272)	66](#_Toc228212930)

[Figure S8: Forest plot of depression and TNF-α among males in cross-sectional studies (k_papers_=11; k_estimates_=11; n_observations_=6,083)	67](#_Toc228212931)

[Figure S9: Forest plot of depression and IL-1β among males in cross-sectional studies (k_papers_=6; k_estimates_=6; n_observations_=1,905)	68](#_Toc228212932)

[Figure S10: Forest plot of depression and fibrinogen among males in cross-sectional studies (k_papers_=6; k_estimates_=6; n_observations_=3,272)	69](#_Toc228212933)

[Figure S11: Forest plot of depression and all markers combined among females in longitudinal studies – depression and subsequent inflammation (k_papers_=6; k_estimates_=9; n_observations_=14,083)	70](#_Toc228212934)

[Figure S12: Forest plot of depression and CRP among females in longitudinal studies – depression and subsequent inflammation (k_papers_=6; k_estimates_=6; n_observations_=12,492)	71](#_Toc228212935)

[Figure S13: Forest plot of depression and IL-6 among females in longitudinal studies – depression and subsequent inflammation (k_papers_=2; k_estimates_=2; n_observations_=1,388)	72](#_Toc228212936)

[Figure S14: Forest plot of depression and all markers combined among males in longitudinal studies – depression and subsequent inflammation (k_papers_=7; k_estimates_=15; n_observations_=10,602)	73](#_Toc228212937)

[Figure S15: Forest plot of depression and CRP among males in longitudinal studies – depression and subsequent inflammation (k_papers_=7; k_estimates_=7; n_observations_=5,006)	74](#_Toc228212938)

[Figure S16: Forest plot of depression and IL-6 among males in longitudinal studies – depression and subsequent inflammation (k_papers_=5; k_estimates_=5; n_observations_=4,022)	75](#_Toc228212939)

[Figure S17: Forest plot of depression and all markers combined among females in longitudinal studies – inflammation and subsequent depression (k_papers_=12; k_estimates_=17; n_observations_=29,624)	76](#_Toc228212940)

[Figure S18: Forest plot of depression and CRP among females in longitudinal studies – inflammation and subsequent depression (k_papers_=11; k_estimates_=11; n_observations_=24,702)	77](#_Toc228212941)

[Figure S19: Forest plot of depression and IL-6 among females in longitudinal studies – inflammation and subsequent depression (k_papers_=5; k_estimates_=5; n_observations_=4,824)	78](#_Toc228212942)

[Figure S20: Forest plot of depression and all markers combined among males in longitudinal studies – inflammation and subsequent depression (k_papers_=11; k_estimates_=18; n_observations_=19,830)	79](#_Toc228212943)

[Figure S21: Forest plot of depression and CRP among males in longitudinal studies – inflammation and subsequent depression (k_papers_=10; k_estimates_=10; n_observations_=14,043)	80](#_Toc228212944)

[Figure S22: Forest plot of depression and IL-6 among males in longitudinal studies – inflammation and subsequent depression (k_papers_=6; k_estimates_=6; n_observations_=4,568)	81](#_Toc228212945)

[Figure S23-S46: Significance Funnel Plots	82](#_Toc228212946)

[Figure S23: Significance funnel plot of depression and all markers combined among females in cross-sectional studies	82](#_Toc228212947)

[Figure S24: Significance funnel plot of depression and CRP among females in cross-sectional studies	83](#_Toc228212948)

[Figure S25: Significance funnel plot of depression and IL-6 among females in cross-sectional studies	84](#_Toc228212949)

[Figure S26: Significance funnel plot of depression and TNF-α among females in cross-sectional studies	85](#_Toc228212950)

[Figure S27: Significance funnel plot of depression and IL-1β among females in cross-sectional studies	86](#_Toc228212951)

[Figure S28: Significance funnel plot of depression and fibrinogen among females in cross-sectional studies	87](#_Toc228212952)

[Figure S29: Significance funnel plot of depression and all markers combined among males in cross-sectional studies	88](#_Toc228212953)

[Figure S30: Significance funnel plot of depression and CRP among males in cross-sectional studies	89](#_Toc228212954)

[Figure S31: Significance funnel plot of depression and IL-6 among males in cross-sectional studies	90](#_Toc228212955)

[Figure S32: Significance funnel plot of depression and TNF-α among males in cross-sectional studies	91](#_Toc228212956)

[Figure S33: Significance funnel plot of depression and IL-1β among males in cross-sectional studies	92](#_Toc228212957)

[Figure S34: Significance funnel plot of depression and fibrinogen among males in cross-sectional studies	93](#_Toc228212958)

[Figure S35: Significance funnel plot of depression and all markers combined among females in longitudinal studies – depression and subsequent inflammation	94](#_Toc228212959)

[Figure S36: Significance funnel plot of depression and CRP among females in longitudinal studies – depression and subsequent inflammation	95](#_Toc228212960)

[Figure S37: Significance funnel plot of depression and IL-6 among females in longitudinal studies – depression and subsequent inflammation	96](#_Toc228212961)

[Figure S38: Significance funnel plot of depression and all biomarkers combined among males in longitudinal studies – depression and subsequent inflammation	97](#_Toc228212962)

[Figure S39: Significance funnel plot of depression and CRP among males in longitudinal studies – depression and subsequent inflammation	98](#_Toc228212963)

[Figure S40: Significance funnel plot of depression and IL-6 among males in longitudinal studies – depression and subsequent inflammation	99](#_Toc228212964)

[Figure S41: Significance funnel plot of depression and all biomarkers combined among females in longitudinal studies – inflammation and subsequent depression	100](#_Toc228212965)

[Figure S42: Significance funnel plot of depression and CRP among females in longitudinal studies – inflammation and subsequent depression	101](#_Toc228212966)

[Figure S43: Significance funnel plot of depression and IL-6 among females in longitudinal studies – inflammation and subsequent depression	102](#_Toc228212967)

[Figure S44: Significance funnel plot of depression and all biomarkers combined among males in longitudinal studies – inflammation and subsequent depression	103](#_Toc228212968)

[Figure S45: Significance funnel plot of depression and CRP among males in longitudinal studies – inflammation and subsequent depression	104](#_Toc228212969)

[Figure S46: Significance funnel plot of depression and IL-6 among males in longitudinal studies – inflammation and subsequent depression	105](#_Toc228212970)

[References	106](#_Toc228212971)

## Table S1: Preferred Reporting Items for Systematic Reviews and Meta-Analyses (PRISMA)

| **Section and Topic** | **Item #** | **Checklist item** | **Location where item is reported** |
| --- | --- | --- | --- |
| **TITLE** | | |  |
| Title | 1 | Identify the report as a systematic review. | Page 1 |
| **ABSTRACT** | | |  |
| Abstract | 2 | See the PRISMA 2020 for Abstracts checklist. | Page 2 |
| **INTRODUCTION** | | |  |
| Rationale | 3 | Describe the rationale for the review in the context of existing knowledge. | Page 3-4 |
| Objectives | 4 | Provide an explicit statement of the objective(s) or question(s) the review addresses. | Page 4 |
| **METHODS** | | |  |
| Eligibility criteria | 5 | Specify the inclusion and exclusion criteria for the review and how studies were grouped for the syntheses. | Page 5, Appendix S1 |
| Information sources | 6 | Specify all databases, registers, websites, organisations, reference lists and other sources searched or consulted to identify studies. Specify the date when each source was last searched or consulted. | Page 5, Appendix S3 |
| Search strategy | 7 | Present the full search strategies for all databases, registers and websites, including any filters and limits used. | Page 5, Appendix S3 |
| Selection process | 8 | Specify the methods used to decide whether a study met the inclusion criteria of the review, including how many reviewers screened each record and each report retrieved, whether they worked independently, and if applicable, details of automation tools used in the process. | Page 5,  Appendix S1 |
| Data collection process | 9 | Specify the methods used to collect data from reports, including how many reviewers collected data from each report, whether they worked independently, any processes for obtaining or confirming data from study investigators, and if applicable, details of automation tools used in the process. | Page 5,  Appendix S2 |
| Data items | 10a | List and define all outcomes for which data were sought. Specify whether all results that were compatible with each outcome domain in each study were sought (e.g. for all measures, time points, analyses), and if not, the methods used to decide which results to collect. | Page 5, Appendix S2 |
|  | 10b | List and define all other variables for which data were sought (e.g. participant and intervention characteristics, funding sources). Describe any assumptions made about any missing or unclear information. | Appendix S2 |
| Study risk of bias assessment | 11 | Specify the methods used to assess risk of bias in the included studies, including details of the tool(s) used, how many reviewers assessed each study and whether they worked independently, and if applicable, details of automation tools used in the process. | Page 6, Appendix S6, Table S2 |
| Effect measures | 12 | Specify for each outcome the effect measure(s) (e.g. risk ratio, mean difference) used in the synthesis or presentation of results. | Page 5, Appendix S2 |
| Synthesis methods | 13a | Describe the processes used to decide which studies were eligible for each synthesis (e.g. tabulating the study intervention characteristics and comparing against the planned groups for each synthesis (item #5)). | Page 5, Appendix S1 |
|  | 13b | Describe any methods required to prepare the data for presentation or synthesis, such as handling of missing summary statistics, or data conversions. | Page 5, Appendix S2 |
|  | 13c | Describe any methods used to tabulate or visually display results of individual studies and syntheses. | Page 5 |
|  | 13d | Describe any methods used to synthesize results and provide a rationale for the choice(s). If meta-analysis was performed, describe the model(s), method(s) to identify the presence and extent of statistical heterogeneity, and software package(s) used. | Page 5, Appendix S5 |
|  | 13e | Describe any methods used to explore possible causes of heterogeneity among study results (e.g. subgroup analysis, meta-regression). | Page 6, Appendix S5, S7 |
|  | 13f | Describe any sensitivity analyses conducted to assess robustness of the synthesized results. | Page 7, Appendix S5, S7 |
| Reporting bias assessment | 14 | Describe any methods used to assess risk of bias due to missing results in a synthesis (arising from reporting biases). | Page 6, Appendix S5, S6 |
| Certainty assessment | 15 | Describe any methods used to assess certainty (or confidence) in the body of evidence for an outcome. | Page 6, Appendix S5, S7 |
| **RESULTS** | | |  |
| Study selection | 16a | Describe the results of the search and selection process, from the number of records identified in the search to the number of studies included in the review, ideally using a flow diagram. | Page 7, Figure 1 |
|  | 16b | Cite studies that might appear to meet the inclusion criteria, but which were excluded, and explain why they were excluded. | Page 7 |
| Study characteristics | 17 | Cite each included study and present its characteristics. | Page 7, Table S3 |
| Risk of bias in studies | 18 | Present assessments of risk of bias for each included study. | Page 8, Table S4 |
| Results of individual studies | 19 | For all outcomes, present, for each study: (a) summary statistics for each group (where appropriate) and (b) an effect estimate and its precision (e.g. confidence/credible interval), ideally using structured tables or plots. | Tables 1, 2, Figures 1, S2-S23 |
| Results of syntheses | 20a | For each synthesis, briefly summarise the characteristics and risk of bias among contributing studies. | Page 8-10 |
|  | 20b | Present results of all statistical syntheses conducted. If meta-analysis was done, present for each the summary estimate and its precision (e.g. confidence/credible interval) and measures of statistical heterogeneity. If comparing groups, describe the direction of the effect. | Page 8-10, Tables 1, 2, Figures 2, S2-S23 |
|  | 20c | Present results of all investigations of possible causes of heterogeneity among study results. | Tables S5-19, Figures S24-S47 |
|  | 20d | Present results of all sensitivity analyses conducted to assess the robustness of the synthesized results. | Tables S5-19, Figures S24-S47 |
| Reporting biases | 21 | Present assessments of risk of bias due to missing results (arising from reporting biases) for each synthesis assessed. | Page 10, Tables S5-S6, Figures S24-S47 |
| Certainty of evidence | 22 | Present assessments of certainty (or confidence) in the body of evidence for each outcome assessed. | Page 8-10, Table S5-S23 |
| **DISCUSSION** | | |  |
| Discussion | 23a | Provide a general interpretation of the results in the context of other evidence. | Page 10-13 |
|  | 23b | Discuss any limitations of the evidence included in the review. | Page 13-14 |
|  | 23c | Discuss any limitations of the review processes used. | Page 13-14 |
|  | 23d | Discuss implications of the results for practice, policy, and future research. | Page 14-15 |
| **OTHER INFORMATION** | | |  |
| Registration and protocol | 24a | Provide registration information for the review, including register name and registration number, or state that the review was not registered. | Page 4 |
|  | 24b | Indicate where the review protocol can be accessed, or state that a protocol was not prepared. | Page 4,  Appendix S4 |
|  | 24c | Describe and explain any amendments to information provided at registration or in the protocol. | Page 4,  Appendix S4 |
| Support | 25 | Describe sources of financial or non-financial support for the review, and the role of the funders or sponsors in the review. | Page 17 |
| Competing interests | 26 | Declare any competing interests of review authors. | Page 17 |
| Availability of data, code and other materials | 27 | Report which of the following are publicly available and where they can be found: template data collection forms; data extracted from included studies; data used for all analyses; analytic code; any other materials used in the review. | Appendix S5 |

*From:* Page MJ, McKenzie JE, Bossuyt PM, Boutron I, Hoffmann TC, Mulrow CD, et al. The PRISMA 2020 statement: an updated guideline for reporting systematic reviews. BMJ 2021;372:n71. doi: 10.1136/bmj.n71

## Detailed Methods

### Appendix S1: Inclusion and exclusion criteria and determination

**Full inclusion criteria:**

1. The study measured at least one of the following inflammatory markers in plasma or serum: C-reactive protein (CRP), fibrinogen, tumor necrosis factor- α (TNF-α), and any interleukin.
2. The study included a measure or diagnosis of depressive or anxiety symptoms.
3. Subjects were adults aged over 18 years old.
4. Paper was published in a peer-reviewed journal.
5. The paper was written in the English language.

**Exclusion Criteria:**

1. The study specifically recruited cancer patients.
2. The study specifically recruited pregnant women.
3. Inflammation was measured via stimulated cells, blood spot or cerebrospinal fluid.
4. The study specifically recruited subject based on used electroconvulsive treatment or psychotropic medication treatment for depression/anxiety, or the study assigned on of these treatment modalities (unless the association between depression/anxiety and inflammation was reported at baseline).
5. The study population was specifically recruited for having an acute infection or injury within the past year.
6. The study experimentally manipulated depression, anxiety or inflammation (unless a baseline correlation was reported).

### Appendix S2: Data extraction and processing

For each paper, reviewers (YS, KN, AS, MS, AN) recorded all statistics needed to compute or estimate the Cohen’s d corresponding to the standardized mean difference between a binary exposure (e.g., having versus not having depression, higher versus lower inflammation) and outcome (e.g., continuous inflammation level, continuous depressive symptom levels) in terms of standard deviations of the outcome. Our sensitivity analyses considered the level of adjustment for potential confounders. For papers that reported multiple models with several levels of adjustment, we included estimates from the unadjusted, minimally adjusted, and most adjusted model reported. All data extracted was confirmed by another author. When there was any uncertainty about data extraction, issues were reviewed in a consensus meeting involving at least three authors (YS, KN, AS, AN, MS, and AOD).

Several conversions and decisions were made to retain all possible papers in analyses and ensure effect estimates were on the same scale. For papers that reported statistics on scales other than mean differences, such as unstandardized regression estimates or risk ratios, we approximately converted them to the Cohen’s *d* scale.^1–4^ Specifically, we calculated or approximated effect sizes using combinations of the following statistics depending on availability: means, standard deviations, standard errors, sample size, Pearson’s *r*, *R²*, coefficients from linear regression models, odds ratios, risk ratios, *t*-values, *p*-values, and 95% confidence intervals. When necessary information was not available, we emailed the corresponding author to attempt to obtain the needed statistics. When the sample size was unclear for the sample size of reported analyses, we assumed the smallest size reported in the paper. For papers that needed ancillary information not presented in the paper (i.e., M/SD of the predictor or outcome), we attempted to use information from other papers using the same study sample with the same independent and dependent variables^5^ but at times used other papers from the same study sample that focused on other measures or papers that were excluded from our analyses but used the same independent and dependent variables. For papers that reported necessary M/SD information by subcategories (i.e., levels of inflammatory marker by tertile of depression), we calculated an average using a weighted mean and SD.^6^ For papers that provided necessary M/SD information or we imputed from another paper, but used log-transformed values in analyses, we approximated the log transformed values using Taylor approximation.^7^ When multiple estimates were reported within a single paper, we included standardized over unstandardized effect estimate and included the estimate with the largest sample size, unless the necessary information was not provided and the authors did not respond to requests for information, in which case estimates with available necessary information was used. When possible, we retained both papers by using the largest adjusted analysis in the main article and including both papers in follow-up analyses examining level of confounding control. When studies did not report needed statistics, but included figures that indicated the relevant information, the values were approximated using R package “digitize” (k = 6). Notably, papers could contribute multiple estimates (e.g., females and males with CRP and IL-6; or stratified estimates such as CRP among pre-menopausal versus post-menopausal females), thus the number of papers was often smaller than the number of estimates for any given model.

### Appendix S3: Full search string

**Search Terms and Settings**

Depression-related terms included “depression”, or “depressive”, or “depressed”. Immune-related terms included: “inflammation”, or “inflammatory”, or “C-reactive protein”, or “CRP”, or “interleukin”, or “cytokine”, or “interferon”, or “tumor necrosis factor”, or “TNF”, or “transforming growth factor”, or “TGF”, or “acute phase protein”. This project also examined anxiety related outcomes and included additional keywords of “anxiety” or “anxious” that are not used in this paper.

The keywords had to appear in articles’ titles or abstracts, and we restricted for the inclusion of human adults only and papers written in the English language. We did not use the human and adult filters for papers published within the past year as indexing of these filters are delayed after papers first appear in PubMed.

**Search Methods**

PubMed, PsycINFO, and Scopus were searched in May 2018, January 2019, and January 2023 for papers examining the relationship between depression and inflammation, beginning at database inception (no start date specification). We also utilized reference treeing, reviewing references from related meta-analyses and eligible papers to identify additional papers to screen.

### Appendix S4: Changes and deviations from preregistered protocol

**Change in preregistration from V1 to V2**

Our first (V1) preregistration was submitted on January 30^th^, 2019. At this point, an initial database search was completed, and article review and data extraction were ongoing. However, no data analysis had been conducted. We updated our preregistration and uploaded it on July 23^rd^, 2020, at which point article review and data extract was ongoing, but no data analysis had been conducted. Some of the code to derive effect sizes was written.

- In the V2 preregistration, we updated the previous version to state that we will analyze data for markers including: IL-6, CRP, TNF-α, IL-1β, and others.
- We also updated the data extraction section to reflect that we will be collecting data relevant to calculating Hedges’ *g* rather than Cohen’s *d* (V1 preregistration) because Hedges’ *g* has a more robust variance estimation that does not require distributional assumptions and that can accommodate clustering of estimates within papers.^8,9^
- The V2 preregistration included updated information on our statistical analysis with more detail on what will be reported.

**Change from V2 preregistration to current paper**

- When we updated the preregistration prior to analyses, we proposed to move from Hedges’ *g* to Cohen’s *d* because Hedges’ *g* has a more robust variance estimation that does not require distributional assumptions and that can accommodate clustering of estimates within papers. However, the conversions to derive Hedges’ *g* from available information created implausible values. Therefore, we reverted to Cohen’s *d*, which we had originally preregistered (V1).
- Because of the length of time working on this project (i.e., due to delays caused by the COVID-19 pandemic), we repeated our search three times. Each time, more eligible papers were identified. Therefore, we had sufficient papers to include fibrinogen, which was not named in the preregistration. We have also listed authors by initials in the preregistration. As this project extended over time, additional authors were included. We did not analyze “other” due to too few studies for each additional biomarker.
- We included analyses across all included biomarkers. This was added post hoc but reflects a change in the field to consider inflammatory responses more broadly. Analyses using an inflammatory factor, a composite measure of multiple cytokines/acute phase proteins, have become more prevalent in recent years.^10–12^
- Preregistration says “decisions of each reviewer will be reviewed by another reviewer” in inclusions and “we will double code a subset” in data extraction. For both, a subset was double coded (i.e., independently), but all exclusions and all data extracted was confirmed by another reviewer.
- Preregistration notes planned sensitivity analyses for publication bias, but does directly not mention plots. We include significance plots to help visualize publication bias.
- In additional sensitivity analyses for confounding, we ran the primary cross-sectional and longitudinal models restricted to only unadjusted estimates and to only fully adjusted estimates.
- We included additional sensitivity analyses that stratified by sample type: medical illness samples vs not, and clinical diagnoses of depression vs not (e.g., self-report symptom scales).
- We noted that our preregistration was broad in our description of the literature search and provided additional information on filters. Specifically, settings used that were not explicitly noted were 1) the checking of human and adult in the advanced search options for PubMed and PsycINFO and 2) the exclusion of articles indexed in Medline while conducting Scopus. The former was chosen to be consistent with our pre-registered inclusion criteria. The latter allows us to remove potential duplicates with PubMed (i.e., papers indexed in Medline) and greatly reduces the number of results. This approach is consistent with other reviews that utilize Scopus.^13^

### Appendix S5: Statistical analysis

**Additional sensitivity analyses**

To assess sensitivity to unmeasured confounding, we computed E-values and 95% confidence intervals for the meta-analytic point estimates^17,18^ and the strength of unmeasured confounding needed to reduce the proportion of true causal effects stronger than a magnitude of Cohen’s *d*=0.2 to below 10%.^17^ To further examine how confounding, sample characteristics, and measurement impacted estimates, we fit meta-regressions with a categorical covariate for confounding adjustment (i.e., unadjusted, minimally adjusted, and fully adjusted); in these models, some studies contributed multiple estimates and RVE estimation method accounts for the resulting correlation of estimates within papers. We also conducted separate sex-stratified meta-analyses by 1) only unadjusted versus only fully adjusted estimates, 2) samples focused on medical illness populations versus not, and 3) clinical depression diagnoses versus not. In these additional post-hoc sensitivity analyses, we re-ran the primary meta-analyses stratified by estimates from samples selected for medical illness (e.g., cardiovascular disease, obesity) versus not selected for medical illness, and samples using clinical diagnoses of depression (e.g., DSM diagnoses or structured interview performed by a trained clinician) versus not (e.g., self-report symptom scales).

To assess sensitivity to publication bias, we fit Vevea & Hedges’s selection model with a cutpoint at *p*=0.05 to each primary meta-analysis comprising at least 30 estimates, with at least 5 affirmative (i.e., significant and with a positive point estimate) and 5 non-affirmative (i.e., nonsignificant or with a negative point estimate).^17,19^ To visualize potential publication bias, we plotted significance funnels for all primary sex-stratified meta-analyses.^17^ We additionally re-ran analyses restricting to only non-affirmative studies to provide estimates corrected for hypothetical “worst-case” scenarios of publication bias or forms of p-hacking.^17^ The latter method is an intentionally conservative sensitivity analysis.

Code and other materials used in the current study are available upon request to the corresponding author.

### Appendix S6: Study quality and risk of bias assessment

**Quality Assessment**

At the study level, two authors (YS, KN, AS, MS) assessed quality and risk of bias for each paper using a questionnaire containing 8 items adapted from the Methodological Index for Non-Randomized Studies (MINORS) criteria,^20^ each rated on a 0 to 2 scale; possible scores ranged from 0 to 16. Study-level quality was assessed in general (i.e., for the paper broadly) and for sex-specific analyses (i.e., with respect to sex-stratified or sex-specific analyses of depression-inflammation associations); see **Table S2** for a detailed description of quality criteria. For example, a paper may have been longitudinal or prospective in design but only examined sex-differences cross-sectionally at baseline or a paper may have reported results of an intervention and only the cross-sectional baseline data was eligible for inclusion. All quality ratings were confirmed by a second author; all discrepancies were resolved by discussion and a third author brought it when discussion did not resolve the discrepancy (YS, KN, MS, AS, AOD). A narrative summary of study quality and risk of bias was conducted.

### Table S2: Quality criteria adapted from MINOR guidelines

| **1) Stated Aim** | |
| --- | --- |
| **1A) Stated Aim: General** | Clearly stated aim: 0 = not reported 1 = reported but inadequate 2 = reported and adequate referring to the paper in general |
| **1B) Stated Aim: Sex-Specific** | Clearly stated aim:  0 = not reported 1 = reported but inadequate (e.g., no sex differences) 2 = reported and adequate (e.g., sex differences or moderators; or single sex study) referring to sex differences in depression and inflammation association specifically |
| **2) Inclusion Exclusion Criteria** | 0 = not reported 1 = reported but inadequate (e.g., only reports inclusion OR exclusion criteria, only refers to parent study) 2 = reported and adequate |
| **3) Pre-register** | |
| **3A) Pre-register: General** | Preregistered: 0 = not reported 1 = reported but inadequate (i.e., preregistered but lacking detail on either data collection or analysis plan)  2 = reported and adequate  referring to the paper in general |
| **3B) Pre-register: Sex-Specific** | Preregistered: 0 = not reported 1 = reported but inadequate (i.e., any preregistration) 2 = reported and adequate (i.e., preregistration of sex differences; or single sex study)  referring to sex differences in depression and inflammation association specifically |
| **4) Study Design** | |
| **4A) Study Design: General** | 0 = cross-sectional  1 = longitudinal with exposure clearly measured before outcome (with or without adjustment for confounders) 2 = longitudinal with exposure clearly measured before outcome AND outcome measured at baseline (with or without adjustment for confounders)  referring to the paper in general |
| **4B) Study Design: Sex-Specific** | 0 = cross-sectional  1 = longitudinal with exposure clearly measured before outcome (with or without adjustment for confounders) 2 = longitudinal with exposure clearly measured before outcome AND outcome measured at baseline (with or without adjustment for confounders)  referring to sex differences in depression and inflammation association specifically |
| **5) Missing Data** | |
| **5A) Missing Data: General** | 0 = not reported 1 = reported but inadequate (5-40% data missing and no statistically principled method used, more than 40% data missing regardless of method use) 2 = reported and adequate (less than 5% missing data, or 5-40% and principled statistical method [e.g., multiple imputation] used)  referring to the paper in general |
| **5B) Missing Data: Sex-Specific** | 0 = not reported 1 = reported but inadequate (5-40% data missing and no statistically principled method used, more than 40% data missing regardless of method use) 2 = reported and adequate (less than 5% missing data, or 5-40% and principled statistical method [e.g., multiple imputation] used)  referring to sex differences in depression and inflammation association specifically |
| **6) Assay Reliability** | intraassay < 10% and interassay < 15% (specific for biomarkers included in current analyses): 0 = not reported OR samples analyzed in singulate OR only interassay 1 = reported but inadequate (i.e., only intraassay reported OR outside of range [either inter or intraassay] OR reference the reliability of the study samples in another paper) 2 = reported and adequate (i.e., reported both inter and intraassay AND in range) based on lowest reliability of markers reported |
| **7) Confounds** | |
| **7A) Confounds: General** | 0 = not reported or no covariates 1 = reported but inadequate (controls for only ONE domain of confounds) 2 = reported and adequate (controls for both demographic AND health-related factors  referring to the paper in general |
| **7B) Confounds: Sex-Specific** | 0 = not reported or no covariates 1 = reported but inadequate (controls for only ONE domain of confounds) 2 = reported and adequate (controls for both demographic AND health-related factors  referring to sex differences in depression and inflammation association specifically |
| **8) Sample Size** | |
| **8A) Sample Size: General** | 0 = no a priori power analysis, powered for large effect only (n < 82) 1 = no a priori power analysis, powered for medium effect only (82 ≤ n < 779) 2 = a priori power analysis for prospective determination of sample size OR powered for small effect (n ≥ 779)  referring to the paper in general |
| **8B) Sample Size: Sex-Specific** | 0 = no a priori power analysis, powered for large effect only (n < 82) 1 = no a priori power analysis, powered for medium effect only (82 ≤ n < 779) 2 = a priori power analysis for prospective determination of sample size OR powered for small effect (n ≥ 779)  rating based on lowest sample size  referring to sex differences in depression and inflammation association specifically |

**Reference**: Slim, Karem et al. “Methodological index for non-randomized studies (minors): development and validation of a new instrument.” ANZ journal of surgery vol. 73,9 (2003): 712-6. doi:10.1046/j.1445-2197.2003.02748.x

### Appendix S7: Sensitivity analyses for evaluating confounding control

**Confounding Control**

We preregistered to conduct sensitivity analyses that examined effect sizes comparing cross-sectional papers that adjusted for all major suspected confounders (e.g., age, medical illness, body mass index) with cross-sectional studies that do not adjust for all the major suspected confounders. Given that papers often reported multiple adjusted models, this was expanded to include analyses that considered models that were 1) unadjusted, 2) minimally adjusted for socio-demographic factors, and 3) fully adjusted for health behaviors and/or disease. Unadjusted models were generally those with no covariates (i.e., bivariate associations between depression and inflammation), but if the paper stratified analyses by race only, we included models with no additional covariates as unadjusted. Minimally adjusted was defined as only adjusting for one or more demographic variable. Fully adjusted indicated adjustment for health behaviors and/or other disease related variables. For papers that reported several fully adjusted models (e.g., model 1 had demographics and health behaviors, model 2 had demographics and more health behaviors), we chose the most adjusted model as our fully adjusted model. Some papers contributed multiple effect estimates to our analyses, but only one per category (e.g., a paper could contribute an unadjusted and a minimally adjusted effect estimate from the same sample, but could not contribute two minimally adjusted effect estimates).

## Detailed Results

### Table S3. Characteristics of Included Studies

| **First Author, Year** | **Sample Size Male** | **Sample Size Female** | **Biomarker Assessed** | **Fluid** | **Depression Measure** | **Clinical Depression Diagnosis?** | **Quality Total: General** | **Quality Total: Sex-Specific** |
| --- | --- | --- | --- | --- | --- | --- | --- | --- |
| Al-Hakeim, 2020^1^ | 90 | 0 | IL-6 | Serum | DSM-IV MDD Diagnosis | Yes | 5 | 5 |
| Almeida, 2007^2^ | 4,245 | 0 | hsCRP | Serum | Geriatric Depression Scale | No | 8 | 7 |
| Andreasson, 2013^3*^ | 0 | 346 | IL-6, TNFα, IL-1β | Plasma | CES-D - Negative Affect Factor | No | 9 | 6 |
| Au, 2014^4^ | 0 | 3,130 | CRP | Serum | CES-D (8-item) | No | 11 | 4 |
| Azar, 2012^5*^ | 40 | 44 | hsCRP, IL-6 | Serum | BDI-II | No | 9 | 8 |
| Bell, 2017^6^ | 978 | 1,090 | hsCRP | Serum | CES-D (8-item) | No | 11 | 10 |
| Benson, 2008^7^ | 0 | 57 | hsCRP, IL-6 | Serum | BDI German Version | No | 5 | 4 |
| Beydoun, 2020^8^ | 776 | 988 | hsCRP, IL-6, IL-1β | Serum | CES-D | No | 11 | 8 |
| Boeck, 2018^9^ | 0 | 42 | CRP, IL-6, TNFα | Serum | DSM-IV MDD Diagnosis | Yes | 8 | 3 |
| Brambilla, 1998^10^ | 0 | 20 | IL-6, TNFα, IL-1β | Plasma | SCID-UP (DSM-III) | Yes | 5 | 5 |
| Campos‐Rodriguez, 2021^11^ | 0 | 247 | CRP, IL-6, TNFα | Plasma | Hospital Anxiety and Depression Scale Spanish Version | No | 10 | 10 |
| Capuron, 2008^12^ | 323 | 0 | hsCRP, IL-6, TNFα | Plasma | BDI-II | No | 11 | 10 |
| Capuron, 2011^13^ | 0 | 101 | hsCRP, IL-6 | Serum | NEO-PI-R Depression Facet Score French Version | No | 12 | 10 |
| Castellini, 2016^14^ | 3,212 | 0 | CRP | Serum | BDI-II | No | 9 | 8 |
| Cho, 2021^15^ | 4,746 | 5,956 | hsCRP | Serum | PHQ-9 Korean Version | No | 9 | 9 |
| Chocano-Bedoya, 2014^16^ | 0 | 4,403 | CRP, IL-6 | Plasma | Self-reported ever physician diagnosed and antidepressant use | No | 11 | 10 |
| Cizza, 2009^17^ | 0 | 118 | hsCRP | Plasma | DSM-IV MDD Diagnosis | Yes | 11 | 8 |
| Danner, 2003^18^ | 2,981 | 3,119 | CRP | Serum | Diagnostic Interview Schedule | Yes | 9 | 6 |
| Dixon, 2008^19^ | 0 | 377 | CRP | Serum | BDI | No | 9 | 8 |
| Dressler, 2006^20^ | 100 | 158 | CRP | Not reported | CES-D Brazilian Portuguese Version | No | 11 | 10 |
| Duivis, 2013^21^ | 950 | 1,911 | hsCRP | Plasma | Inventory of Depressive Symptomatology – Self Report | No | 12 | 11 |
| Elovainio, 2009^22^ | 2,748 | 3,257 | hsCRP | Serum | BDI | No | 9 | 9 |
| Empana, 2005^23^ | 889 | 0 | hsCRP, IL-6, Fibrinogen | Plasma | Minnesota Multiphasic Personality Inventory - Welsh Depression Subscale | No | 12 | 8 |
| Ernst, 2021^24^ | 5,450 | 4,670 | hsCRP | Plasma | PHQ-9 | No | 11 | 11 |
| Euteneuer, 2011^25^ | 34 | 51 | IL-6, TNFα | Serum | BDI-II German Version | No | 8 | 4 |
| Felger, 2020^26*^ | 31 | 58 | hsCRP, TNFα | Plasma | Inventory of Depressive Symptomatology – Self Report | No | 9 | 5 |
| Ford, 2004^27^ | 3,154 | 3,760 | CRP | Serum | Diagnostic Interview Schedule | Yes | 8 | 7 |
| Friedman, 2007^28^ | 0 | 112 | IL-6 | Plasma | Mood and Anxiety Symptom Questionnaire Short Form – Depressive Symptoms Scale | No | 9 | 8 |
| Gazal, 2013^29*^ | 0 | 11 | IL-6 | Serum | HAM-D | Yes | 7 | 4 |
| Gelaye, 2015^30^ | 315 | 201 | hsCRP | Serum | PHQ-9 | No | 8 | 7 |
| Ghahramani, 1996^31^ | 53 | 0 | Fibrinogen | Serum | BDI | No | 4 | 3 |
| Gimeno, 2009^32^ | 4,175 | 1,803 | hsCRP, IL-6 | Serum | General Health Questionnaire - Cognitive Symptoms of Depression | No | 13 | 11 |
| Glaus, 2014^33*^ | 1,684 | 1,843 | hsCRP, IL-6, TNFα, IL-1β | Serum | Diagnostic Interview for Genetic Studies French Version | Yes | 10 | 9 |
| Grassi-Oliveira, 2011^34^ | 0 | 30 | IL-6 | Plasma | BDI | No | 5 | 3 |
| Groer, 2015^35^ | 52 | 0 | hsCRP | Plasma | CES-D | No | 4 | 3 |
| Guo, 2018^36^ | 0 | 73 | CRP, IL-6, TNFα | Serum | HAM-D Chinese Version | Yes | 5 | 5 |
| Häfner, 2011^37^ | 847 | 700 | hsCRP, IL-6 | Serum | DEpression and EXhaustion subscale | No | 10 | 10 |
| Henningsson, 2008^38^ | 0 | 181 | hsCRP | Serum | Single Item (feel depressed) | No | 9 | 7 |
| Hiles, 2015^39^ | 699 | 711 | hsCRP, IL-6 | Serum | CES-D | No | 10 | 9 |
| Honkalampi, 2021^40^ | 152 | 253 | hsCRP | Not reported | SCID-IV (DSM-IV) | Yes | 8 | 5 |
| Huang, 2007^41^ | 24 | 30 | hsCRP | Serum | SCID-IV (DSM-IV) | Yes | 5 | 3 |
| Huang, 2019^42^ | 166 | 0 | hsCRP, IL-6 | Plasma | BDI-II | No | 13 | 12 |
| Imai, 2018^43*^ | 0 | 90 | hsCRP, IL-6, TNFα, IL-1β | Serum | BDI-II Japanese Version | No | 9 | 9 |
| Iodice, 2021^44*^ | 0 | 200 | IL-6, TNFα | Plasma | BDI-II | No | 7 | 7 |
| Janszky, 2005^45^ | 0 | 157 | hsCRP, IL-6 | Plasma | BDI | No | 8 | 8 |
| Johnson, 2017^46^ | 96 | 296 | TNFα | Serum | Geriatric Depression Scale | No | 5 | 3 |
| Jones, 2017^47^ | 0 | 7,477 | CRP | Serum | CES-D Short Form (6-item) | No | 10 | 10 |
| Joyce, 1992^48^ | 21 | 0 | CRP | Plasma | HAM-D | Yes | 4 | 4 |
| Kahl, 2005^49^ | 0 | 38 | IL-6, TNFα | Serum | SCID-IV (DSM-IV) German Version | Yes | 5 | 3 |
| Kahl, 2006^50^ | 0 | 24 | IL-1β | Serum | SCID-IV (DSM-IV) German Version | Yes | 10 | 4 |
| Keeler, 2021^51^ | 0 | 131 | IL-6, TNFα | Serum | BDI-II | No | 10 | 9 |
| Khan, 2020^52^ | 18,524 | 8,114 | hsCRP | Not reported | CES-D (10-item) | No | 9 | 8 |
| Kim, 2021^53^ | 47 | 53 | CRP, IL-6, IL-1β | Plasma | SCID-IV (DSM-IV) | Yes | 5 | 4 |
| Köhler-Forsberg, 2017^54^ | 89 | 142 | hsCRP | Serum | BDI | No | 10 | 10 |
| Ladwig, 2003^55^ | 3,205 | 0 | hsCRP | Serum | Von Zerssen Affective Symptom Checklist – Depressive Subscale | No | 9 | 6 |
| Lahlou-Laforet, 2006^56^ | 231 | 0 | Fibrinogen | Plasma | CES-D French Version | No | 7 | 4 |
| Lamers, 2019^57^ | 297 | 584 | IL-6 | Plasma | CIDI for DSM-IV | Yes | 13 | 12 |
| Lavebratt, 2017^58^ | 40 | 76 | IL-6 | Serum | MADRS | Yes | 11 | 6 |
| Lawes, 2019^59^ | 2,449 | 0 | hsCRP | Plasma | CES-D (8-item) | No | 11 | 6 |
| Lehtimäki, 2008^60^ | 0 | 6 | IL-6, IL-1β | Plasma | MADRS | Yes | 2 | 1 |
| Liu, 2014^61^ | 6,396 | 6,610 | hsCRP | Not reported | PHQ-9 | No | 8 | 8 |
| Liu, 2022^62^ | 365 | 425 | CRP, IL-6, TNFα, Fibrinogen | Serum | CES-D | No | 11 | 9 |
| Liukkonen, 2006^63^ | 2,559 | 2,447 | hsCRP | Serum | Hopkins Symptom Checklist-25 Depression Subscale | No | 9 | 9 |
| Liukkonen, 2010^64^ | 0 | 492 | hsCRP | Serum | BDI Finnish Version | No | 9 | 7 |
| Low, 2009^65^ | 0 | 173 | CRP | Not reported | CES-D | No | 6 | 4 |
| Luukinen, 2010^66^ | 156 | 248 | hsCRP | Serum | Short Zung Self-Rating Depression Scale | No | 10 | 9 |
| Ma, 2010^67^ | 259 | 249 | hsCRP | Serum | BDI | No | 12 | 12 |
| Marques-Deak, 2007^68^ | 0 | 85 | IL-6, IL-1β | Serum | SCID-IV (DSM-IV) | Yes | 8 | 7 |
| Martin, 2015^69^ | 876 | 0 | hsCRP, IL-6, TNFα | Not reported | BDI | No | 11 | 8 |
| Matthews, 2007^70^ | 0 | 3,080 | hsCRP, Fibrinogen | Plasma | CES-D | No | 12 | 12 |
| Matthews, 2010^71^ | 0 | 1,781 | hsCRP | Plasma | CES-D | No | 11 | 11 |
| McDade, 2013^72^ | 0 | 1,566 | hsCRP | Plasma | CES-D | No | 8 | 7 |
| McLaughlin, 2022^73*^ | 72 | 144 | hsCRP | Serum | SCID-5 (DSM-5) | Yes | 8 | 6 |
| Mehta, 2022^74^ | 0 | 54 | hsCRP, IL-6 | Plasma | BDI | No | 6 | 3 |
| Miller, 2005^75^ | 0 | 72 | hsCRP, IL-6, TNFα | Serum | Depression Interview and Structured Hamilton | Yes | 7 | 4 |
| Mommersteeg, 2019^76*^ | 247 | 273 | hsCRP | Serum | BDI | No | 11 | 11 |
| Morris, 2011^77^ | 199 | 313 | hsCRP | Plasma | BDI-II | No | 8 | 8 |
| Motivala, 2005^78^ | 40 | 0 | IL-6 | Serum | DSM-IV MDD Diagnosis | Yes | 8 | 7 |
| Naghashpour, 2011^79^ | 0 | 98 | hsCRP | Serum | BDI (13-item) | No | 6 | 5 |
| Naicker, 2017^80^ | 1,074 | 961 | CRP | Serum | CONOR-Mental Health Index | No | 10 | 10 |
| Ogłodek, 2014^81^ | 80 | 80 | IL-6 | Plasma | BDI-II | No | 4 | 3 |
| Ogłodek, 2017^82^ | 110 | 110 | TNFα | Serum | ICD-10 MDD Diagnosis | Yes | 5 | 4 |
| Ogłodek, 2022^83^ | 110 | 110 | IL-1β | Serum | BDI-II | No | 5 | 4 |
| Olszanecka-Glinianowicz, 2009^84^ | 0 | 62 | IL-6, TNFα | Serum | BDI | No | 5 | 4 |
| Osler, 2015^85*^ | 2,482 | 0 | hsCRP, IL-6 | Plasma | Major Depression Inventory | No | 9 | 8 |
| Panagiotakos, 2004^86^ | 453 | 400 | hsCRP | Not reported | Zung Self-Rating Depression Scale Greek Version | No | 10 | 9 |
| Pasco, 2010^87^ | 0 | 644 | hsCRP | Serum | SCID-IV (DSM-IV) Research Version | Yes | 10 | 10 |
| Penninx, 2003^88^ | 1,463 | 1,561 | IL-6 | Plasma | CES-D | No | 10 | 9 |
| Pike, 2006^89^ | 50 | 0 | IL-6 | Serum | SCID-IV (DSM-IV) | Yes | 6 | 4 |
| Poudel-Tandukar, 2014^90^ | 181 | 135 | CRP | Serum | BDI Nepali Version | No | 10 | 10 |
| Powers, 2019^91^ | 0 | 55 | hsCRP | Serum | BDI-II | No | 7 | 6 |
| Prohan, 2014^92^ | 60 | 0 | hsCRP | Serum | BDI-II | No | 6 | 4 |
| Ravona-Springer, 2020^93^ | 463 | 0 | CRP | Plasma | Geriatric Depression Scale | No | 10 | 10 |
| Redwine, 2007^94^ | 18 | 0 | IL-6 | Plasma | HAM-D | Yes | 9 | 5 |
| Schrock, 2021^95^ | 518 | 0 | CRP, IL-6, TNFα, IL-1β | Plasma | PROMIS Depression Short Form 8a | No | 12 | 12 |
| Shimura, 2017^96^ | 0 | 115 | IL-6 | Serum | Zung Self-Rating Depression Scale | No | 9 | 8 |
| Shin, 2019^97*^ | 496 | 645 | CRP | Not reported | CIDI-Auto 2.1 (DSM-IV) | Yes | 7 | 4 |
| Shirom, 2010^98^ | 538 | 203 | hsCRP, Fibrinogen | Serum | PHQ-8 | No | 10 | 9 |
| Smagula, 2014^99^ | 2,560 | 0 | CRP, IL-6, TNFα | Serum | Geriatric Depression Scale | No | 10 | 10 |
| Song, 2015^100^ | 224 | 345 | CRP | Serum | CES-D Korean Version | No | 8 | 7 |
| Song, 2022^101^ | 443 | 525 | CRP, IL-6 | Serum | CIDI-Short Form | Yes | 11 | 10 |
| Sturgeon, 2014^102^ | 0 | 34 | IL-6, TNFα | Serum | CES-D | No | 4 | 4 |
| Suarez, 2003^103^ | 95 | 0 | IL-6 | Plasma | BDI | No | 10 | 10 |
| Surtees, 2008^104^ | 1,483 | 845 | CRP | Plasma | Health and Life Experiences Questionnaire for DSM-IV MDD diagnosis | No | 10 | 7 |
| Tayefi, 2017^105^ | 3,012 | 3,969 | hsCRP | Serum | BDI | No | 9 | 8 |
| Toker, 2005^106^ | 933 | 630 | hsCRP, Fibrinogen | Plasma | PHQ-9 | No | 9 | 8 |
| Torres, 2022^107^ | 0 | 85 | hsCRP | Plasma | Inventory of Depressive Symptomatology – Self Report | No | 6 | 5 |
| Tsuboi, 2018^108^ | 0 | 20 | hsCRP, IL-6, TNFα | Serum | CES-D Japanese Version | No | 5 | 2 |
| Tully, 2015^109^ | 1,167 | 0 | hsCRP, IL-6, TNFα | Not reported | CES-D or BDI | No | 11 | 11 |
| Tully, 2016^110^ | 688 | 0 | hsCRP, IL-6 | Not reported | BDI | No | 11 | 7 |
| Ushiroyama, 2002^111^ | 0 | 610 | IL-6 | Plasma | Depressed mood (not specified) | No | 5 | 4 |
| Vetter, 2013^112^ | 79 | 311 | hsCRP | Not reported | PHQ-8 | No | 9 | 9 |
| Vogelzangs, 2012^113^ | 562 | 1,064 | IL-6, TNFα | Plasma | CIDI for DSM-IV | Yes | 10 | 10 |
| Wang, 2018^114^ | 120 | 0 | CRP | Plasma | HAM-D | Yes | 5 | 4 |
| Webb, 2017^115^ | 346 | 293 | CRP, IL-6, TNFα | Plasma | World Health Organization-Five Well-Being Index | No | 9 | 9 |
| Whooley, 2007^116^ | 801 | 183 | hsCRP, IL-6, Fibrinogen | Plasma or serum | Computerized Diagnostic Interview Schedule for DSM-IV | Yes | 12 | 10 |
| Wium-Andersen, 2014^117^ | 35,044 | 43,765 | hsCRP | Plasma | Hospitalization with depression (ICD-8 and ICD-10 MDD diagnoses) | Yes | 10 | 9 |
| Wysokiński, 2016^118^ | 54 | 350 | CRP | Serum | ICD-10 MDD diagnosis | Yes | 5 | 4 |
| Yang, 2007^119^ | 16 | 40 | IL-6, TNFα, IL-1β | Serum | DSM-IV MDD diagnosis and HAM-D Chinese Version | Yes | 6 | 6 |
| Yang, 2020^120^ | 142 | 250 | hsCRP | Serum | PHQ-9 | No | 8 | 8 |
| Ye, 2021^121^ | 63,574 | 81,964 | hsCRP | Serum | PHQ-9 | No | 8 | 8 |
| Zahra, 2007^122^ | 40 | 0 | hsCRP, IL-6, IL-1β | Plasma | DSM-IV MDD Diagnosis | Yes | 4 | 3 |
| Zhang, 2018^123^ | 4,404 | 5,055 | CRP | Not reported | CES-D (10-item) Chinese Version | No | 6 | 5 |
| Zheng, 2023^124^ | 1,949 | 1,739 | hsCRP | Not reported | CES-D (10-item) Chinese Version | No | 13 | 12 |

BDI – Beck Depression Inventory; CES-D – Center for Epidemiologic Studies Depression Scale; CIDI - Composite International Diagnostic Interview; CRP – C-reactive protein; DSM – Diagnostic and Statistical Manual of Mental Disorders; HAM-D - Hamilton Depression Rating Scale; hsCRP – high-sensitivity C-reactive protein; ICD - International Classification of Diseases; IL – interleukin; MADRS - Montgomery–Åsberg Depression Rating Scale; MDD – major depressive disorder; NEO-PI-R - Neuroticism, Extraversion and Openness Personality Inventory-Revised; PHQ – Patient Health Questionnaire; PROMIS - Patient-Reported Outcomes Measurement Information System; SCID - Structured Clinical Interview for DSM Disorders; TNFα – tumor necrosis factor alpha

Information reflects the sample sizes, biomarkers, fluid, and depression measure that were available and utilized in the sex-specific meta-analyses (e.g., only relevant biomarkers are listed, the depression measure used in sex-specific depression-marker relationship is listed). Superscript number references refer to “List of Included Papers”. ^*^ Indicates data or information was received from paper authors

### Table S4: Quality of included studies

| **First Author, Year** | 1A) Stated Aim: General | 1B) Stated Aim: Sex-Specific | 2)  Inclusion Exclusion Criteria | 3A)  Pre-register: General | 3B)  Pre-register: Sex-Specific | 4A) Study Design: General | 4B)  Study Design: Sex-Specific | 5A) Missing Data: General | 5B) Missing Data: Sex-Specific | 6)  Assay Reliability | 7A) Confounds: General | 7B) Confounds: Sex-Specific | 8A) Sample Size: General | 8B) Sample Size: Sex-Specific | Total: General | Total: Sex-Specific |
| --- | --- | --- | --- | --- | --- | --- | --- | --- | --- | --- | --- | --- | --- | --- | --- | --- |
| Al-Hakeim, 2020^1^ | 2 | 2 | 2 | 0 | 0 | 0 | 0 | 0 | 0 | 0 | 0 | 0 | 1 | 1 | 5 | 5 |
| Almeida, 2007^2^ | 2 | 1 | 1 | 0 | 0 | 0 | 0 | 1 | 1 | 0 | 2 | 2 | 2 | 2 | 8 | 7 |
| Andreasson, 2013^3^ | 2 | 1 | 2 | 0 | 0 | 0 | 0 | 2 | 2 | 0 | 2 | 0 | 1 | 1 | 9 | 6 |
| Au, 2014^4^ | 2 | 1 | 1 | 0 | 0 | 2 | 0 | 2 | 0 | 0 | 2 | 0 | 2 | 2 | 11 | 4 |
| Azar, 2012^5^ | 2 | 2 | 2 | 0 | 0 | 0 | 0 | 2 | 2 | 2 | 1 | 0 | 0 | 0 | 9 | 8 |
| Bell, 2017^6^ | 2 | 1 | 2 | 0 | 0 | 2 | 2 | 1 | 1 | 0 | 2 | 2 | 2 | 2 | 11 | 10 |
| Benson, 2008^7^ | 2 | 2 | 2 | 0 | 0 | 0 | 0 | 0 | 0 | 0 | 1 | 0 | 0 | 0 | 5 | 4 |
| Beydoun, 2020^8^ | 2 | 2 | 2 | 0 | 0 | 2 | 2 | 2 | 1 | 0 | 2 | 1 | 1 | 0 | 11 | 8 |
| Boeck, 2018^9^ | 2 | 1 | 2 | 0 | 0 | 0 | 0 | 2 | 0 | 0 | 2 | 0 | 0 | 0 | 8 | 3 |
| Brambilla, 1998^10^ | 1 | 1 | 2 | 0 | 0 | 0 | 0 | 0 | 0 | 2 | 0 | 0 | 0 | 0 | 5 | 5 |
| Campos‐Rodriguez, 2021^11^ | 2 | 2 | 2 | 0 | 0 | 0 | 0 | 1 | 1 | 2 | 2 | 2 | 1 | 1 | 10 | 10 |
| Capuron, 2008^12^ | 2 | 1 | 2 | 0 | 0 | 0 | 0 | 2 | 2 | 2 | 2 | 2 | 1 | 1 | 11 | 10 |
| Capuron, 2011^13^ | 2 | 2 | 2 | 0 | 0 | 2 | 0 | 1 | 1 | 2 | 2 | 2 | 1 | 1 | 12 | 10 |
| Castellini, 2016^14^ | 2 | 1 | 2 | 0 | 0 | 0 | 0 | 1 | 1 | 0 | 2 | 2 | 2 | 2 | 9 | 8 |
| Cho, 2021^15^ | 2 | 2 | 2 | 0 | 0 | 0 | 0 | 1 | 1 | 0 | 2 | 2 | 2 | 2 | 9 | 9 |
| Chocano-Bedoya, 2014^16^ | 2 | 1 | 2 | 0 | 0 | 2 | 2 | 1 | 1 | 0 | 2 | 2 | 2 | 2 | 11 | 10 |
| Cizza, 2009^17^ | 2 | 2 | 2 | 0 | 0 | 2 | 0 | 1 | 1 | 2 | 1 | 0 | 1 | 1 | 11 | 8 |
| Danner, 2003^18^ | 2 | 1 | 2 | 0 | 0 | 0 | 0 | 1 | 1 | 0 | 2 | 0 | 2 | 2 | 9 | 6 |
| Dixon, 2008^19^ | 2 | 1 | 2 | 0 | 0 | 0 | 0 | 2 | 2 | 0 | 2 | 2 | 1 | 1 | 9 | 8 |
| Dressler, 2006^20^ | 2 | 1 | 2 | 0 | 0 | 0 | 0 | 2 | 2 | 2 | 2 | 2 | 1 | 1 | 11 | 10 |
| Duivis, 2013^21^ | 2 | 1 | 2 | 0 | 0 | 0 | 0 | 2 | 2 | 2 | 2 | 2 | 2 | 2 | 12 | 11 |
| Elovainio, 2009^22^ | 2 | 2 | 2 | 0 | 0 | 0 | 0 | 1 | 1 | 0 | 2 | 2 | 2 | 2 | 9 | 9 |
| Empana, 2005^23^ | 2 | 2 | 2 | 0 | 0 | 2 | 0 | 2 | 2 | 0 | 2 | 0 | 2 | 2 | 12 | 8 |
| Ernst, 2021^24^ | 2 | 2 | 2 | 0 | 0 | 2 | 2 | 1 | 1 | 0 | 2 | 2 | 2 | 2 | 11 | 11 |
| Euteneuer, 2011^25^ | 2 | 1 | 2 | 0 | 0 | 0 | 0 | 1 | 1 | 0 | 2 | 0 | 1 | 0 | 8 | 4 |
| Felger, 2020^26^ | 2 | 1 | 2 | 0 | 0 | 0 | 0 | 0 | 0 | 2 | 2 | 0 | 1 | 0 | 9 | 5 |
| Ford, 2004^27^ | 2 | 1 | 1 | 0 | 0 | 0 | 0 | 1 | 1 | 0 | 2 | 2 | 2 | 2 | 8 | 7 |
| Friedman, 2007^28^ | 2 | 1 | 1 | 0 | 0 | 0 | 0 | 1 | 1 | 2 | 2 | 2 | 1 | 1 | 9 | 8 |
| Gazal, 2013^29^ | 2 | 1 | 2 | 0 | 0 | 2 | 0 | 0 | 0 | 1 | 0 | 0 | 0 | 0 | 7 | 4 |
| Gelaye, 2015^30^ | 2 | 1 | 2 | 0 | 0 | 0 | 0 | 1 | 1 | 0 | 2 | 2 | 1 | 1 | 8 | 7 |
| Ghahramani, 1996^31^ | 2 | 1 | 1 | 0 | 0 | 0 | 0 | 1 | 1 | 0 | 0 | 0 | 0 | 0 | 4 | 3 |
| Gimeno, 2009^32^ | 2 | 1 | 2 | 0 | 0 | 2 | 2 | 1 | 1 | 2 | 2 | 1 | 2 | 2 | 13 | 11 |
| Glaus, 2014^33^ | 2 | 1 | 2 | 0 | 0 | 0 | 0 | 1 | 1 | 2 | 1 | 1 | 2 | 2 | 10 | 9 |
| Grassi-Oliveira, 2011^34^ | 2 | 1 | 2 | 0 | 0 | 0 | 0 | 0 | 0 | 0 | 1 | 0 | 0 | 0 | 5 | 3 |
| Groer, 2015^35^ | 2 | 1 | 1 | 0 | 0 | 0 | 0 | 0 | 0 | 1 | 0 | 0 | 0 | 0 | 4 | 3 |
| Guo, 2018^36^ | 1 | 1 | 2 | 0 | 0 | 0 | 0 | 0 | 0 | 0 | 2 | 2 | 0 | 0 | 5 | 5 |
| Häfner, 2011^37^ | 2 | 2 | 2 | 0 | 0 | 0 | 0 | 1 | 1 | 2 | 2 | 2 | 1 | 1 | 10 | 10 |
| Henningsson, 2008^38^ | 2 | 1 | 2 | 0 | 0 | 0 | 0 | 1 | 1 | 2 | 1 | 0 | 1 | 1 | 9 | 7 |
| Hiles, 2015^39^ | 2 | 1 | 2 | 0 | 0 | 2 | 2 | 1 | 1 | 0 | 2 | 2 | 1 | 1 | 10 | 9 |
| Honkalampi, 2021^40^ | 2 | 1 | 1 | 0 | 0 | 0 | 0 | 2 | 2 | 0 | 2 | 0 | 1 | 1 | 8 | 5 |
| Huang, 2007^41^ | 2 | 1 | 2 | 0 | 0 | 0 | 0 | 0 | 0 | 0 | 1 | 0 | 0 | 0 | 5 | 3 |
| Huang, 2019^42^ | 2 | 1 | 2 | 0 | 0 | 2 | 2 | 2 | 2 | 2 | 2 | 2 | 1 | 1 | 13 | 12 |
| Imai, 2018^43^ | 2 | 2 | 2 | 0 | 0 | 0 | 0 | 2 | 2 | 2 | 0 | 0 | 1 | 1 | 9 | 9 |
| Iodice, 2021^44^ | 2 | 2 | 2 | 0 | 0 | 0 | 0 | 0 | 0 | 0 | 1 | 1 | 2 | 2 | 7 | 7 |
| Janszky, 2005^45^ | 2 | 2 | 2 | 0 | 0 | 0 | 0 | 1 | 1 | 0 | 2 | 2 | 1 | 1 | 8 | 8 |
| Johnson, 2017^46^ | 2 | 1 | 1 | 0 | 0 | 0 | 0 | 0 | 0 | 0 | 1 | 0 | 1 | 1 | 5 | 3 |
| Jones, 2017^47^ | 2 | 2 | 2 | 0 | 0 | 2 | 2 | 0 | 0 | 0 | 2 | 2 | 2 | 2 | 10 | 10 |
| Joyce, 1992^48^ | 2 | 2 | 2 | 0 | 0 | 0 | 0 | 0 | 0 | 0 | 0 | 0 | 0 | 0 | 4 | 4 |
| Kahl, 2005^49^ | 2 | 1 | 2 | 0 | 0 | 0 | 0 | 0 | 0 | 0 | 1 | 0 | 0 | 0 | 5 | 3 |
| Kahl, 2006^50^ | 2 | 0 | 2 | 0 | 0 | 0 | 0 | 0 | 0 | 2 | 2 | 0 | 2 | 0 | 10 | 4 |
| Keeler, 2021^51^ | 2 | 1 | 2 | 0 | 0 | 0 | 0 | 2 | 2 | 0 | 2 | 2 | 2 | 2 | 10 | 9 |
| Khan, 2020^52^ | 2 | 1 | 2 | 0 | 0 | 0 | 0 | 1 | 1 | 0 | 2 | 2 | 2 | 2 | 9 | 8 |
| Kim, 2021^53^ | 2 | 2 | 2 | 0 | 0 | 0 | 0 | 0 | 0 | 0 | 0 | 0 | 1 | 0 | 5 | 4 |
| Köhler-Forsberg, 2017^54^ | 2 | 2 | 2 | 0 | 0 | 0 | 0 | 1 | 1 | 2 | 2 | 2 | 1 | 1 | 10 | 10 |
| Ladwig, 2003^55^ | 2 | 1 | 2 | 0 | 0 | 0 | 0 | 1 | 1 | 0 | 2 | 0 | 2 | 2 | 9 | 6 |
| Lahlou-Laforet, 2006^56^ | 2 | 1 | 2 | 0 | 0 | 0 | 0 | 0 | 0 | 0 | 2 | 0 | 1 | 1 | 7 | 4 |
| Lamers, 2019^57^ | 2 | 1 | 2 | 0 | 0 | 2 | 2 | 1 | 1 | 2 | 2 | 2 | 2 | 2 | 13 | 12 |
| Lavebratt, 2017^58^ | 2 | 1 | 2 | 0 | 0 | 2 | 0 | 1 | 1 | 2 | 2 | 0 | 0 | 0 | 11 | 6 |
| Lawes, 2019^59^ | 2 | 1 | 2 | 0 | 0 | 2 | 0 | 1 | 1 | 0 | 2 | 0 | 2 | 2 | 11 | 6 |
| Lehtimäki, 2008^60^ | 2 | 1 | 0 | 0 | 0 | 0 | 0 | 0 | 0 | 0 | 0 | 0 | 0 | 0 | 2 | 1 |
| Liu, 2014^61^ | 1 | 1 | 2 | 0 | 0 | 0 | 0 | 1 | 1 | 0 | 2 | 2 | 2 | 2 | 8 | 8 |
| Liu, 2022^62^ | 2 | 1 | 2 | 0 | 0 | 0 | 0 | 1 | 1 | 2 | 2 | 2 | 2 | 1 | 11 | 9 |
| Liukkonen, 2006^63^ | 2 | 2 | 2 | 0 | 0 | 0 | 0 | 1 | 1 | 0 | 2 | 2 | 2 | 2 | 9 | 9 |
| Liukkonen, 2010^64^ | 2 | 2 | 2 | 0 | 0 | 0 | 0 | 2 | 2 | 0 | 2 | 0 | 1 | 1 | 9 | 7 |
| Low, 2009^65^ | 2 | 2 | 1 | 0 | 0 | 0 | 0 | 0 | 0 | 0 | 2 | 0 | 1 | 1 | 6 | 4 |
| Luukinen, 2010^66^ | 2 | 1 | 2 | 0 | 0 | 2 | 2 | 1 | 1 | 0 | 2 | 2 | 1 | 1 | 10 | 9 |
| Ma, 2010^67^ | 2 | 2 | 2 | 0 | 0 | 2 | 2 | 1 | 1 | 2 | 2 | 2 | 1 | 1 | 12 | 12 |
| Marques-Deak, 2007^68^ | 2 | 2 | 2 | 0 | 0 | 1 | 0 | 1 | 1 | 1 | 0 | 0 | 1 | 1 | 8 | 7 |
| Martin, 2015^69^ | 2 | 2 | 2 | 0 | 0 | 2 | 2 | 1 | 1 | 0 | 2 | 0 | 2 | 1 | 11 | 8 |
| Matthews, 2007^70^ | 2 | 2 | 2 | 0 | 0 | 2 | 2 | 2 | 2 | 0 | 2 | 2 | 2 | 2 | 12 | 12 |
| Matthews, 2010^71^ | 2 | 2 | 2 | 0 | 0 | 2 | 2 | 1 | 1 | 0 | 2 | 2 | 2 | 2 | 11 | 11 |
| McDade, 2013^72^ | 2 | 1 | 2 | 0 | 0 | 0 | 0 | 0 | 0 | 0 | 2 | 2 | 2 | 2 | 8 | 7 |
| McLaughlin, 2022^73^ | 2 | 1 | 2 | 0 | 0 | 0 | 0 | 0 | 0 | 2 | 1 | 0 | 1 | 1 | 8 | 6 |
| Mehta, 2022^74^ | 2 | 1 | 2 | 0 | 0 | 0 | 0 | 0 | 0 | 0 | 2 | 0 | 0 | 0 | 6 | 3 |
| Miller, 2005^75^ | 2 | 1 | 2 | 0 | 0 | 0 | 0 | 0 | 0 | 1 | 2 | 0 | 0 | 0 | 7 | 4 |
| Mommersteeg, 2019^76^ | 2 | 2 | 2 | 2 | 2 | 0 | 0 | 2 | 2 | 0 | 2 | 2 | 1 | 1 | 11 | 11 |
| Morris, 2011^77^ | 2 | 2 | 2 | 0 | 0 | 0 | 0 | 1 | 1 | 0 | 2 | 2 | 1 | 1 | 8 | 8 |
| Motivala, 2005^78^ | 2 | 1 | 2 | 0 | 0 | 0 | 0 | 1 | 1 | 2 | 1 | 1 | 0 | 0 | 8 | 7 |
| Naghashpour, 2011^79^ | 2 | 1 | 2 | 0 | 0 | 0 | 0 | 0 | 0 | 0 | 0 | 0 | 2 | 2 | 6 | 5 |
| Naicker, 2017^80^ | 2 | 2 | 2 | 0 | 0 | 0 | 0 | 2 | 2 | 0 | 2 | 2 | 2 | 2 | 10 | 10 |
| Ogłodek, 2014^81^ | 1 | 1 | 2 | 0 | 0 | 0 | 0 | 0 | 0 | 0 | 0 | 0 | 1 | 0 | 4 | 3 |
| Ogłodek, 2017^82^ | 2 | 1 | 2 | 0 | 0 | 0 | 0 | 0 | 0 | 0 | 0 | 0 | 1 | 1 | 5 | 4 |
| Ogłodek, 2022^83^ | 2 | 1 | 2 | 0 | 0 | 0 | 0 | 0 | 0 | 0 | 0 | 0 | 1 | 1 | 5 | 4 |
| Olszanecka-Glinianowicz, 2009^84^ | 2 | 1 | 2 | 0 | 0 | 0 | 0 | 0 | 0 | 1 | 0 | 0 | 0 | 0 | 5 | 4 |
| Osler, 2015^85^ | 2 | 2 | 2 | 0 | 0 | 1 | 0 | 0 | 0 | 0 | 2 | 2 | 2 | 2 | 9 | 8 |
| Panagiotakos, 2004^86^ | 2 | 2 | 2 | 0 | 0 | 0 | 0 | 2 | 2 | 0 | 2 | 2 | 2 | 1 | 10 | 9 |
| Pasco, 2010^87^ | 2 | 2 | 2 | 0 | 0 | 2 | 2 | 1 | 1 | 0 | 2 | 2 | 1 | 1 | 10 | 10 |
| Penninx, 2003^88^ | 2 | 1 | 2 | 0 | 0 | 0 | 0 | 2 | 2 | 0 | 2 | 2 | 2 | 2 | 10 | 9 |
| Pike, 2006^89^ | 2 | 1 | 2 | 0 | 0 | 0 | 0 | 0 | 0 | 1 | 1 | 0 | 0 | 0 | 6 | 4 |
| Poudel-Tandukar, 2014^90^ | 2 | 2 | 2 | 0 | 0 | 0 | 0 | 2 | 2 | 1 | 2 | 2 | 1 | 1 | 10 | 10 |
| Powers, 2019^91^ | 2 | 1 | 2 | 0 | 0 | 0 | 0 | 0 | 0 | 2 | 1 | 1 | 0 | 0 | 7 | 6 |
| Prohan, 2014^92^ | 2 | 2 | 2 | 0 | 0 | 0 | 0 | 0 | 0 | 0 | 2 | 0 | 0 | 0 | 6 | 4 |
| Ravona-Springer, 2020^93^ | 2 | 2 | 2 | 0 | 0 | 1 | 1 | 2 | 2 | 0 | 2 | 2 | 1 | 1 | 10 | 10 |
| Redwine, 2007^94^ | 2 | 1 | 2 | 0 | 0 | 1 | 0 | 0 | 0 | 2 | 0 | 0 | 2 | 0 | 9 | 5 |
| Schrock, 2021^95^ | 2 | 2 | 1 | 0 | 0 | 2 | 2 | 2 | 2 | 2 | 2 | 2 | 1 | 1 | 12 | 12 |
| Shimura, 2017^96^ | 2 | 1 | 2 | 0 | 0 | 0 | 0 | 0 | 0 | 2 | 2 | 2 | 1 | 1 | 9 | 8 |
| Shin, 2019^97^ | 2 | 1 | 1 | 0 | 0 | 0 | 0 | 1 | 1 | 0 | 2 | 0 | 1 | 1 | 7 | 4 |
| Shirom, 2010^98^ | 2 | 1 | 2 | 0 | 0 | 2 | 2 | 1 | 1 | 0 | 2 | 2 | 1 | 1 | 10 | 9 |
| Smagula, 2014^99^ | 2 | 2 | 2 | 0 | 0 | 0 | 0 | 2 | 2 | 0 | 2 | 2 | 2 | 2 | 10 | 10 |
| Song, 2015^100^ | 2 | 1 | 2 | 0 | 0 | 0 | 0 | 1 | 1 | 0 | 2 | 2 | 1 | 1 | 8 | 7 |
| Song, 2022^101^ | 2 | 2 | 1 | 0 | 0 | 1 | 1 | 1 | 1 | 2 | 2 | 2 | 2 | 1 | 11 | 10 |
| Sturgeon, 2014^102^ | 2 | 2 | 2 | 0 | 0 | 0 | 0 | 0 | 0 | 0 | 0 | 0 | 0 | 0 | 4 | 4 |
| Suarez, 2003^103^ | 2 | 2 | 1 | 0 | 0 | 0 | 0 | 2 | 2 | 2 | 2 | 2 | 1 | 1 | 10 | 10 |
| Surtees, 2008^104^ | 2 | 1 | 2 | 0 | 0 | 0 | 0 | 2 | 2 | 0 | 2 | 0 | 2 | 2 | 10 | 7 |
| Tayefi, 2017^105^ | 2 | 1 | 1 | 0 | 0 | 0 | 0 | 2 | 2 | 0 | 2 | 2 | 2 | 2 | 9 | 8 |
| Toker, 2005^106^ | 2 | 1 | 2 | 0 | 0 | 0 | 0 | 2 | 2 | 0 | 2 | 2 | 1 | 1 | 9 | 8 |
| Torres, 2022^107^ | 2 | 2 | 1 | 0 | 0 | 0 | 0 | 1 | 1 | 0 | 1 | 0 | 1 | 1 | 6 | 5 |
| Tsuboi, 2018^108^ | 2 | 1 | 1 | 0 | 0 | 0 | 0 | 0 | 0 | 0 | 2 | 0 | 0 | 0 | 5 | 2 |
| Tully, 2015^109^ | 2 | 2 | 2 | 0 | 0 | 2 | 2 | 1 | 1 | 0 | 2 | 2 | 2 | 2 | 11 | 11 |
| Tully, 2016^110^ | 2 | 1 | 2 | 0 | 0 | 2 | 1 | 1 | 1 | 1 | 2 | 0 | 1 | 1 | 11 | 7 |
| Ushiroyama, 2002^111^ | 2 | 2 | 1 | 0 | 0 | 0 | 0 | 0 | 0 | 0 | 0 | 0 | 2 | 1 | 5 | 4 |
| Vetter, 2013^112^ | 2 | 2 | 2 | 0 | 0 | 0 | 0 | 2 | 2 | 0 | 2 | 2 | 1 | 1 | 9 | 9 |
| Vogelzangs, 2012^113^ | 2 | 2 | 2 | 0 | 0 | 0 | 0 | 1 | 1 | 2 | 2 | 2 | 1 | 1 | 10 | 10 |
| Wang, 2018^114^ | 2 | 1 | 2 | 0 | 0 | 0 | 0 | 0 | 0 | 0 | 0 | 0 | 1 | 1 | 5 | 4 |
| Webb, 2017^115^ | 2 | 2 | 2 | 0 | 0 | 0 | 0 | 1 | 1 | 1 | 2 | 2 | 1 | 1 | 9 | 9 |
| Whooley, 2007^116^ | 2 | 1 | 2 | 0 | 0 | 0 | 0 | 2 | 2 | 2 | 2 | 2 | 2 | 1 | 12 | 10 |
| Wium-Andersen, 2014^117^ | 2 | 1 | 2 | 0 | 0 | 0 | 0 | 2 | 2 | 0 | 2 | 2 | 2 | 2 | 10 | 9 |
| Wysokiński, 2016^118^ | 2 | 1 | 2 | 0 | 0 | 0 | 0 | 0 | 0 | 0 | 0 | 0 | 1 | 1 | 5 | 4 |
| Yang, 2007^119^ | 2 | 2 | 2 | 0 | 0 | 0 | 0 | 0 | 0 | 2 | 0 | 0 | 0 | 0 | 6 | 6 |
| Yang, 2020^120^ | 2 | 2 | 2 | 0 | 0 | 0 | 0 | 2 | 2 | 0 | 1 | 1 | 1 | 1 | 8 | 8 |
| Ye, 2021^121^ | 2 | 2 | 1 | 0 | 0 | 0 | 0 | 1 | 1 | 0 | 2 | 2 | 2 | 2 | 8 | 8 |
| Zahra, 2007^122^ | 2 | 1 | 2 | 0 | 0 | 0 | 0 | 0 | 0 | 0 | 0 | 0 | 0 | 0 | 4 | 3 |
| Zhang, 2018^123^ | 2 | 1 | 0 | 0 | 0 | 0 | 0 | 0 | 0 | 0 | 2 | 2 | 2 | 2 | 6 | 5 |
| Zheng, 2023^124^ | 2 | 1 | 2 | 0 | 0 | 2 | 2 | 1 | 1 | 2 | 2 | 2 | 2 | 2 | 13 | 12 |

Superscript number references refer to “List of Included Papers”

### List of Included Papers

1.	Al-Hakeim HK, Al-Fadhel SZ, Al-Dujaili AH. Correlation of serum cu and zn with some cytokines in major depressive disorder. *J Glob Pharma Technol*. 2020;12(6):115-121.

2.	Almeida OP, Norman P, Hankey GJ, Jamrozik K, Flicker L. The association between C-reactive protein concentration and depression in later life is due to poor physical health: results from the Health in Men Study (HIMS). *Psychol Med*. 2007;37(12):1775-1786. doi:10.1017/S0033291707000827

3.	Andreasson AN, Szulkin R, Undén AL, von Essen J, Nilsson LG, Lekander M. Inflammation and positive affect are associated with subjective health in women of the general population. *J Health Psychol*. 2013;18(3):311-320. doi:10.1177/1359105311435428

4.	Au B, Smith KJ, Gariépy G, Schmitz N. C-reactive protein, depressive symptoms, and risk of diabetes: Results from the English Longitudinal Study of Ageing (ELSA). *J Psychosom Res*. 2014;77(3):180-186. doi:10.1016/j.jpsychores.2014.07.012

5.	Azar R, Nolan RP, Stewart DE. Listening to the heart-brain talk: persistent depressive symptoms are associated with hsCRP in apparently healthy individuals at high risk for coronary artery disease. *Eur J Prev Cardiol*. 2012;19(4):857-863. doi:10.1177/1741826711415720

6.	Bell JA, Kivimäki M, Bullmore ET, Steptoe A, Carvalho LA. Repeated exposure to systemic inflammation and risk of new depressive symptoms among older adults. *Transl Psychiatry*. 2017;7(8):e1208. doi:10.1038/tp.2017.155

7.	Benson S, Janssen OE, Hahn S, et al. Obesity, depression, and chronic low-grade inflammation in women with polycystic ovary syndrome. *Brain Behav Immun*. 2008;22(2):177-184. doi:10.1016/j.bbi.2007.07.003

8.	Beydoun MA, Obhi HK, Weiss J, et al. Systemic inflammation is associated with depressive symptoms differentially by sex and race: a longitudinal study of urban adults. *Mol Psychiatry*. 2020;25(6):1286-1300. doi:10.1038/s41380-019-0408-2

9.	Boeck C, Pfister S, Bürkle A, et al. Alterations of the serum N-glycan profile in female patients with Major Depressive Disorder. *J Affect Disord*. 2018;234:139-147. doi:10.1016/j.jad.2018.02.082

10.	Brambilla F, Maggioni M. Blood levels of cytokines in elderly patients with major depressive disorder. *Acta Psychiatr Scand*. 1998;97(4):309-313. doi:10.1111/j.1600-0447.1998.tb10005.x

11.	Campos-Rodriguez F, Cordero-Guevara J, Asensio-Cruz MI, et al. Interleukin 6 as a marker of depression in women with sleep apnea. *J Sleep Res*. 2021;30(1):e13035. doi:10.1111/jsr.13035

12.	Capuron L, Su S, Miller AH, et al. Depressive symptoms and metabolic syndrome: Is inflammation the underlying link? *Biol Psychiatry*. 2008;64(10):896-900. doi:10.1016/j.biopsych.2008.05.019

13.	Capuron L, Poitou C, Machaux-Tholliez D, et al. Relationship between adiposity, emotional status and eating behaviour in obese women: role of inflammation. *Psychol Med*. 2011;41(7):1517-1528. doi:10.1017/S0033291710001984

14.	Castellini G, Wu FCW, Finn JD, et al. Interactions Between Depression and Lower Urinary Tract Symptoms: The Role of Adverse Life Events and Inflammatory Mechanisms. Results From the European Male Ageing Study. *Psychosom Med*. 2016;78(6):758-769. doi:10.1097/PSY.0000000000000328

15.	Cho SH, Lim JE, Lee J, et al. Association between high-sensitivity C-reactive protein levels and depression: Moderation by age, sex, obesity, and aerobic physical activity. *J Affect Disord*. 2021;291:375-383. doi:10.1016/j.jad.2021.05.040

16.	Chocano-Bedoya PO, Mirzaei F, O’Reilly EJ, et al. C-reactive protein, interleukin-6, soluble tumor necrosis factor α receptor 2 and incident clinical depression. *J Affect Disord*. 2014;163:25-32. doi:10.1016/j.jad.2014.03.023

17.	Cizza G, Eskandari F, Coyle M, et al. Plasma CRP levels in premenopausal women with major depression: a 12-month controlled study. *Horm Metab Res*. 2009;41(8):641-648. doi:10.1055/s-0029-1220717

18.	Danner M, Kasl SV, Abramson JL, Vaccarino V. Association between depression and elevated C-reactive protein. *Psychosom Med*. 2003;65(3):347-356. doi:10.1097/01.PSY.0000041542.29808.01

19.	Dixon JB, Hayden MJ, Lambert GW, et al. Raised CRP levels in obese patients: symptoms of depression have an independent positive association. *Obes (Silver Spring)*. 2008;16(9):2010-2015. doi:10.1038/oby.2008.271

20.	Dressler WW, Balieiro MC, Ribeiro RP, Dos-Santos JE. Depressive symptoms and C-reactive protein in a Brazilian urban community. *Braz J Med Biol Res*. 2006;39:1013-1019. doi:10.1590/S0100-879X2006000800003

21.	Duivis HE, Vogelzangs N, Kupper N, de Jonge P, Penninx BWJH. Differential association of somatic and cognitive symptoms of depression and anxiety with inflammation: Findings from the Netherlands Study of Depression and Anxiety (NESDA). *Psychoneuroendocrinology*. 2013;38(9):1573-1585. doi:10.1016/j.psyneuen.2013.01.002

22.	Elovainio M, Aalto AM, Kivimäki M, et al. Depression and C-reactive protein: Population-based Health 2000 Study. *Psychosom Med*. 2009;71(4):423-430. doi:10.1097/PSY.0b013e31819e333a

23.	Empana JP, Sykes DH, Luc G, et al. Contributions of depressive mood and circulating inflammatory markers to coronary heart disease in healthy European men: the Prospective Epidemiological Study of Myocardial Infarction (PRIME). *Circulation*. 2005;111(18):2299-2305. doi:10.1161/01.CIR.0000164203.54111.AE

24.	Ernst M, Brähler E, Otten D, et al. Inflammation predicts new onset of depression in men, but not in women within a prospective, representative community cohort. *Sci Rep*. 2021;11(1):2271. doi:10.1038/s41598-021-81927-9

25.	Euteneuer F, Schwarz MJ, Hennings A, et al. Depression, cytokines and experimental pain: evidence for sex-related association patterns. *J Affect Disord*. 2011;131(1-3):143-149. doi:10.1016/j.jad.2010.11.017

26.	Felger JC, Haroon E, Patel TA, et al. What does plasma CRP tell us about peripheral and central inflammation in depression? *Mol Psychiatry*. 2020;25(6):1301-1311. doi:10.1038/s41380-018-0096-3

27.	Ford DE, Erlinger TP. Depression and C-reactive protein in US adults: data from the Third National Health and Nutrition Examination Survey. *Arch Intern Med*. 2004;164(9):1010-1014. doi:10.1001/archinte.164.9.1010

28.	Friedman EM, Hayney M, Love GD, Singer BH, Ryff CD. Plasma interleukin-6 and soluble IL-6 receptors are associated with psychological well-being in aging women. *Health Psychol*. 2007;26(3):305-313. doi:10.1037/0278-6133.26.3.305

29.	Gazal M, Souza LD, Fucolo BA, et al. The impact of cognitive behavioral therapy on IL-6 levels in unmedicated women experiencing the first episode of depression: a pilot study. *Psychiatry Res*. 2013;209(3):742-745. doi:10.1016/j.psychres.2013.03.002

30.	Gelaye B, Williams MA, Lemma S, et al. Major depressive disorder and cardiometabolic disease risk among sub-Saharan African adults. *Diabetes Metab Syndr*. 2015;9(3):183-191. doi:10.1016/j.dsx.2014.05.003

31.	Ghahramani M, Nazari M, Tabei S, Ahmadi J, Rafati S, Ghalambor M. Effects of depression on endocrine and immune systems. *Med J Islam Repub Iran*. 1996;10(2):117-124.

32.	Gimeno D, Kivimäki M, Brunner EJ, et al. Associations of C-reactive protein and interleukin-6 with cognitive symptoms of depression: 12-year follow-up of the Whitehall II study. *Psychol Med*. 2009;39(3):413-423. doi:10.1017/S0033291708003723

33.	Glaus J, Vandeleur CL, von Känel R, et al. Associations between mood, anxiety or substance use disorders and inflammatory markers after adjustment for multiple covariates in a population-based study. *J Psychiatr Res*. 2014;58:36-45. doi:10.1016/j.jpsychires.2014.07.012

34.	Grassi-Oliveira R, Bauer ME, Pezzi JC, Teixeira AL, Brietzke E. Interleukin-6 and verbal memory in recurrent major depressive disorder. *Neuro Endocrinol Lett*. 2011;32(4):540-544.

35.	Groer MW, Kane B, Williams SN, Duffy A. Relationship of PTSD Symptoms With Combat Exposure, Stress, and Inflammation in American Soldiers. *Biol Res Nurs*. 2015;17(3):303-310. doi:10.1177/1099800414544949

36.	Guo L, Ren L, Zhang C. Relationship between depression and inflammatory factors and brain-derived neurotrophic factor in patients with perimenopause syndrome. *Exp Ther Med*. 2018;15(5):4436-4440. doi:10.3892/etm.2018.5985

37.	Häfner S, Emeny RT, Lacruz ME, et al. Association between social isolation and inflammatory markers in depressed and non-depressed individuals: results from the MONICA/KORA study. *Brain Behav Immun*. 2011;25(8):1701-1707. doi:10.1016/j.bbi.2011.06.017

38.	Henningsson S, Baghaei F, Rosmond R, et al. Association between serum levels of C-reactive protein and personality traits in women. *Behav Brain Funct*. 2008;4(1):16. doi:10.1186/1744-9081-4-16

39.	Hiles SA, Baker AL, de Malmanche T, McEvoy M, Boyle M, Attia J. Unhealthy lifestyle may increase later depression via inflammation in older women but not men. *J Psychiatr Res*. 2015;63:65-74. doi:10.1016/j.jpsychires.2015.02.010

40.	Honkalampi K, Virtanen M, Hintsa T, et al. Comparison of the level of allostatic load between patients with major depression and the general population. *J Psychosom Res*. 2021;143:110389. doi:10.1016/j.jpsychores.2021.110389

41.	Huang TL, Lin FC. High-sensitivity C-reactive protein levels in patients with major depressive disorder and bipolar mania. *Prog Neuropsychopharmacol Biol Psychiatry*. 2007;31(2):370-372. doi:10.1016/j.pnpbp.2006.09.010

42.	Huang M, Su S, Goldberg J, et al. Longitudinal association of inflammation with depressive symptoms: A 7-year cross-lagged twin difference study. *Brain Behav Immun*. 2019;75:200-207. doi:10.1016/j.bbi.2018.10.007

43.	Imai R, Hori H, Itoh M, et al. Inflammatory markers and their possible effects on cognitive function in women with posttraumatic stress disorder. *J Psychiatr Res*. 2018;102:192-200. doi:10.1016/j.jpsychires.2018.04.009

44.	Iodice S, Ceresa A, Esposito CM, et al. The Independent Role of Body Mass Index (BMI) and Severity of Depressive Symptoms on Biological Changes of Women Affected by Overweight/Obesity. *Int J Environ Res Public Health*. 2021;18(6):2923. doi:10.3390/ijerph18062923

45.	Janszky I, Lekander M, Blom M, Georgiades A, Ahnve S. Self-rated health and vital exhaustion, but not depression, is related to inflammation in women with coronary heart disease. *Brain Behav Immun*. 2005;19(6):555-563. doi:10.1016/j.bbi.2005.01.001

46.	Johnson LA, Edwards M, Gamboa A, Hall J, Robinson M, O’Bryant SE. Depression, inflammation, and memory loss among Mexican Americans: analysis of the HABLE cohort. *Int Psychogeriatr*. 2017;29(10):1693-1699. doi:10.1017/S1041610217001016

47.	Jones SM, Weitlauf J, Danhauer SC, et al. Prospective data from the Women’s Health Initiative on depressive symptoms, stress, and inflammation. *J Health Psychol*. 2017;22(4):457-464. doi:10.1177/1359105315603701

48.	Joyce PR, Hawes CR, Mulder RT, Sellman JD, Wilson DA, Boswell DR. Elevated levels of acute phase plasma proteins in major depression. *Biol Psychiatry*. 1992;32(11):1035-1041. doi:10.1016/0006-3223(92)90065-8

49.	Kahl KG, Bester M, Greggersen W, et al. Visceral fat deposition and insulin sensitivity in depressed women with and without comorbid borderline personality disorder. *Psychosom Med*. 2005;67(3):407-412. doi:10.1097/01.psy.0000160458.95955.f4

50.	Kahl KG, Bens S, Ziegler K, et al. Cortisol, the cortisol-dehydroepiandrosterone ratio, and pro-inflammatory cytokines in patients with current major depressive disorder comorbid with borderline personality disorder. *Biol Psychiatry*. 2006;59(7):667-671. doi:10.1016/j.biopsych.2005.08.001

51.	Keeler JL, Patsalos O, Chung R, et al. Reduced MIP-1β as a Trait Marker and Reduced IL-7 and IL-12 as State Markers of Anorexia Nervosa. *J Pers Med*. 2021;11(8):814. doi:10.3390/jpm11080814

52.	Khan A, Leonard D, Defina L, Barlow CE, Willis B, Brown ES. Association between C reactive protein and depression in a population of healthy adults: the Cooper Center Longitudinal Study. *J Investig Med*. 2020;68(5):1019-1023. doi:10.1136/jim-2019-001254

53.	Kim J, Kim JH, Chang KA. Sex Difference in Peripheral Inflammatory Biomarkers in Drug-Naïve Patients with Major Depression in Young Adulthood. *Biomedicines*. 2021;9(7):708. doi:10.3390/biomedicines9070708

54.	Köhler-Forsberg O, Buttenschøn HN, Tansey KE, et al. Association between C-reactive protein (CRP) with depression symptom severity and specific depressive symptoms in major depression. *Brain Behav Immun*. 2017;62:344-350. doi:10.1016/j.bbi.2017.02.020

55.	Ladwig KH, Marten-Mittag B, Löwel H, Döring A, Koenig W. Influence of depressive mood on the association of CRP and obesity in 3205 middle aged healthy men. *Brain Behav Immun*. 2003;17(4):268-275. doi:10.1016/s0889-1591(03)00056-4

56.	Lahlou-Laforet K, Alhenc-Gelas M, Pornin M, et al. Relation of depressive mood to plasminogen activator inhibitor, tissue plasminogen activator, and fibrinogen levels in patients with versus without coronary heart disease. *Am J Cardiol*. 2006;97(9):1287-1291. doi:10.1016/j.amjcard.2005.11.062

57.	Lamers F, Milaneschi Y, Smit JH, Schoevers RA, Wittenberg G, Penninx BWJH. Longitudinal association between depression and inflammatory markers: Results from the Netherlands Study of Depression and Anxiety. *Biol Psychiatry*. 2019;85(10):829-837. doi:10.1016/j.biopsych.2018.12.020

58.	Lavebratt C, Herring MP, Liu JJ, et al. Interleukin-6 and depressive symptom severity in response to physical exercise. *Psychiatry Res*. 2017;252:270-276. doi:10.1016/j.psychres.2017.03.012

59.	Lawes S, Demakakos P, Steptoe A, Lewis G, Carvalho LA. Combined influence of depressive symptoms and systemic inflammation on all-cause and cardiovascular mortality: evidence for differential effects by gender in the English Longitudinal Study of Ageing. *Psychol Med*. 2019;49(9):1521-1531. doi:10.1017/S003329171800209X

60.	Lehtimäki K, Keränen T, Huuhka M, et al. Increase in plasma proinflammatory cytokines after electroconvulsive therapy in patients with depressive disorder. *J ECT*. 2008;24(1):88-91. doi:10.1097/YCT.0b013e3181571abb

61.	Liu Y, Al-Sayegh H, Jabrah R, Wang W, Yan F, Zhang J. Association between C-reactive protein and depression: modulated by gender and mediated by body weight. *Psychiatry Res*. 2014;219(1):103-108. doi:10.1016/j.psychres.2014.05.025

62.	Liu X, Huang J, Jiang Y, et al. IL-6 and IL-8 are likely associated with psychological status in treatment naïve general population. *J Affect Disord*. 2022;298(Pt A):337-344. doi:10.1016/j.jad.2021.10.042

63.	Liukkonen T, Silvennoinen-Kassinen S, Jokelainen J, et al. The association between C-Reactive protein levels and depression: Results from the Northern Finland 1966 Birth Cohort Study. *Biol Psychiatry*. 2006;60(8):825-830. doi:10.1016/j.biopsych.2006.02.016

64.	Liukkonen T, Vanhala M, Jokelainen J, Keinänen-Kiukaanniemi S, Koponen H, Timonen M. Effect of menopause and use of contraceptives/hormone therapy on association of C-reactive protein and depression: a population-based study. *J Psychosom Res*. 2010;68(6):573-579. doi:10.1016/j.jpsychores.2009.11.003

65.	Low CA, Cunningham AL, Kao AH, Krishnaswami S, Kuller LH, Wasko MCM. Association between C-reactive protein and depressive symptoms in women with rheumatoid arthritis. *Biol Psychol*. 2009;81(2):131-134. doi:10.1016/j.biopsycho.2009.02.003

66.	Luukinen H, Jokelainen J, Hedberg P. The relationships between high-sensitivity C-reactive protein and incident depressed mood among older adults. *Scand J Clin Lab Invest*. 2010;70(2):75-79. doi:10.3109/00365510903410548

67.	Ma Y, Chiriboga DE, Pagoto SL, et al. Association between Depression and C-Reactive Protein. *Cardiol Res Pract*. 2010;2011:e286509. doi:10.4061/2011/286509

68.	Marques-Deak AH, Neto FL, Dominguez WV, et al. Cytokine profiles in women with different subtypes of major depressive disorder. *J Psychiatr Res*. 2007;41(1-2):152-159. doi:10.1016/j.jpsychires.2005.11.003

69.	Martin S, Vincent A, Taylor AW, et al. Lower Urinary Tract Symptoms, Depression, Anxiety and Systemic Inflammatory Factors in Men: A Population-Based Cohort Study. *PLOS ONE*. 2015;10(10):e0137903. doi:10.1371/journal.pone.0137903

70.	Matthews KA, Schott LL, Bromberger J, Cyranowski J, Everson-Rose SA, Sowers MF. Associations between depressive symptoms and inflammatory/hemostatic markers in women during the menopausal transition. *Psychosom Med*. 2007;69(2):124-130. doi:10.1097/01.psy.0000256574.30389.1b

71.	Matthews KA, Schott LL, Bromberger JT, Cyranowski JM, Everson-Rose SA, Sowers M. Are there bi-directional associations between depressive symptoms and C-reactive protein in mid-life women? *Brain Behav Immun*. 2010;24(1):96-101. doi:10.1016/j.bbi.2009.08.005

72.	McDade TW, Borja JB, Adair LS, Kuzawa C. Depressive symptoms are not associated with inflammation in younger and older adults in the Philippines. *Evol Med Public Health*. 2013;2013(1):18-23. doi:10.1093/emph/eos004

73.	McLaughlin AP, Nikkheslat N, Hastings C, et al. The influence of comorbid depression and overweight status on peripheral inflammation and cortisol levels. *Psychol Med*. 2022;52(14):3289-3296. doi:10.1017/S0033291721000088

74.	Mehta ND, Stevens JS, Li Z, et al. Inflammation, amygdala-ventromedial prefrontal functional connectivity and symptoms of anxiety and PTSD in African American women recruited from an inner-city hospital: Preliminary results. *Brain Behav Immun*. 2022;105:122-130. doi:10.1016/j.bbi.2022.06.013

75.	Miller GE, Rohleder N, Stetler C, Kirschbaum C. Clinical depression and regulation of the inflammatory response during acute stress. *Psychosom Med*. 2005;67(5):679-687. doi:10.1097/01.psy.0000174172.82428.ce

76.	Mommersteeg PMC, Naudé PJW, Bagijn W, Widdershoven J, Westerhuis BWJJM, Schoemaker RG. Gender differences in associations of depressive symptoms and anxiety with inflammatory markers in patients with non-obstructive coronary artery disease. *J Psychosom Res*. 2019;125:109779. doi:10.1016/j.jpsychores.2019.109779

77.	Morris AA, Zhao L, Ahmed Y, et al. Association between depression and inflammation – Differences by race and sex: The META-Health Study. *Psychosom Med*. 2011;73(6):462-468. doi:10.1097/PSY.0b013e318222379c

78.	Motivala SJ, Sarfatti A, Olmos L, Irwin MR. Inflammatory markers and sleep disturbance in major depression. *Psychosom Med*. 2005;67(2):187-194. doi:10.1097/01.psy.0000149259.72488.09

79.	Naghashpour M, Amani R, Nutr R, Nematpour S, Haghighizadeh MH. Riboflavin status and its association with serum hs-CRP levels among clinical nurses with depression. *J Am Coll Nutr*. 2011;30(5):340-347. doi:10.1080/07315724.2011.10719977

80.	Naicker K, Øverland S, Johnson JA, et al. Symptoms of anxiety and depression in type 2 diabetes: Associations with clinical diabetes measures and self-management outcomes in the Norwegian HUNT study. *Psychoneuroendocrinology*. 2017;84:116-123. doi:10.1016/j.psyneuen.2017.07.002

81.	Ogłodek EA, Szota A, Just MJ, Moś D, Araszkiewicz A. Comparison of chemokines (CCL-5 and SDF-1), chemokine receptors (CCR-5 and CXCR-4) and IL-6 levels in patients with different severities of depression. *Pharmacol Rep*. 2014;66(5):920-926. doi:10.1016/j.pharep.2014.06.001

82.	Ogłodek EA, Just MJ, Szromek AR, Araszkiewicz A. Assessing the serum concentration levels of NT-4/5, GPX-1, TNF-alpha, and l-arginine as biomediators of depression severity in first depressive episode patients with and without posttraumatic stress disorder. *Pharmacol Rep*. 2017;69(5):1049-1058. doi:10.1016/j.pharep.2017.04.013

83.	Ogłodek E. Changes in the Serum Levels of Cytokines: IL-1β, IL-4, IL-8 and IL-10 in Depression with and without Posttraumatic Stress Disorder. *Brain Sci*. 2022;12(3):387. doi:10.3390/brainsci12030387

84.	Olszanecka-Glinianowicz M, Zahorska-Markiewicz B, Kocełak P, et al. Is chronic inflammation a possible cause of obesity-related depression? *Mediators Inflamm*. 2009;2009:439107. doi:10.1155/2009/439107

85.	Osler M, Bruunsgaard H, Lykke Mortensen E. Lifetime socio-economic position and depression: an analysis of the influence of cognitive function, behaviour and inflammatory markers. *Eur J Public Health*. 2015;25(6):1065-1069. doi:10.1093/eurpub/ckv134

86.	Panagiotakos DB, Pitsavos C, Chrysohoou C, et al. Inflammation, coagulation, and depressive symptomatology in cardiovascular disease-free people; the ATTICA study. *Eur Heart J*. 2004;25(6):492-499. doi:10.1016/j.ehj.2004.01.018

87.	Pasco JA, Nicholson GC, Williams LJ, et al. Association of high-sensitivity C-reactive protein with de novo major depression. *Br J Psychiatry*. 2010;197(5):372-377. doi:10.1192/bjp.bp.109.076430

88.	Penninx BWJH, Kritchevsky SB, Yaffe K, et al. Inflammatory markers and depressed mood in older persons: results from the health, aging and body composition study. *Biol Psychiatry*. 2003;54(5):566-572. doi:10.1016/S0006-3223(02)01811-5

89.	Pike JL, Irwin MR. Dissociation of inflammatory markers and natural killer cell activity in major depressive disorder. *Brain Behav Immun*. 2006;20(2):169-174. doi:10.1016/j.bbi.2005.05.004

90.	Poudel-Tandukar K, Bertone-Johnson ER, Palmer PH, Poudel KC. C-reactive protein and depression in persons with Human Immunodeficiency Virus infection: The Positive Living with HIV (POLH) Study. *Brain Behav Immun*. 2014;42:89-95. doi:10.1016/j.bbi.2014.06.004

91.	Powers A, Dixon HD, Conneely K, et al. The differential effects of PTSD, MDD, and dissociation on CRP in trauma-exposed women. *Compr Psychiatry*. 2019;93:33-40. doi:10.1016/j.comppsych.2019.06.007

92.	Prohan M, Amani R, Nematpour S, Jomehzadeh N, Haghighizadeh MH. Total antioxidant capacity of diet and serum, dietary antioxidant vitamins intake, and serum hs-CRP levels in relation to depression scales in university male students. *Redox Rep*. 2014;19(3):133-139. doi:10.1179/1351000214Y.0000000085

93.	Ravona-Springer R, Lutski M, Schnaider Beeri M, Goldbourt U, Tanne D. C-reactive protein in midlife is associated with depressive symptoms two decades later among men with coronary heart disease. *Nord J Psychiatry*. 2020;74(3):226-233. doi:10.1080/08039488.2019.1695284

94.	Redwine LS, Mills PJ, Hong S, et al. Cardiac-related hospitalization and/or death associated with immune dysregulation and symptoms of depression in heart failure patients. *Psychosom Med*. 2007;69(1):23-29. doi:10.1097/PSY.0b013e31802e2f35

95.	Schrock JM, McDade TW, Carrico AW, D’Aquila RT, Mustanski B. Traumatic events and mental health: The amplifying effects of pre-trauma systemic inflammation. *Brain Behav Immun*. 2021;98:173-184. doi:10.1016/j.bbi.2021.08.208

96.	Shimura Y, Kurosawa H, Tsuchiya M, et al. Serum interleukin 6 levels are associated with depressive state of the patients with knee osteoarthritis irrespective of disease severity. *Clin Rheumatol*. 2017;36(12):2781-2787. doi:10.1007/s10067-017-3826-z

97.	Shin D, Shin J, Lee KW. Effects of Inflammation and Depression on Telomere Length in Young Adults in the United States. *J Clin Med*. 2019;8(5):711. doi:10.3390/jcm8050711

98.	Shirom A, Toker S, Melamed S, Berliner S, Shapira I. Vigor, Anxiety, and Depressive Symptoms as Predictors of Changes in Fibrinogen and C-reactive Protein. *Appl Psychol Health Well-Being*. 2010;2(3):251-271. doi:10.1111/j.1758-0854.2010.01033.x

99.	Smagula SF, Ancoli-Israel S, Barrett-Connor E, et al. Inflammation, sleep disturbances, and depressed mood among community-dwelling older men. *J Psychosom Res*. 2014;76(5):368-373. doi:10.1016/j.jpsychores.2014.02.005

100.	Song BM, Lee JM, Choi W, et al. Association between C reactive protein level and depressive symptoms in an elderly Korean population: Korean Social Life, Health and Aging Project. *BMJ Open*. 2015;5(2):e006429. doi:10.1136/bmjopen-2014-006429

101.	Song S, DeMeo NN, Almeida DM, Majd M, Engeland CG, Graham-Engeland JE. The longitudinal connection between depressive symptoms and inflammation: Mediation by sleep quality. *PloS One*. 2022;17(5):e0269033. doi:10.1371/journal.pone.0269033

102.	Sturgeon JA, Darnall BD, Zwickey HL, et al. Proinflammatory cytokines and DHEA-S in women with fibromyalgia: impact of psychological distress and menopausal status. *J Pain Res*. 2014;7:707-716. doi:10.2147/JPR.S71344

103.	Suarez EC. Plasma interleukin-6 is associated with psychological coronary risk factors: Moderation by use of multivitamin supplements. *Brain Behav Immun*. 2003;17(4):296-303. doi:10.1016/S0889-1591(03)00059-X

104.	Surtees PG, Wainwright NWJ, Boekholdt SM, Luben RN, Wareham NJ, Khaw KT. Major depression, C-reactive protein, and incident ischemic heart disease in healthy men and women. *Psychosom Med*. 2008;70(8):850-855. doi:10.1097/PSY.0b013e318183acd5

105.	Tayefi M, Shafiee M, Kazemi-Bajestani SMR, et al. Depression and anxiety both associate with serum level of hs-CRP: A gender-stratified analysis in a population-based study. *Psychoneuroendocrinology*. 2017;81:63-69. doi:10.1016/j.psyneuen.2017.02.035

106.	Toker S, Shirom A, Shapira I, Berliner S, Melamed S. The association between burnout, depression, anxiety, and inflammation biomarkers: C-reactive protein and fibrinogen in men and women. *J Occup Health Psychol*. 2005;10:344-362. doi:10.1037/1076-8998.10.4.344

107.	Torres E, Zumpf KB, Ciolino JD, et al. C-Reactive protein concentrations in reproductive-aged women with major mood disorders. *Arch Womens Ment Health*. 2022;25(3):577-584. doi:10.1007/s00737-022-01222-y

108.	Tsuboi H, Sakakibara H, Minamida Y, et al. Elevated Levels of Serum IL-17A in Community-Dwelling Women with Higher Depressive Symptoms. *Behav Sci*. 2018;8(11):102. doi:10.3390/bs8110102

109.	Tully PJ, Baumeister H, Bengel J, et al. The longitudinal association between inflammation and incident depressive symptoms in men: the effects of hs-CRP are independent of abdominal obesity and metabolic disturbances. *Physiol Behav*. 2015;139:328-335. doi:10.1016/j.physbeh.2014.11.058

110.	Tully PJ, Baumeister H, Martin S, et al. Elucidating the Biological Mechanisms Linking Depressive Symptoms With Type 2 Diabetes in Men: The Longitudinal Effects of Inflammation, Microvascular Dysfunction, and Testosterone. *Psychosom Med*. 2016;78(2):221-232. doi:10.1097/PSY.0000000000000263

111.	Ushiroyama T, Ikeda A, Ueki M. Elevated plasma interleukin-6 (IL-6) and soluble IL-6 receptor concentrations in menopausal women with and without depression. *Int J Gynecol Obstet*. 2002;79(1):51-52. doi:10.1016/S0020-7292(02)00189-3

112.	Vetter ML, Wadden TA, Vinnard C, et al. Gender differences in the relationship between symptoms of depression and high-sensitivity CRP. *Int J Obes*. 2013;37(1):S38-S43. doi:10.1038/ijo.2013.95

113.	Vogelzangs N, Duivis HE, Beekman ATF, et al. Association of depressive disorders, depression characteristics and antidepressant medication with inflammation. *Transl Psychiatry*. 2012;2(2):e79-e79. doi:10.1038/tp.2012.8

114.	Wang S, Zhong H, Lu M, et al. Higher Serum C Reactive Protein Determined C Reactive Protein Single-Nucleotide Polymorphisms Are Involved in Inherited Depression. *Psychiatry Investig*. 2018;15(8):824-828. doi:10.30773/pi.2018.04.03.2

115.	Webb M, Davies M, Ashra N, et al. The association between depressive symptoms and insulin resistance, inflammation and adiposity in men and women. *PLOS ONE*. 2017;12(11):e0187448. doi:10.1371/journal.pone.0187448

116.	Whooley MA, Caska CM, Hendrickson BE, Rourke MA, Ho J, Ali S. Depression and inflammation in patients with coronary heart disease: Findings from the Heart and Soul Study. *Biol Psychiatry*. 2007;62(4):314-320. doi:10.1016/j.biopsych.2006.10.016

117.	Wium-Andersen MK, Orsted DD, Nordestgaard BG. Elevated C-reactive protein, depression, somatic diseases, and all-cause mortality: A mendelian randomization study. *Biol Psychiatry*. 2014;76(3):249-257. doi:10.1016/j.biopsych.2013.10.009

118.	Wysokiński A, Socha K, Sołtysik BK, Kłoszewska I, Sobów T, Kostka T. Levels of C-reactive protein (CRP) in elderly patients with unipolar depression – case control analysis. *Nord J Psychiatry*. 2016;70(7):503-507. doi:10.3109/08039488.2016.1174298

119.	Yang K, Xie G, Zhang Z, et al. Levels of serum interleukin (IL)-6, IL-1β, tumour necrosis factor-α and leptin and their correlation in depression. *Aust N Z J Psychiatry*. 2007;41(3):266-273. doi:10.1080/00048670601057759

120.	Yang QQ, Shao D, Li J, Yang CL, Fan MH, Cao FL. Positive association between serum levels of high-sensitivity C-reactive protein and depression/anxiety in female, but not male, patients with type 2 diabetes mellitus. *Biol Res Nurs*. 2020;22(2):178-187. doi:10.1177/1099800419894641

121.	Ye Z, Kappelmann N, Moser S, et al. Role of inflammation in depression and anxiety: Tests for disorder specificity, linearity and potential causality of association in the UK Biobank. *EClinicalMedicine*. 2021;38:100992. doi:10.1016/j.eclinm.2021.100992

122.	Zahra A, Obaia E, Hassouna A, Ezat M. The Relation between Major Depression and Plasma levels of Cytokines and High Sensitivity C-Reactive Protein. *Bull Egypt Soc Physiol Sci*. 2007;27(2):307-320. doi:10.21608/besps.2007.37406

123.	Zhang L, Li JL, Zhang LL, Guo LL, Li H, Li D. No association between C-reactive protein and depressive symptoms among the middle-aged and elderly in China: Evidence from the China Health and Retirement Longitudinal Study. *Medicine (Baltimore)*. 2018;97(38):e12352. doi:10.1097/MD.0000000000012352

124.	Zheng H, Jin X, Zheng X. The relationship between the joint effect of C-reactive protein and glycated hemoglobin with the risk of depressive symptoms among middle-aged and older adults: Findings from the China health and retirement longitudinal study. *J Psychiatr Res*. 2023;158:88-94. doi:10.1016/j.jpsychires.2022.12.046

## Table S5-S19: Sensitivity Analyses: Meta-analytic and meta-regression results

#### Table S5. Sensitivity analyses: meta-analytic results for cross-sectional studies: depression-biomarker associations separately among females and males

|  |  |  |  |  | Unmeasured Confounding | | | Publication Bias | |
| --- | --- | --- | --- | --- | --- | --- | --- | --- | --- |
|  | k_pap._ | k_est._ | n_obs._ | Cohen’s *d* [95% CI] | E-val_est_ | E-val_LCI_ | Ghat | Selection Model Ratio [95% CI] | Worst-Case Cohen’s *d* [95% CI] |
| Females |  |  |  |  |  |  |  |  |  |
| All Markers | **85** | **147** | **211,926** | **0.06 [0.03, 0.10]** | 1.31 | 1.19 | -- | **0.91 [0.59, 1.23]** | **0.03 [0.00, 0.06]** |
| CRP | 60 | 69 | 190,865 | 0.01 [-0.01, 0.03] | 1.12 | 1.00 | -- | -- | 0.01 [-0.01, 0.03] |
| IL-6 | **40** | **41** | **11,565** | **0.09 [0.02, 0.17]** | 1.40 | 1.17 | 1.46 | -- | 0.03 [-0.08, 0.13] |
| TNF-α | 22 | 23 | 5,550 | 0.15 [-0.04, 0.35] | 1.56 | 1.00 | 1.62 | -- | 0.07 [-0.04, 0.17] |
| IL-1β | 11 | 11 | 2,708 | 0.41 [-0.36, 1.18] | 2.27 | 1.00 | 2.77 | -- | -0.08 [-0.20, 0.04] |
| Fibrinogen | 3 | 3 | 1,238 | 0.23 [-0.76, 1.22] | 1.77 | 1.00 | -- | -- | 0.00 [-0.77, 0.77] |
|  |  |  |  |  |  |  |  |  |  |
| Males |  |  |  |  |  |  |  |  |  |
| All Markers | **71** | **108** | **206,007** | **0.14 [0.09, 0.19]** | 1.53 | 1.39 | 1.46 | **2.25 [1.66, 2.85]** | **0.08 [0.02, 0.13]** |
| CRP | **55** | **58** | **176,475** | **0.08 [0.05, 0.11]** | 1.36 | 1.28 | 1.00 | **0.60 [0.31, 0.88]** | **0.09 [0.04, 0.13]** |
| IL-6 | **27** | **27** | **18,272** | **0.13 [0.04, 0.22]** | 1.50 | 1.25 | 1.79 | -- | 0.04 [-0.07, 0.15] |
| TNF-α | 11 | 11 | 6,083 | 0.30 [-0.37, 0.98] | 1.97 | 1.00 | 1.97 | -- | 0.05 [-0.12, 0.22] |
| IL-1β | 6 | 6 | 1,905 | 1.84 [-1.10, 4.78] | 10.14 | 1.00 | -- | -- | 0.11 [-0.25, 0.48] |
| Fibrinogen | 6 | 6 | 3,272 | 0.27 [-0.21, 0.75] | 1.88 | 1.00 | -- | -- | -0.01 [-0.48, 0.47] |

***Note.*** Estimates from the primary models are repeated for context. k_pap._ = number of papers contributing to each model; k_est._. = number of total estimates (some papers contributed more than one estimate to each model); n_obs._ = total sample size (number of individual observations in each model). E-val = minimum strength of association on risk ratio scale an unmeasured confounder would need with both exposure and outcome, conditional on measured covariates to explain away the observed association (to shift the estimate [E-val_est_] or the confidence interval [E-val_LCI_] to the null). Ghat = minimum strength of unmeasured confounding on risk ratio scale required to reduce the proportion of true effects stronger than to below 10%; only calculated in models where k>10, τ^2^>0, and the proportion of true effects larger than +/-0.2 was >10%. Selection models estimate the ratio of publishing affirmative to non-affirmative results; only calculated in models where k>30, and ≥5 affirmative and non-affirmative estimates. Worst-case Cohen’s *d* are calculated among only non-affirmative estimates. Results in bold indicate *p* <0.05 and italics indicate 0.05≤*p<0.10*.

#### Table S6. Sensitivity analyses: meta-analytic results for longitudinal studies: depression-biomarker associations separately among females and males

|  |  |  |  |  | Unmeasured Confounding | | Publication Bias |
| --- | --- | --- | --- | --- | --- | --- | --- |
|  | k_pap._ | k_est._ | n_obs._ | Cohen’s *d* [95% CI] | E-val_est_ | E-val_LCI_ | Worst-Case Cohen’s *d* [95% CI] |
| Depression and subsequent Inflammation | | | | | | |  |
| Females |  |  |  |  |  |  |  |
| All Markers | 6 | 9 | 14,083 | 0.08 [-0.14, 0.31] | 1.37 | 1.00 | 0.00 [-0.03, 0.02] |
| CRP | 6 | 6 | 12,492 | 0.07 [-0.12, 0.27] | 1.34 | 1.00 | 0.00 [-0.02, 0.01] |
| IL-6 | 2 | 2 | 1,388 | 0.32 [-3.35, 3.99] | 2.01 | 1.00 | 0.03 [-0.03, 0.10] |
|  |  |  |  |  |  |  |  |
| Males |  |  |  |  |  |  |  |
| All Markers | 7 | 15 | 10,602 | 0.04 [-0.02, 0.11] | 1.25 | 1.00 | -0.01 [-0.05, 0.04] |
| CRP | 7 | 7 | 5,006 | 0.10 [-0.03, 0.22] | 1.41 | 1.00 | 0.00 [-0.06, 0.07] |
| IL-6 | 5 | 5 | 4,022 | 0.01 [-0.08, 0.11] | 1.12 | 1.00 | -0.01 [-0.12, 0.10] |
| Inflammation and subsequent Depression | | | | | | | |
| Females |  |  |  |  |  |  |  |
| All Markers | **12** | **17** | **29,624** | **0.03 [0.00, 0.06]** | 1.20 | 1.09 | *0.02 [0.00, 0.04]* |
| CRP | 11 | 11 | 24,702 | 0.02 [-0.01, 0.04] | 1.14 | 1.00 | 0.01 [-0.04, 0.06] |
| IL-6 | **5** | **5** | **4,824** | **0.05 [0.00, 0.10]** | 1.27 | 1.18 | 0.04 [-0.02, 0.10] |
|  |  |  |  |  |  |  |  |
| Males |  |  |  |  |  |  |  |
| All Markers | *11* | *18* | *19,830* | *0.07 [-0.01, 0.15]* | 1.33 | 1.00 | 0.00 [-0.05, 0.05] |
| CRP | **10** | **10** | **14,043** | **0.06 [0.00, 0.13]** | 1.31 | 1.13 | 0.03 [-0.03, 0.10] |
| IL-6 | 6 | 6 | 4,568 | 0.03 [-0.05, 0.10] | 1.18 | 1.00 | -0.01 [-0.06, 0.03] |

***Note.*** Estimates from the primary models are repeated for context. k_pap._ = number of papers contributing to each model; k_est._. = number of total estimates (some papers contributed more than one estimate to each model); n_obs._ = total sample size (number of individual observations in each model). E-val = minimum strength of association on risk ratio scale an unmeasured confounder would need with both exposure and outcome, conditional on measured covariates to explain away the observed association (to shift the estimate [E-val_est_] or the confidence interval [E-val_LCI_] to the null). Worst-case Cohen’s *d* are calculated among only non-affirmative estimates. Results in bold indicate *p* <0.05 and italics indicate 0.05≤*p<0.10*.

#### Table S7. Meta-regression results for effects of level of confounding adjustment in cross-sectional studies: depression-biomarker associations

|  | k_pap._ | k_est._ | n_obs._ | Predictor | Cohen’s *d* [95% CI] | p | τ^2^ | I^2^ | df |
| --- | --- | --- | --- | --- | --- | --- | --- | --- | --- |
| All Markers – Females | 86 | 181 | 375,942 | Unadjusted | **0.17 [0.09, 0.25]** | **<.001** | 0.01 | 89.18 | 35.40 |
|  |  |  |  | Minimally adjusted | **-0.16 [-0.31, -0.02]** | **0.028** |  |  | 6.10 |
|  |  |  |  | Fully adjusted | **-0.14 [-0.22, -0.06]** | **0.001** |  |  | 53.08 |
|  |  |  |  |  |  |  |  |  |  |
| All Markers - Males | 73 | 137 | 365,123 | Unadjusted | **0.27 [0.15, 0.40]** | **<.001** | 0.02 | 92.15 | 32.27 |
|  |  |  |  | Minimally adjusted | **-0.23 [-0.40, -0.06]** | **0.017** |  |  | 5.21 |
|  |  |  |  | Fully adjusted | **-0.17 [-0.30, -0.04]** | **0.011** |  |  | 50.46 |
|  |  |  |  |  |  |  |  |  |  |
| CRP - Females | 61 | 91 | 349,710 | Unadjusted | 0.06 [-0.02, 0.13] | 0.104 | 0.00 | 82.98 | 3.96 |
|  |  |  |  | Minimally adjusted | -0.05 [-0.18, 0.09] | 0.356 |  |  | 3.23 |
|  |  |  |  | Fully adjusted | *-0.05 [-0.11, 0.01]* | *0.082* |  |  | 6.07 |
|  |  |  |  |  |  |  |  |  |  |
| CRP - Males | 57 | 79 | 329,143 | Unadjusted | **0.17 [0.10, 0.25]** | **<.001** | 0.01 | 83.57 | 18.48 |
|  |  |  |  | Minimally adjusted | **-0.13 [-0.24, -0.03]** | **0.021** |  |  | 5.54 |
|  |  |  |  | Fully adjusted | **-0.10 [-0.18, -0.02]** | **0.012** |  |  | 31.18 |
|  |  |  |  |  |  |  |  |  |  |
| IL-6- Females | 40 | 49 | 14,722 | Unadjusted | **0.17 [0.02, 0.32]** | **0.025** | 0.05 | 86.57 | 21.15 |
|  |  |  |  | Minimally adjusted | *-0.19 [-0.41, 0.03]* | *0.073* |  |  | 4.37 |
|  |  |  |  | Fully adjusted | -0.10 [-0.26, 0.06] | 0.224 |  |  | 24.38 |
|  |  |  |  |  |  |  |  |  |  |
| IL-6 - Males | 27 | 33 | 23,225 | Unadjusted | **0.29 [0.06, 0.52]** | **0.019** | 0.05 | 91.00 | 10.84 |
|  |  |  |  | Minimally adjusted | *-0.29 [-0.61, 0.03]* | *0.065* |  |  | 3.97 |
|  |  |  |  | Fully adjusted | -0.20 [-0.44, 0.05] | 0.106 |  |  | 17.86 |
|  |  |  |  |  |  |  |  |  |  |
| TNF-α - Females | 22 | 26 | 6,934 | Unadjusted | 0.40 [-0.12, 0.92] | 0.119 | 0.13 | 91.18 | 14.31 |
|  |  |  |  | Minimally adjusted | -0.47 [-2.15, 1.22] | 0.260 |  |  | 1.42 |
|  |  |  |  | Fully adjusted | -0.35 [-0.93, 0.23] | 0.198 |  |  | 7.90 |
|  |  |  |  |  |  |  |  |  |  |
| TNF-α - Males | 11 | 12 | 6,645 | Unadjusted | 0.79 [-1.14, 2.72] | 0.351 | 0.32 | 95.59 | 5.50 |
|  |  |  |  | Minimally adjusted | -0.79 [-2.72, 1.15] | 0.351 |  |  | 5.50 |
|  |  |  |  | Fully adjusted | -0.79 [-2.68, 1.10] | 0.347 |  |  | 6.03 |
|  |  |  |  |  |  |  |  |  |  |
| IL-1β - Females | 11 | 11 | 2,708 | Unadjusted | 0.58 [-0.51, 1.67] | 0.252 | 0.91 | 94.21 | 7.92 |
|  |  |  |  | Minimally adjusted | -0.69 [-1.78, 0.40] | 0.181 |  |  | 7.92 |
|  |  |  |  | Fully adjusted | -0.51 [-1.60, 0.58] | 0.310 |  |  | 7.92 |
|  |  |  |  |  |  |  |  |  |  |
| IL-1β - Males | 6 | 6 | 1,905 | Unadjusted | 2.99 [-2.21, 8.19] | 0.165 | 9.39 | 97.81 | 3.00 |
|  |  |  |  | Minimally adjusted | -2.97 [-8.17, 2.22] | 0.166 |  |  | 3.00 |
|  |  |  |  | Fully adjusted | -2.94 [-8.14, 2.25] | 0.169 |  |  | 3.00 |

***Note.*** Estimates reflect dummy coding where the coefficient for Unadjusted refers to the average effect with no covariates, and the coefficients for Minimally adjusted and Adjusted refer to the average difference in effect when adjusted for at demographics only and for demographics and at least one other domain of confounder (e.g., health behaviors, BMI, medication, illness), respectively. k_pap._ = number of papers contributing to each model; k_est._. = number of total estimates (some papers contributed more than one estimate to each model); n_obs._ = total sample size (number of individual observations in each model). Results in bold indicate *p* <0.05 and italics indicate 0.05≤*p<0.10*.

#### Table S8. Meta-regression results for effects of level of confounding adjustment in longitudinal studies: depression-biomarker associations

|  | k_pap._ | k_est._ | n_obs._ | Predictor | Cohen’s *d* [95% CI] | p | τ^2^ | I^2^ | df |
| --- | --- | --- | --- | --- | --- | --- | --- | --- | --- |
| Depression and subsequent Inflammation | | | | | | | | |  |
| All Markers - Females | 6 | 15 | 26,096 | Unadjusted | 0.15 [-0.25, 0.55] | 0.322 | 0.02 | 95.96 | 2.97 |
|  |  |  |  | Minimally adjusted | -0.13 [-0.53, 0.27] | 0.364 |  |  | 2.97 |
|  |  |  |  | Fully adjusted | -0.07 [-0.23, 0.10] | 0.316 |  |  | 3.77 |
|  |  |  |  |  |  |  |  |  |  |
| All Markers - Males | 7 | 19 | 12,564 | Unadjusted | 0.08 [-0.06, 0.22] | 0.130 | 0.01 | 81.93 | 1.78 |
|  |  |  |  | Minimally adjusted | -0.09 [-0.23, 0.05] | 0.107 |  |  | 1.78 |
|  |  |  |  | Fully adjusted | -0.03 [-0.16, 0.10] | 0.576 |  |  | 3.34 |
|  |  |  |  |  |  |  |  |  |  |
| CRP - Females | 6 | 10 | 23,777 | Unadjusted | 0.14 [-0.21, 0.48] | 0.287 | 0.01 | 95.10 | 2.95 |
|  |  |  |  | Minimally adjusted | -0.14 [-0.49, 0.20] | 0.276 |  |  | 2.95 |
|  |  |  |  | Fully adjusted | -0.07 [-0.21, 0.08] | 0.280 |  |  | 3.74 |
|  |  |  |  |  |  |  |  |  |  |
| CRP - Males | 7 | 9 | 5,987 | Unadjusted | 0.13 [-0.10, 0.36] | 0.126 | 0.01 | 84.36 | 1.78 |
|  |  |  |  | Minimally adjusted | -0.15 [-0.38, 0.08] | 0.105 |  |  | 1.78 |
|  |  |  |  | Fully adjusted | -0.01 [-0.22, 0.20] | 0.890 |  |  | 3.34 |
|  |  |  |  |  |  |  |  |  |  |
| IL-6 - Males | 5 | 6 | 4,465 | Unadjusted | 0.04 [-1.06, 1.14] | 0.739 | 0.01 | 74.77 | 1.00 |
|  |  |  |  | Minimally adjusted | -0.04 [-1.14, 1.06] | 0.709 |  |  | 1.00 |
|  |  |  |  | Fully adjusted | -0.03 [-0.35, 0.29] | 0.738 |  |  | 2.12 |
|  |  |  |  |  |  |  |  |  |  |
| Inflammation and subsequent Depression | | | | | | | | |  |
| All Markers - Females | 12 | 23 | 46,214 | Unadjusted | 0.03 [-0.13, 0.19] | 0.273 | 0.00 | 50.95 | 1.00 |
|  |  |  |  | Minimally adjusted | 0.02 [-0.08, 0.13] | 0.369 |  |  | 1.55 |
|  |  |  |  | Fully adjusted | 0.00 [-0.17, 0.18] | 0.862 |  |  | 1.29 |
|  |  |  |  | Unadjusted |  |  |  |  |  |
| All Markers - Males | 13 | 25 | 24,014 | Minimally adjusted | 0.12 [-0.07, 0.31] | 0.102 | 0.01 | 61.12 | 1.69 |
|  |  |  |  | Fully adjusted | -0.07 [-0.20, 0.06] | 0.160 |  |  | 2.70 |
|  |  |  |  | Unadjusted | -0.06 [-0.21, 0.08] | 0.277 |  |  | 3.20 |
|  |  |  |  | Minimally adjusted |  |  |  |  |  |
| CRP - Females | 11 | 15 | 37,920 | Fully adjusted | 0.03 [-0.12, 0.18] | 0.262 | 0.00 | 49.06 | 1.00 |
|  |  |  |  | Unadjusted | 0.01 [-0.14, 0.15] | 0.847 |  |  | 1.51 |
|  |  |  |  | Minimally adjusted | -0.01 [-0.14, 0.12] | 0.626 |  |  | 1.33 |
|  |  |  |  | Fully adjusted |  |  |  |  |  |
| CRP - Males | 11 | 14 | 16,179 | Unadjusted | 0.16 [-0.33, 0.65] | 0.157 | 0.00 | 46.71 | 1.03 |
|  |  |  |  | Minimally adjusted | -0.10 [-0.30, 0.09] | 0.146 |  |  | 1.85 |
|  |  |  |  | Fully adjusted | -0.11 [-0.35, 0.13] | 0.168 |  |  | 1.68 |

***Note.*** Estimates reflect dummy coding where the coefficient for Unadjusted refers to the average effect with no covariates, and the coefficients for Minim adjusted and Adjusted refer to the average difference in effect when adjusted for at demographics only and for demographics and at least one other domain of confounder (e.g., health behaviors, BMI, medication, illness), respectively. k_pap._ = number of papers contributing to each model; k_est._. = number of total estimates (some papers contributed more than one estimate to each model); n_obs._ = total sample size (number of individual observations in each model). Results in bold indicate *p* <0.05 and italics indicate 0.05≤*p<0.10*.

#### Table S9. Meta-analytic results for cross-sectional studies: depression-biomarker associations separately among females and males, restricted to unadjusted estimates

|  | k_pap._ | k_est._ | n_obs._ | Cohen’s *d* [95% CI] | p | p_Bon._ | τ^2^ | df | % positive | % positive and *d*>.20 | % negative and *d*<-.20 |
| --- | --- | --- | --- | --- | --- | --- | --- | --- | --- | --- | --- |
| Females |  |  |  |  |  |  |  |  |  |  |  |
| All Markers | 60 | 103 | 129,663 | **0.19 [0.10, 0.28]** | **<.001** | **<.001** | 0.17 | 43.63 | 89 [77, 100] | 30 [0, 47] | 0 |
| CRP | 39 | 46 | 121,322 | **0.10 [0.06, 0.15]** | **<.001** | **0.001** | 0.08 | 20.80 | 93 [70, 98] | 22 [0, 54] | 0 |
| IL-6 | 28 | 29 | 4,276 | **0.19 [0.04, 0.34]** | **0.015** | 0.216 | 0.34 | 23.45 | 72 [39, 86] | 38 [10, 55] | 3 [0, 14] |
| TNF-α | 17 | 18 | 2,663 | 0.43 [-0.13, 0.98] | 0.121 | 1.000 | 0.47 | 15.14 | 83 [0, 94] | 50 [0, 72] | 0 |
| IL-1β | 9 | 9 | 772 | 0.58 [-0.51, 1.67] | 0.252 | 1.000 | 0.95 | 7.92 | 100 | 100 | 0 |
|  |  |  |  |  |  |  |  |  |  |  |  |
| Males |  |  |  |  |  |  |  |  |  |  |  |
| All Markers | 50 | 69 | 115,595 | **0.31 [0.17, 0.45]** | **<.001** | **<.001** | 0.20 | 38.12 | 93 [80, 97] | 45 [29, 61] | 0 |
| CRP | 35 | 37 | 105,818 | **0.19 [0.11, 0.27]** | **<.001** | **<.001** | 0.12 | 23.79 | 97 [84, 100] | 41 [8, 59] | 0 |
| IL-6 | 17 | 17 | 6,286 | **0.31 [0.06, 0.55]** | **0.018** | 0.246 | 0.27 | 12.01 | 94 [71, 100] | 47 [0, 71] | 0 |
| TNF-α | 7 | 7 | 1,172 | 0.88 [-1.21, 2.97] | 0.340 | 1.000 | 0.77 | 5.83 | 100 | 100 | 0 |
| IL-1β | 4 | 4 | 213 | 2.99 [-2.21, 8.19] | 0.165 | 1.000 | 3.06 | 3.00 | 100 | 100 | 0 |
| Fibrinogen | 4 | 4 | 2,106 | 0.14 [-0.33, 0.61] | 0.332 | 1.000 | 0.14 | 2.13 | 100 | 0 | 0 |

***Note.*** k_pap._ = number of papers contributing to each model; k_est._. = number of total estimates (some papers contributed more than one estimate to each model); n_obs._ = total sample size (number of individual observations in each model). *p_Bon_****.*** *=* Bonferroni adjusted p value. %’s refer to the estimated percentage of population effects. Results in bold indicate *p* <0.05 and italics indicate 0.05≤*p<0.10*.

#### Table S10. Meta-analytic results for longitudinal studies: depression-biomarker associations separately among females and males, restricted to unadjusted estimates

|  | k_pap._ | k_est._ | n_obs._ | Cohen’s *d* [95% CI] | p | p_Bon._ | τ^2^ | df | % positive | % positive and *d*>.20 | % negative and *d*<-.20 |
| --- | --- | --- | --- | --- | --- | --- | --- | --- | --- | --- | --- |
| Depression and subsequent Inflammation | | | | | | | | | | | |
| Females |  |  |  |  |  |  |  |  |  |  |  |
| All Markers | 4 | 6 | 12,013 | 0.15 [-0.25, 0.55] | 0.322 | 1.000 | 0.13 | 2.97 | 100 | 0 | 0 |
| CRP | 4 | 4 | 11,285 | 0.14 [-0.21, 0.48] | 0.286 | 1.000 | 0.12 | 2.95 | 100 | 0 | 0 |
|  |  |  |  |  |  |  |  |  |  |  |  |
| Males |  |  |  |  |  |  |  |  |  |  |  |
| All Markers | 3 | 6 | 3,338 | 0.07 [-0.06, 0.20] | 0.150 | 1.000 | 0.12 | 2.00 | 100 | 0 | 0 |
| CRP | 3 | 3 | 1,669 | 0.13 [-0.15, 0.42] | 0.179 | 1.000 | 0.10 | 2.00 | 100 | 0 | 0 |
| IL-6 | 2 | 2 | 1,131 | 0.01 [-1.18, 1.19] | 0.964 | 1.000 | 0.12 | 1.00 | 100 | 0 | 0 |
| Inflammation and subsequent Depression | | | | | | | | | | | |
| Females |  |  |  |  |  |  |  |  |  |  |  |
| All Markers | 2 | 2 | 7,725 | *0.03 [-0.02, 0.08]* | *0.088* | 0.615 | 0.00 | 1.00 | 100 | 0 | 0 |
| CRP | 2 | 2 | 7,725 | *0.03 [-0.02, 0.08]* | *0.088* | 0.527 | 0.00 | 1.00 | 100 | 0 | 0 |
|  |  |  |  |  |  |  |  |  |  |  |  |
| Males |  |  |  |  |  |  |  |  |  |  |  |
| All Markers | 4 | 5 | 2,507 | *0.15 [-0.06, 0.36]* | *0.093* | 0.650 | 0.11 | 2.15 | 100 | 0 | 0 |
| CRP | 3 | 3 | 1,158 | 0.19 [-0.36, 0.75] | 0.161 | 0.969 | 0.08 | 1.15 | 100 | 0 | 0 |

***Note.*** k_pap._ = number of papers contributing to each model; k_est._. = number of total estimates (some papers contributed more than one estimate to each model); n_obs._ = total sample size (number of individual observations in each model). *p_Bon_****.*** *=* Bonferroni adjusted p value. %’s refer to the estimated percentage of population effects. Results in bold indicate *p* <0.05 and italics indicate 0.05≤*p<0.10*.

#### Table S11. Meta-analytic results for cross-sectional studies: depression-biomarker associations separately among females and males, restricted to fully adjusted estimates

|  | k_pap._ | k_est._ | n_obs._ | Cohen’s *d* [95% CI] | p | p_Bon._ | τ^2^ | df | % positive | % positive and *d*>.20 | % negative and *d*<-.20 |
| --- | --- | --- | --- | --- | --- | --- | --- | --- | --- | --- | --- |
| Females |  |  |  |  |  |  |  |  |  |  |  |
| All Markers | 39 | 60 | 179,968 | 0.02 [0.00, 0.04] | 0.062 | 0.249 | 0.03 | 16.82 | 95 [80, 100] | 0 | 0 |
| CRP | 33 | 37 | 171,397 | 0.01 [-0.01, 0.03] | 0.299 | 1.000 | 0.00 | 3.15 | 92 [73, 100] | 0 | 0 |
| IL-6 | 14 | 15 | 5,194 | **0.06 [0.02, 0.10]** | **0.013** | 0.203 | 0.03 | 5.78 | 93 [47, 100] | 0 | 0 |
| TNF-α | 6 | 6 | 2,233 | 0.02 [-0.04, 0.08] | 0.243 | 1.000 | 0.02 | 1.89 | 100 | 0 | 0 |
| Fibrinogen | 3 | 3 | 1,238 | 0.23 [-0.76, 1.22] | 0.419 | 1.000 | 0.47 | 2.00 | 100 | 100 | 0 |
|  |  |  |  |  |  |  |  |  |  |  |  |
| Males |  |  |  |  |  |  |  |  |  |  |  |
| All Markers | 37 | 54 | 174,733 | **0.05 [0.03, 0.08]** | **<.001** | **0.001** | 0.04 | 17.37 | 93 [75, 98] | 2 [0, 20] | 0 |
| CRP | 33 | 35 | 159,140 | **0.04 [0.02, 0.06]** | **<.001** | **0.006** | 0.02 | 11.11 | 100 [80, 100] | 0 | 0 |
| IL-6 | 11 | 11 | 9,609 | 0.05 [-0.03, 0.13] | 0.189 | 1.000 | 0.07 | 7.66 | 82 [0, 91] | 9 [0, 49] | 0 |
| TNF-α | 4 | 4 | 3,833 | 0.00 [-0.06, 0.06] | 0.973 | 1.000 | 0.00 | 1.94 | 0 | 0 | 0 |
| Fibrinogen | 3 | 3 | 2,099 | 0.22 [-0.97, 1.42] | 0.506 | 1.000 | 0.42 | 2.00 | 100 | 100 | 0 |

***Note.*** k_pap._ = number of papers contributing to each model; k_est._. = number of total estimates (some papers contributed more than one estimate to each model); n_obs._ = total sample size (number of individual observations in each model). *p_Bon_****.*** *=* Bonferroni adjusted p value. %’s refer to the estimated percentage of population effects. Results in bold indicate *p* <0.05 and italics indicate 0.05≤*p<0.10*.

#### Table S12. Meta-analytic results for longitudinal studies: depression-biomarker associations separately among females and males, restricted to fully adjusted estimates

|  | k_pap._ | k_est._ | n_obs._ | Cohen’s *d* [95% CI] | p | p_Bon._ | τ^2^ | df | % positive | % positive and *d*>.20 | % negative and *d*<-.20 |
| --- | --- | --- | --- | --- | --- | --- | --- | --- | --- | --- | --- |
| Depression and subsequent Inflammation | | | | | | | | | | | |
| Females |  |  |  |  |  |  |  |  |  |  |  |
| All Markers | 5 | 7 | 12,262 | 0.10 [-0.20, 0.40] | 0.408 | 1.000 | 0.13 | 3.96 | 100 | 0 | 0 |
| CRP | 5 | 5 | 11,534 | 0.09 [-0.17, 0.35] | 0.387 | 1.000 | 0.12 | 3.94 | 100 | 0 | 0 |
|  |  |  |  |  |  |  |  |  |  |  |  |
| Males |  |  |  |  |  |  |  |  |  |  |  |
| All Markers | 5 | 11 | 4,624 | 0.05 [-0.05, 0.15] | 0.260 | 1.000 | 0.12 | 3.96 | 64 [0, 91] | 18 [0, 36] | 0 |
| CRP | 5 | 5 | 1,923 | 0.12 [-0.08, 0.32] | 0.167 | 1.000 | 0.14 | 3.97 | 100 | 0 | 0 |
| IL-6 | 3 | 3 | 1,127 | -0.01 [-0.24, 0.22] | 0.828 | 1.000 | 0.07 | 1.90 | 0 | 0 | 0 |
| Inflammation and subsequent Depression | | | | | | | | | | | |
| Females |  |  |  |  |  |  |  |  |  |  |  |
| All Markers | 10 | 13 | 26,367 | 0.03 [-0.01, 0.07] | 0.088 | 0.703 | 0.04 | 5.03 | 100 [69, 77] | 0 | 0 |
| CRP | 8 | 8 | 22,396 | 0.02 [-0.02, 0.05] | 0.194 | 1.000 | 0.03 | 3.55 | 100 | 0 | 0 |
| IL-6 | 4 | 4 | 3,873 | *0.06 [-0.01, 0.12]* | *0.059* | 0.534 | 0.00 | 1.69 | 100 | 0 | 0 |
|  |  |  |  |  |  |  |  |  |  |  |  |
| Males |  |  |  |  |  |  |  |  |  |  |  |
| All Markers | 10 | 15 | 14,396 | 0.07 [-0.03, 0.18] | 0.138 | 1.000 | 0.11 | 7.21 | 73 [0, 93] | 7 [0, 55] | 0 |
| CRP | 8 | 8 | 10,956 | 0.09 [-0.04, 0.21] | 0.135 | 1.000 | 0.08 | 4.72 | 100 | 0 | 0 |
| IL-6 | 5 | 5 | 2,221 | 0.02 [-0.09, 0.13] | 0.662 | 1.000 | 0.06 | 3.18 | 100 | 0 | 0 |

***Note.*** k_pap._ = number of papers contributing to each model; k_est._. = number of total estimates (some papers contributed more than one estimate to each model); n_obs._ = total sample size (number of individual observations in each model). *p_Bon_****.*** *=* Bonferroni adjusted p value. %’s refer to the estimated percentage of population effects. Results in bold indicate *p* <0.05 and italics indicate 0.05≤*p<0.10*.

#### Table S13. Meta-analytic results for cross-sectional studies: depression-biomarker associations separately among females and males, restricted to medical illness samples

|  | k_pap._ | k_est._ | n_obs._ | Cohen’s *d* [95% CI] | p | p_Bon._ | τ^2^ | df | % positive | % positive and *d*>.20 | % negative and *d*<-.20 |
| --- | --- | --- | --- | --- | --- | --- | --- | --- | --- | --- | --- |
| Females |  |  |  |  |  |  |  |  |  |  |  |
| All Markers | 17 | 29 | 5,152 | **0.06 [0.01, 0.10]** | **0.020** | 0.161 | 0.00 | 7.12 | 100 | 0 | 0 |
| CRP | 13 | 13 | 3,283 | 0.03 [-0.04, 0.10] | 0.293 | 1.000 | 0.03 | 3.32 | 100 [69, 100] | 0 | 0 |
| IL-6 | 9 | 10 | 1,143 | **0.10 [0.03, 0.17]** | **0.027** | 0.746 | 0.00 | 2.17 | 100 | 0 | 0 |
| TNF-α | 4 | 5 | 543 | 0.00 [-0.23, 0.23] | 0.901 | 1.000 | 0.00 | 1.21 | 100 | 0 | 0 |
|  |  |  |  |  |  |  |  |  |  |  |  |
| Males |  |  |  |  |  |  |  |  |  |  |  |
| All Markers | 10 | 14 | 7,318 | 0.07 [-0.03, 0.17] | 0.126 | 1.000 | 0.10 | 6.44 | 64 [14, 86] | 7 [0, 29] | 0 |
| CRP | 9 | 9 | 3,920 | **0.08 [0.00, 0.17]** | **0.047** | 1.000 | 0.08 | 5.82 | 100 | 0 | 0 |
| IL-6 | 3 | 3 | 1,708 | -0.05 [-1.09, 1.00] | 0.780 | 1.000 | 0.18 | 1.27 | 0 | 0 | 0 |
| Fibrinogen | 2 | 2 | 1,690 | -0.07 [-1.01, 0.88] | 0.543 | 1.000 | 0.09 | 1.00 | 0 | 0 | 0 |

***Note.*** k_pap._ = number of papers contributing to each model; k_est._. = number of total estimates (some papers contributed more than one estimate to each model); n_obs._ = total sample size (number of individual observations in each model). *p_Bon_****.*** *=* Bonferroni adjusted p value. %’s refer to the estimated percentage of population effects. Results in bold indicate *p* <0.05 and italics indicate 0.05≤*p<0.10*.

*[note: there were not enough samples to conduct meta-analyses for longitudinal studies with medical illness samples]*

#### Table S14. Meta-analytic results for cross-sectional studies: depression-biomarker associations separately among females and males, restricted to non-medical illness samples

|  | k_pap._ | k_est._ | n_obs._ | Cohen’s *d* [95% CI] | p | p_Bon._ | τ^2^ | df | % positive | % positive and *d*>.20 | % negative and *d*<-.20 |
| --- | --- | --- | --- | --- | --- | --- | --- | --- | --- | --- | --- |
| Females |  |  |  |  |  |  |  |  |  |  |  |
| All Markers | 69 | 118 | 206,774 | **0.06 [0.02, 0.11]** | **0.007** | *0.053* | 0.08 | 37.64 | 81 [68, 95] | 6 [0, 26] | 0 |
| CRP | 48 | 56 | 187,582 | 0.02 [-0.01, 0.04] | 0.122 | 1.000 | 0.03 | 16.35 | 82 [53, 95] | 0 | 0 |
| IL-6 | 31 | 31 | 10,422 | *0.10 [0.00, 0.20]* | *0.054* | 1.000 | 0.19 | 22.70 | 71 [42, 84] | 26 [0, 45] | 3 [0, 18] |
| TNF-α | 18 | 18 | 5,007 | 0.20 [-0.08, 0.49] | 0.148 | 1.000 | 0.24 | 13.76 | 78 [0, 94] | 39 [0, 72] | 0 |
| IL-1β | 11 | 11 | 2,708 | 0.41 [-0.36, 1.18] | 0.256 | 1.000 | 0.49 | 9.32 | 64 [0, 91] | 27 [0, 45] | 0 |
| Fibrinogen | 2 | 2 | 1,055 | 0.30 [-4.30, 4.90] | 0.556 | 1.000 | 0.51 | 1.00 | 100 | 100 | 0 |
|  |  |  |  |  |  |  |  |  |  |  |  |
| Males |  |  |  |  |  |  |  |  |  |  |  |
| All Markers | 62 | 94 | 198,689 | **0.15 [0.09, 0.22]** | **<.001** | **<.001** | 0.11 | 40.42 | 93 [82, 97] | 30 [7, 46] | 0 |
| CRP | 47 | 49 | 172,555 | **0.08 [0.05, 0.12]** | **<.001** | **0.001** | 0.06 | 26.12 | 98 [82, 100] | 12 [0, 43] | 0 |
| IL-6 | 24 | 24 | 16,564 | **0.16 [0.05, 0.27]** | **0.005** | **0.148** | 0.19 | 18.15 | 88 [58, 96] | 42 [0, 67] | 0 |
| TNF-α | 11 | 11 | 6,083 | 0.30 [-0.37, 0.98] | 0.337 | 1.000 | 0.40 | 9.18 | 73 [0, 100] | 27 [0, 64] | 0 |
| IL-1β | 6 | 6 | 1,905 | 1.84 [-1.10, 4.78] | 0.168 | 1.000 | 1.33 | 4.95 | 100 | 100 | 0 |
| Fibrinogen | 4 | 4 | 1,582 | 0.53 [-0.32, 1.37] | 0.140 | 1.000 | 0.47 | 2.94 | 100 | 100 | 0 |

***Note.*** k_pap._ = number of papers contributing to each model; k_est._. = number of total estimates (some papers contributed more than one estimate to each model); n_obs._ = total sample size (number of individual observations in each model). *p_Bon_****.*** *=* Bonferroni adjusted p value. %’s refer to the estimated percentage of population effects. Results in bold indicate *p* <0.05 and italics indicate 0.05≤*p<0.10*.

#### Table S15. Meta-analytic results for longitudinal studies: depression-biomarker associations separately among females and males, restricted to non-medical illness samples

|  | k_pap._ | k_est._ | n_obs._ | Cohen’s *d* [95% CI] | p | p_Bon._ | τ^2^ | df | % positive | % positive and *d*>.20 | % negative and *d*<-.20 |
| --- | --- | --- | --- | --- | --- | --- | --- | --- | --- | --- | --- |
| Depression and subsequent Inflammation | | | | | | | | | | | |
| Females |  |  |  |  |  |  |  |  |  |  |  |
| All Markers | 6 | 9 | 14,083 | 0.08 [-0.14, 0.31] | 0.381 | 1.000 | 0.12 | 4.93 | 100 | 0 | 0 |
| CRP | 6 | 6 | 12,492 | 0.07 [-0.12, 0.27] | 0.382 | 1.000 | 0.11 | 4.90 | 100 | 0 | 0 |
| IL-6 | 2 | 2 | 1,388 | 0.32 [-3.35, 3.99] | 0.467 | 1.000 | 0.41 | 1.00 | 100 | 100 | 0 |
|  |  |  |  |  |  |  |  |  |  |  |  |
| Males |  |  |  |  |  |  |  |  |  |  |  |
| All Markers | 7 | 15 | 10,602 | 0.04 [-0.02, 0.11] | 0.149 | 0.595 | 0.09 | 5.83 | 60 [0, 80] | 13 [0, 27] | 0 |
| CRP | 7 | 7 | 5,006 | 0.10 [-0.03, 0.22] | 0.109 | 1.000 | 0.11 | 5.90 | 100 | 0 | 0 |
| IL-6 | 5 | 5 | 4,022 | 0.01 [-0.08, 0.11] | 0.702 | 1.000 | 0.05 | 3.62 | 100 | 0 | 0 |
| Inflammation and subsequent Depression | | | | | | | | | | | |
| Females |  |  |  |  |  |  |  |  |  |  |  |
| All Markers | 12 | 17 | 29,624 | **0.03 [0.00, 0.06]** | **0.039** | 0.156 | 0.03 | 6.59 | 94 [59, 100] | 0 | 0 |
| CRP | 11 | 11 | 24,702 | 0.02 [-0.01, 0.04] | 0.122 | 1.000 | 0.02 | 5.86 | 91 [0, 100] | 0 | 0 |
| IL-6 | 5 | 5 | 4,824 | **0.05 [0.00, 0.10]** | **0.040** | 0.524 | 0.00 | 2.42 | 100 | 0 | 0 |
|  |  |  |  |  |  |  |  |  |  |  |  |
| Males |  |  |  |  |  |  |  |  |  |  |  |
| All Markers | 10 | 17 | 19,516 | 0.04 [-0.02, 0.10] | 0.154 | 0.617 | 0.06 | 5.69 | 82 [0, 100] | 0 | 0 |
| CRP | 9 | 9 | 13,729 | *0.04 [0.00, 0.08]* | *0.053* | 0.691 | 0.03 | 3.20 | 100 | 0 | 0 |
| IL-6 | 6 | 6 | 4,568 | 0.03 [-0.05, 0.10] | 0.388 | 1.000 | 0.05 | 3.84 | 100 | 0 | 0 |

***Note.*** k_pap._ = number of papers contributing to each model; k_est._. = number of total estimates (some papers contributed more than one estimate to each model); n_obs._ = total sample size (number of individual observations in each model). *p_Bon_****.*** *=* Bonferroni adjusted p value. %’s refer to the estimated percentage of population effects. Results in bold indicate *p* <0.05 and italics indicate 0.05≤*p<0.10*.

#### Table S16. Meta-analytic results for cross-sectional studies: depression-biomarker associations separately among females and males, restricted to samples assessing clinical depression diagnosis

|  | k_pap._ | k_est._ | n_obs._ | Cohen’s *d* [95% CI] | p | p_Bon._ | τ^2^ | df | % positive | % positive and *d*>.20 | % negative and *d*<-.20 |
| --- | --- | --- | --- | --- | --- | --- | --- | --- | --- | --- | --- |
| Females |  |  |  |  |  |  |  |  |  |  |  |
| All Markers | 24 | 47 | 60,476 | **0.33 [0.00, 0.67]** | **0.049** | 0.196 | 0.39 | 20.95 | 81 [60, 96] | 32 [0, 53] | 0 |
| CRP | 14 | 16 | 51,331 | *0.13 [-0.01, 0.27]* | *0.063* | 1.000 | 0.16 | 10.30 | 88 [0, 94] | 44 [0, 75] | 0 |
| IL-6 | 14 | 14 | 3,601 | 0.03 [-0.08, 0.13] | 0.584 | 1.000 | 0.10 | 5.64 | 64 [0, 93] | 7 [0, 57] | 7 [0, 43] |
| TNF-α | 9 | 9 | 3,297 | 0.64 [-0.05, 1.77] | 0.228 | 1.000 | 0.40 | 7.05 | 100 | 100 | 0 |
| IL-1β | 7 | 7 | 2,064 | 0.15 [-0.27, 0.57] | 0.394 | 1.000 | 0.29 | 4.59 | 100 | 0 | 0 |
|  |  |  |  |  |  |  |  |  |  |  |  |
| Males |  |  |  |  |  |  |  |  |  |  |  |
| All Markers | 21 | 34 | 49,925 | **0.78 [0.05, 1.51]** | **0.037** | 0.149 | 0.72 | 19.43 | 82 [57, 91] | 50 [26, 65] | 3 [0, 9] |
| CRP | 13 | 14 | 41,709 | *0.65 [-0.11, 1.41]* | *0.088* | 1.000 | 0.63 | 11.58 | 79 [0, 93] | 50 [0, 71] | 0 |
| IL-6 | 11 | 11 | 3,344 | 0.34 [-0.16, 0.83] | 0.158 | 1.000 | 0.43 | 8.83 | 73 [0, 91] | 55 [0, 73] | 9 [0, 36] |
| TNF-α | 4 | 4 | 2,328 | 1.81 [-3.70, 7.31] | 0.369 | 1.000 | 0.97 | 2.93 | 100 | 100 | 0 |
| IL-1β | 4 | 4 | 1,743 | 1.43 [-2.85, 5.71] | 0.364 | 1.000 | 1.40 | 2.97 | 100 | 100 | 0 |

***Note.*** k_pap._ = number of papers contributing to each model; k_est._. = number of total estimates (some papers contributed more than one estimate to each model); n_obs._ = total sample size (number of individual observations in each model). *p_Bon_****.*** *=* Bonferroni adjusted p value. %’s refer to the estimated percentage of population effects. Results in bold indicate *p* <0.05 and italics indicate 0.05≤*p<0.10*.

#### Table S17. Meta-analytic results for longitudinal studies: depression-biomarker associations separately among females and males, restricted to samples assessing clinical depression diagnosis

|  | k_pap._ | k_est._ | n_obs._ | Cohen’s *d* [95% CI] | p | p_Bon._ | τ^2^ | df | % positive | % positive and *d*>.20 | % negative and *d*<-.20 |
| --- | --- | --- | --- | --- | --- | --- | --- | --- | --- | --- | --- |
| Inflammation and subsequent Depression | | | | | | | | | | | |
| Females |  |  |  |  |  |  |  |  |  |  |  |
| All Markers | 2 | 2 | 1,228 | 0.06 [-0.08, 0.20] | 0.119 | 0.831 | 0.00 | 1.00 | 100 | 0 | 0 |

***Note.*** k_pap._ = number of papers contributing to each model; k_est._. = number of total estimates (some papers contributed more than one estimate to each model); n_obs._ = total sample size (number of individual observations in each model). *p_Bon_****.*** *=* Bonferroni adjusted p value. %’s refer to the estimated percentage of population effects. Results in bold indicate *p* <0.05 and italics indicate 0.05≤*p<0.10*.

#### Table S18. Meta-analytic results for cross-sectional studies: depression-biomarker associations separately among females and males, restricted to samples NOT assessing clinical depression diagnoses

|  | k_pap._ | k_est._ | n_obs._ | Cohen’s *d* [95% CI] | p | p_Bon._ | τ^2^ | df | % positive | % positive and *d*>.20 | % negative and *d*<-.20 |
| --- | --- | --- | --- | --- | --- | --- | --- | --- | --- | --- | --- |
| Females |  |  |  |  |  |  |  |  |  |  |  |
| All Markers | 61 | 100 | 151,450 | **0.05 [0.02, 0.08]** | **0.005** | **0.021** | 0.06 | 35.93 | 93 [80, 100] | 1 [0, 15] | 0 |
| CRP | 46 | 53 | 139,534 | 0.01 [-0.01, 0.03] | 0.169 | 1.000 | 0.01 | 5.02 | 92 [78, 100] | 0 | 0 |
| IL-6 | 26 | 27 | 7,964 | **0.12 [0.02, 0.21]** | **0.019** | 0.343 | 0.23 | 21.91 | 81 [44, 93] | 15 [0, 33] | 0 |
| TNF-α | 13 | 14 | 2,253 | 0.02 [-0.03, 0.07] | 0.324 | 1.000 | 0.00 | 5.06 | 100 | 0 | 0 |
| IL-1β | 4 | 4 | 644 | 0.90 [-2.21, 4.02] | 0.422 | 1.000 | 0.81 | 2.98 | 100 | 100 | 0 |
| Fibrinogen | 2 | 2 | 1,055 | 0.30 [-4.30, 4.90] | 0.556 | 1.000 | 0.51 | 1.00 | 100 | 100 | 0 |
|  |  |  |  |  |  |  |  |  |  |  |  |
| Males |  |  |  |  |  |  |  |  |  |  |  |
| All Markers | 50 | 74 | 156,082 | **0.10 [0.06, 0.13]** | **<.001** | **<.001** | 0.08 | 32.69 | 91 [78, 97] | 16 [1, 35] | 0 |
| CRP | 42 | 44 | 134,766 | **0.06 [0.04, 0.08]** | **<.001** | **<.001** | 0.03 | 18.80 | 98 [84, 100] | 0 | 0 |
| IL-6 | 16 | 16 | 14,928 | **0.10 [0.03, 0.18]** | **0.011** | 0.205 | 0.15 | 12.38 | 88 [50, 94] | 25 [0, 56] | 0 |
| TNF-α | 7 | 7 | 3,755 | 0.01 [-0.08, 0.10] | 0.616 | 1.000 | 0.00 | 2.40 | 100 | 0 | 0 |
| IL-1β | 2 | 2 | 162 | 2.76 [-31.99, 37.52] | 0.497 | 1.000 | 3.85 | 1.00 | 100 | 100 | 0 |
| Fibrinogen | 5 | 5 | 2,471 | 0.37 [-0.22, 0.96] | 0.153 | 1.000 | 0.30 | 3.76 | 100 | 100 | 0 |

***Note.*** k_pap._ = number of papers contributing to each model; k_est._. = number of total estimates (some papers contributed more than one estimate to each model); n_obs._ = total sample size (number of individual observations in each model). *p_Bon_****.*** *=* Bonferroni adjusted p value. %’s refer to the estimated percentage of population effects. Results in bold indicate *p* <0.05 and italics indicate 0.05≤*p<0.10*.

#### Table S19. Meta-analytic results for longitudinal studies: depression-biomarker associations separately among females and males, restricted to samples NOT assessing clinical depression diagnoses

|  | k_pap._ | k_est._ | n_obs._ | Cohen’s *d* [95% CI] | p | p_Bon._ | τ^2^ | df | % positive | % positive and *d*>.20 | % negative and *d*<-.20 |
| --- | --- | --- | --- | --- | --- | --- | --- | --- | --- | --- | --- |
| Depression and subsequent Inflammation | | | | | | | | | | | |
| Females |  |  |  |  |  |  |  |  |  |  |  |
| All Markers | 5 | 7 | 13,033 | 0.00 [-0.01, 0.01] | 0.597 | 1.000 | 0.00 | 1.48 | 100 | 0 | 0 |
| CRP | 5 | 5 | 11,967 | 0.00 [-0.01, 0.01] | 0.925 | 1.000 | 0.00 | 1.50 | 0 | 0 | 0 |
|  |  |  |  |  |  |  |  |  |  |  |  |
| Males |  |  |  |  |  |  |  |  |  |  |  |
| All Markers | 6 | 13 | 9,716 | 0.03 [-0.04, 0.11] | 0.290 | 1.000 | 0.06 | 4.65 | 62 [0, 85] | 0 | 0 |
| CRP | 6 | 6 | 4,563 | 0.06 [-0.05, 0.18] | 0.225 | 1.000 | 0.09 | 4.80 | 100 | 0 | 0 |
| IL-6 | 4 | 4 | 3,579 | 0.04 [-0.07, 0.14] | 0.323 | 1.000 | 0.04 | 2.40 | 100 | 0 | 0 |
| Inflammation and subsequent Depression | | | | | | | | | | | |
| Females |  |  |  |  |  |  |  |  |  |  |  |
| All Markers | 10 | 15 | 28,396 | 0.02 [-0.01, 0.05] | 0.153 | 1.000 | 0.03 | 4.47 | 93 [0, 100] | 0 | 0 |
| CRP | 10 | 10 | 24,058 | 0.01 [-0.01, 0.03] | 0.225 | 1.000 | 0.02 | 4.90 | 80 [0, 100] | 0 | 0 |
| IL-6 | 4 | 4 | 4,240 | *0.04 [-0.01, 0.09]* | *0.083* | 0.582 | 0.00 | 2.09 | 100 | 0 | 0 |
|  |  |  |  |  |  |  |  |  |  |  |  |
| Males |  |  |  |  |  |  |  |  |  |  |  |
| All Markers | 10 | 17 | 19,533 | *0.09 [-0.01, 0.18]* | *0.063* | 0.443 | 0.08 | 6.62 | 88 [35, 100] | 0 | 0 |
| CRP | 10 | 10 | 14,043 | **0.06 [0.00, 0.13]** | **0.050** | 0.349 | 0.05 | 5.27 | 100 [30, 100] | 0 | 0 |
| IL-6 | 5 | 5 | 4,271 | 0.04 [-0.06, 0.14] | 0.281 | 1.000 | 0.05 | 2.88 | 100 | 0 | 0 |

***Note.*** k_pap._ = number of papers contributing to each model; k_est._. = number of total estimates (some papers contributed more than one estimate to each model); n_obs._ = total sample size (number of individual observations in each model). *p_Bon_****.*** *=* Bonferroni adjusted p value. %’s refer to the estimated percentage of population effects. Results in bold indicate *p* <0.05 and italics indicate 0.05≤*p<0.10*.

# Figure S1-S22: Forest plots

#### Figure S1: Forest plot of depression and all markers combined among females in cross-sectional studies (k_papers_=85; k_estimates_=147; n_observations_=211,926)

#### Figure S2: Forest plot of depression and IL-6 among females in cross-sectional studies (k_papers_=40; k_estimates_=41; n_observations_=11,565)

#### Figure S3: Forest plot of depression and TNF-α among females in cross-sectional studies (k_papers_=22; k_estimates_=23; n_observations_=5,550)

#### Figure S4: Forest plot of depression and IL-1β among females in cross-sectional studies (k_papers_=11; k_estimates_=11; n_observations_=2,708)

#### Figure S5: Forest plot of depression and fibrinogen among females in cross-sectional studies (k_papers_=3; k_estimates_=3; n_observations_=1,238)

#### Figure S6: Forest plot of depression and all markers combined among males in cross-sectional studies (k_papers_=71; k_estimates_=108; n_observations_=206,007)

#### Figure S7: Forest plot of depression and IL-6 among males in cross-sectional studies (k_papers_=27; k_estimates_=27; n_observations_=18,272)

#### Figure S8: Forest plot of depression and TNF-α among males in cross-sectional studies (k_papers_=11; k_estimates_=11; n_observations_=6,083)

#### Figure S9: Forest plot of depression and IL-1β among males in cross-sectional studies (k_papers_=6; k_estimates_=6; n_observations_=1,905)

#### Figure S10: Forest plot of depression and fibrinogen among males in cross-sectional studies (k_papers_=6; k_estimates_=6; n_observations_=3,272)

#### Figure S11: Forest plot of depression and all markers combined among females in longitudinal studies – depression and subsequent inflammation (k_papers_=6; k_estimates_=9; n_observations_=14,083)

#### Figure S12: Forest plot of depression and CRP among females in longitudinal studies – depression and subsequent inflammation (k_papers_=6; k_estimates_=6; n_observations_=12,492)

#### Figure S13: Forest plot of depression and IL-6 among females in longitudinal studies – depression and subsequent inflammation (k_papers_=2; k_estimates_=2; n_observations_=1,388)

#### Figure S14: Forest plot of depression and all markers combined among males in longitudinal studies – depression and subsequent inflammation (k_papers_=7; k_estimates_=15; n_observations_=10,602)

#### Figure S15: Forest plot of depression and CRP among males in longitudinal studies – depression and subsequent inflammation (k_papers_=7; k_estimates_=7; n_observations_=5,006)

#### Figure S16: Forest plot of depression and IL-6 among males in longitudinal studies – depression and subsequent inflammation (k_papers_=5; k_estimates_=5; n_observations_=4,022)

#### Figure S17: Forest plot of depression and all markers combined among females in longitudinal studies – inflammation and subsequent depression (k_papers_=12; k_estimates_=17; n_observations_=29,624)

#### Figure S18: Forest plot of depression and CRP among females in longitudinal studies – inflammation and subsequent depression (k_papers_=11; k_estimates_=11; n_observations_=24,702)

#### Figure S19: Forest plot of depression and IL-6 among females in longitudinal studies – inflammation and subsequent depression (k_papers_=5; k_estimates_=5; n_observations_=4,824)

#### Figure S20: Forest plot of depression and all markers combined among males in longitudinal studies – inflammation and subsequent depression (k_papers_=11; k_estimates_=18; n_observations_=19,830)

#### Figure S21: Forest plot of depression and CRP among males in longitudinal studies – inflammation and subsequent depression (k_papers_=10; k_estimates_=10; n_observations_=14,043)

#### Figure S22: Forest plot of depression and IL-6 among males in longitudinal studies – inflammation and subsequent depression (k_papers_=6; k_estimates_=6; n_observations_=4,568)

## Figure S23-S46: Significance Funnel Plots

#### Figure S23: Significance funnel plot of depression and all markers combined among females in cross-sectional studies

#### Figure S24: Significance funnel plot of depression and CRP among females in cross-sectional studies

#### Figure S25: Significance funnel plot of depression and IL-6 among females in cross-sectional studies

#### Figure S26: Significance funnel plot of depression and TNF-α among females in cross-sectional studies

#### Figure S27: Significance funnel plot of depression and IL-1β among females in cross-sectional studies

#### Figure S28: Significance funnel plot of depression and fibrinogen among females in cross-sectional studies

#### Figure S29: Significance funnel plot of depression and all markers combined among males in cross-sectional studies

#### Figure S30: Significance funnel plot of depression and CRP among males in cross-sectional studies

#### Figure S31: Significance funnel plot of depression and IL-6 among males in cross-sectional studies

#### Figure S32: Significance funnel plot of depression and TNF-α among males in cross-sectional studies

#### Figure S33: Significance funnel plot of depression and IL-1β among males in cross-sectional studies

#### Figure S34: Significance funnel plot of depression and fibrinogen among males in cross-sectional studies

#### Figure S35: Significance funnel plot of depression and all markers combined among females in longitudinal studies – depression and subsequent inflammation

#### Figure S36: Significance funnel plot of depression and CRP among females in longitudinal studies – depression and subsequent inflammation

#### Figure S37: Significance funnel plot of depression and IL-6 among females in longitudinal studies – depression and subsequent inflammation

#### Figure S38: Significance funnel plot of depression and all biomarkers combined among males in longitudinal studies – depression and subsequent inflammation

#### Figure S39: Significance funnel plot of depression and CRP among males in longitudinal studies – depression and subsequent inflammation

#### Figure S40: Significance funnel plot of depression and IL-6 among males in longitudinal studies – depression and subsequent inflammation

#### Figure S41: Significance funnel plot of depression and all biomarkers combined among females in longitudinal studies – inflammation and subsequent depression

#### Figure S42: Significance funnel plot of depression and CRP among females in longitudinal studies – inflammation and subsequent depression

#### Figure S43: Significance funnel plot of depression and IL-6 among females in longitudinal studies – inflammation and subsequent depression

#### Figure S44: Significance funnel plot of depression and all biomarkers combined among males in longitudinal studies – inflammation and subsequent depression

#### Figure S45: Significance funnel plot of depression and CRP among males in longitudinal studies – inflammation and subsequent depression

#### Figure S46: Significance funnel plot of depression and IL-6 among males in longitudinal studies – inflammation and subsequent depression

## References

1.	VanderWeele TJ. Optimal approximate conversions of odds ratios and hazard ratios to risk ratios. *Biometrics*. 2020;76(3):746-752. doi:10.1111/biom.13197

2.	Mathur MB, VanderWeele TJ. A Simple, Interpretable Conversion from Pearson’s Correlation to Cohen’s for d Continuous Exposures. *Epidemiol Camb Mass*. 2020;31(2):e16-e18. doi:10.1097/EDE.0000000000001105

3.	Hasselblad V, Hedges LV. Meta-analysis of screening and diagnostic tests. *Psychol Bull*. 1995;117(1):167-178. doi:10.1037/0033-2909.117.1.167

4.	Chinn S. A simple method for converting an odds ratio to effect size for use in meta-analysis. *Stat Med*. 2000;19(22):3127-3131. doi:10.1002/1097-0258(20001130)19:22<3127::aid-sim784>3.0.co;2-m

5.	Weir CJ, Butcher I, Assi V, et al. Dealing with missing standard deviation and mean values in meta-analysis of continuous outcomes: A systematic review. *BMC Med Res Methodol*. Published online 2018. doi:10.1186/s12874-018-0483-0

6.	Pooled variance. In: *Wikipedia*. ; 2024. Accessed August 7, 2024. https://en.wikipedia.org/w/index.php?title=Pooled_variance&oldid=1234706247#Aggregation_of_standard_deviation_data

7.	Dippo CS, Wolter KM. *A Comparison of Variance Estimators Using the Taylor Series Approximation*. US Census Bureau; 1984. Accessed August 7, 2024. https://www.census.gov/library/working-papers/1984/adrm/rr84-21.html

8.	Hedges LV, Tipton E, Johnson MC. Robust variance estimation in meta-regression with dependent effect size estimates. *Res Synth Methods*. 2010;1(1):39-65. doi:10.1002/jrsm.5

9.	Fisher Z, Tipton E. robumeta: An R-package for robust variance estimation in meta-analysis. Published online March 7, 2015. doi:10.48550/arXiv.1503.02220

10.	Moriarity DP, Ellman LM, Coe CL, Olino TM, Alloy LB. A physiometric investigation of inflammatory composites: Comparison of “a priori” aggregates, empirically-identified factors, and individual proteins. *Brain Behav Immun - Health*. 2021;18:100391. doi:10.1016/j.bbih.2021.100391

11.	Lindqvist D, Wolkowitz OM, Mellon S, et al. Proinflammatory milieu in combat-related PTSD is independent of depression and early life stress. *Brain Behav Immun*. 2014;42:81-88. doi:10.1016/j.bbi.2014.06.003

12.	van Dooren FEP, Schram MT, Schalkwijk CG, et al. Associations of low grade inflammation and endothelial dysfunction with depression - The Maastricht Study. *Brain Behav Immun*. 2016;56:390-396. doi:10.1016/j.bbi.2016.03.004

13.	Shields GS, Spahr CM, Slavich GM. Psychosocial Interventions and Immune System Function: A Systematic Review and Meta-analysis of Randomized Clinical Trials. *JAMA Psychiatry*. 2020;77(10):1031-1043. doi:10.1001/jamapsychiatry.2020.0431

14.	Holm S. A Simple Sequentially Rejective Multiple Test Procedure. *Scand J Stat*. 1979;6(2):65-70.

15.	Mathur MB, VanderWeele TJ. New metrics for meta-analyses of heterogeneous effects. *Stat Med*. 2019;38(8):1336-1342. doi:10.1002/sim.8057

16.	Mathur MB, VanderWeele TJ. Robust Metrics and Sensitivity Analyses for Meta-analyses of Heterogeneous Effects. *Epidemiol Camb Mass*. 2020;31(3):356-358. doi:10.1097/EDE.0000000000001180

17.	Mathur MB, VanderWeele TJ. Sensitivity Analysis for Unmeasured Confounding in Meta-Analyses. *J Am Stat Assoc*. 2020;115(529):163-172. doi:10.1080/01621459.2018.1529598

18.	VanderWeele TJ, Ding P. Sensitivity Analysis in Observational Research: Introducing the E-Value. *Ann Intern Med*. 2017;167(4):268-274. doi:10.7326/M16-2607

19.	Vevea JL, Hedges LV. A general linear model for estimating effect size in the presence of publication bias. *Psychometrika*. 1995;60(3):419-435. doi:10.1007/BF02294384

20.	Slim K, Nini E, Forestier D, Kwiatkowski F, Panis Y, Chipponi J. Methodological index for non-randomized studies (minors): development and validation of a new instrument. *ANZ J Surg*. 2003;73(9):712-716. doi:10.1046/j.1445-2197.2003.02748.x
